# Deriving general structure–activity/selectivity relationship patterns for different subfamilies of cyclin-dependent kinase inhibitors using machine learning methods

**DOI:** 10.1038/s41598-024-66173-z

**Published:** 2024-07-03

**Authors:** Sara Kaveh, Ahmad Mani-Varnosfaderani, Marzieh Sadat Neiband

**Affiliations:** 1 Chemometrics and Cheminformatics Laboratory, Department of Analytical Chemistry, Tarbiat Modares University, Tehran, Iran; 2 Department of Chemistry, Payame Noor University (PNU), P.O. Box 19395-4697, Tehran, Iran

**Keywords:** Kinases, Cheminformatics, Chemical libraries, Cheminformatics, Computational chemistry, Drug discovery and development, Lead optimization, Screening

## Abstract

Cyclin-dependent kinases (CDKs) play essential roles in regulating the cell cycle and are among the most critical targets for cancer therapy and drug discovery. The primary objective of this research is to derive general structure–activity relationship (SAR) patterns for modeling the selectivity and activity levels of CDK inhibitors using machine learning methods. To accomplish this, 8592 small molecules with different binding affinities to CDK1, CDK2, CDK4, CDK5, and CDK9 were collected from Binding DB, and a diverse set of descriptors was calculated for each molecule. The supervised Kohonen networks (SKN) and counter propagation artificial neural networks (CPANN) models were trained to predict the activity levels and therapeutic targets of the molecules. The validity of models was confirmed through tenfold cross-validation and external test sets. Using selected sets of molecular descriptors (e.g. hydrophilicity and total polar surface area) we derived activity and selectivity maps to elucidate local regions in chemical space for active and selective CDK inhibitors. The SKN models exhibited prediction accuracies ranging from 0.75 to 0.94 for the external test sets. The developed multivariate classifiers were used for ligand-based virtual screening of 2 million random molecules of the PubChem database, yielding areas under the receiver operating characteristic curves ranging from 0.72 to 1.00 for the SKN model. Considering the persistent challenge of achieving CDK selectivity, this research significantly contributes to addressing the issue and underscores the paramount importance of developing drugs with minimized side effects.

## Introduction

Cyclin-dependent kinases (CDKs) encompass a versatile class of enzymes with the capacity to intricately modify distinct protein substrates closely associated with cell cycle progression^1^. Specifically, CDKs facilitate substrate phosphorylation by transferring phosphate groups from adenosine triphosphate (ATP) to specific amino acid residues, initiating precise cellular responses^1^. Additionally, CDKs play pivotal roles in numerous biological processes, including metabolism, apoptosis, cell signaling, permeation, cell migration, and various other pathways of cellular reactivity^2^. The biological significance of CDKs is evident in the treatment of various diseases, as indicated by the approval of 73 small molecules by the United States Food and Drug Administration (U.S. FDA) until 2022^3^. For instance, three drugs including Palbociclib, Ribociclib, and Abemaciclib are selective CDK4/6 inhibitors and are used for treating HER2+/HR advanced breast cancer and have been approved by the FDA in 2017^4^.

The CDK family is divided into three subgroups based on their interaction with cyclins. The first group includes CDK1–CDK4, and CDK6, which play distinct roles in cell cycle transitions, and cell division. The second group includes CDK7–CDK13 and CDK19 and CDK20 which regulate the transcription process in the cells. The third group comprises of CDK14–CDK18 and have particular motif sequences and lack specific biological functions^5,6^. CDK1 is very essential for cell cycle progression^6^ while the activity of CDK2 in the G2/M phase of cell division is much lower than CDK1^7^. CDK5 regulates the central nervous system^8^ and CDK9 is responsible for DNA damage, repair, and transcription^9,10^. As mentioned above, the distinct roles of various CDK subfamilies in biological processes highlight the importance of selective CDK inhibitors. For example, CDK5 plays a prominent role in the nervous system, while it has a minor effect in the endocrine and immune systems^8^. Therefore, selective CDK5 inhibitors can be used as effective drugs for treatment of diseases related to the nervous system with less side effects related to interventions with immune system^8,11^.

Recently, Akl et al. provided detailed information on the synthesis, characterization, and potential applications of novel piperazine-tethered phthalazines, serving as selective CDK1 inhibitors with in vitro anticancer activity against pancreatic cancer^12^. They identified a novel series of benzylphthalazine derivatives, which exhibited strong inhibition effect against CDK1^12^. Hassan et al. used 2D quantitative structure–activity relationship (QSAR) model to explore the structural features of pyrazolo[3,4-b] pyridines as CDK2 inhibitors^13^. They introduced a linear model which suggested three compounds with enhanced growth inhibition effect against two prevailing cancer types^13^. Daniels et al. discussed the potential role of CDK5 in various diseases, including Alzheimer, polycystic kidney, and neurodegenerative diseases^14^. They suggested a systematic approach for synthesis of the CDK5 inhibitors through a structure-based design in conjunction with diverse coupling methods such as the Buchwald method. The proposed approach had the potential to serve as a guiding framework for the development of additional highly selective inhibitors targeting alternative kinase enzymes^14^. Cidado et al. suggested AZD4573 compound, as a highly selective CDK9 inhibitor. They demonstrated that AZD4573 can effectively induce apoptosis in cancer cells by depleting MCL-1, highlighting its promising antitumor activity and supporting its evaluation in clinical trials^15^. In a recent study, Qian et al. introduced a deep learning framework called BiLAT that utilizes SMILES representation to predict the inhibitory activity of molecules on multiple CDK subtypes (CDK1, 2, 4, 5–9)^16^. This innovative model aimed to identify selective inhibitors targeting individual CDK subtypes or multitarget inhibitors tailored to specific subtypes^16^. In another recent study, Liang et al. devised a rapid method for identifying novel selective CDK4/6 kinase inhibitors^17^. Their approach yielded two new compounds that effectively inhibited CDK4/6 while maintaining excellent selectivity for CDK2, demonstrating promise in inhibiting retinoblastoma protein (pRb) phosphorylation in breast cancer cells and exhibiting potential for oral therapeutic applications in breast cancer^17^. Although the selectivity of CDKs inhibitors had been partially investigated in the aforementioned studies, a comprehensive and transparent view that explicitly correlates CDKs selectivity with molecular descriptors is conspicuously absent from the current literature. To fill this gap, our research endeavors to provide a holistic examination of SAR, uncovering potential correlations between compound selectivity for CDKs and various molecular descriptors. Moreover, we aim to identify distinct subspaces within the chemical space where different CDK inhibitors form discernible sub-clusters. Our work not only bridges existing knowledge gaps but also offers a more thorough and data-driven perspective on the selectivity of CDK inhibitors, paving the way for information-based targeted drug design.

The main aim of the present work is to infer and understand the general SAR patterns related to CDK inhibitors. To achieve this, we collected 12,704 active and inactive ligands of CDK1, CDK2, CDK4, CDK5, and CDK9 from the Binding DB. To assess the molecular characteristics of each ligand, a comprehensive range of molecular descriptors were computed^18^. Furthermore, we employed two machine learning methods, namely the counter propagation artificial neural network (CPANN)^19^ and supervised Kohonen networks (SKN)^20^ for classification of ligands based on their activities and therapeutic targets. The findings of this study demonstrate a clear correlation between the selected descriptors and the activity/selectivity of molecules, providing valuable insight that can aid medicinal chemists in developing drugs with improved efficacy and reduced side effects. In addition, the results of this research will help researchers to effectively design and optimize drug candidates, thereby streamlining the drug discovery and development process.

## Materials and methods

### Data sets

#### Binding DB

In this study, we collected 12,704 molecules in June 2021 from the Binding Database^21^. This dataset includes 1766, 2506, 3117, 2750, and 2565 molecules related to CDK1, CDK2, CDK4, CDK5, and CDK9 targets, respectively. Molecules of each class of compounds were downloaded as 3D structures in SDF format from the Binding DB. The IC_50_ thresholds used for labeling molecules into active and inactive groups have been established based on scientific evidence derived from previously published pharmaceutical articles. The selection of these thresholds was achieved by careful consideration of relevant references. As a next step, the molecules were categorized into active, and inactive according to the IC_50_ threshold values (see Supplementary Table S1). The range of molecules demonstrating intermediate activity, falling between active and inactive compounds, was designated as such but excluded from the scope of this study.

To prepare the molecules for analysis, several steps were taken. These steps included adding hydrogen atoms to the molecules, removing similar structures, generating 3D structures, and performing 3D optimization of molecular structures using the Merck Molecular Force Field (MMFF94) to determine partial atomic charges^20^. These data preparation steps were conducted using Open Babel software, version 2.4.0^22^. This careful optimization process involved a controlled iteration of up to 2500 steps, with a convergence threshold confidently set at 10^−6^ kcal mol^−1^. Ultimately, the files in SDF format were converted to HIN format. The optimized 3D structures in HIN formats were used as input to the DRAGON 5.5 software^18,23^ to calculate the molecular descriptors (see Supplementary Fig. S1a). A diverse set of 450 molecular descriptors was calculated for each molecule. The IC_50_ thresholds for group determination, as well as the number of molecules in active and inactive groups for each CDK target after the cleaning steps are presented in Supplementary Table S1. To ensure the elimination of duplicates within the dataset, a thorough examination was conducted across both the active and inactive groups, as well as within the active groups prior to model development. This meticulous process was implemented to guarantee the integrity and reliability of the dataset used in subsequent modeling procedures. After removing duplicated molecules and molecules with intermediate activity level, 8592 molecules were remained for further analysis. Subsequently, we employed a descriptor filtration code aimed at eliminating multicollinear variables exhibiting more than 90% similarity, as measured by the Pearson correlation coefficient^24^. From the pool of highly correlated variables, we selected those with the lowest average correlation to all other variables in dataset^24^. Subsequently, each molecular descriptor in the dataset underwent mean-centering and variance scaling^24^. This standardized scaling approach serves to eliminate bias in the models and equalize the variance among all variables^24^.

#### PubChem database

In this study, the models were evaluated through virtual screening on a dataset of 2 million molecules randomly selected from the PubChem database (PubChem-R)^25^. The downloading process was initiated by accessing the PubChem depository website where the molecules were stored in SDF files each containing 25,000 molecules. To achieve 2 million molecules, 80 files were randomly downloaded across the whole database (see Supplementary Fig. S1b). Prior to analysis, the collected molecular structures underwent optimization according to the strategy described in section “Binding DB” and a set of 450 molecular descriptors was calculated for each molecule.

The molecular weight (MW), total polar surface area (TPSA_(tot)_), and Moriguchi octanol–water partition coefficient (mLogP) values for the downloaded PubChem molecules fell within the ranges of 50 to 1269, 0 to 512, and − 12.31 to 11.35, respectively. The mean and standard deviation (STD) values for MW, TPSA_(tot)_, and mLogP were 351.39 (std = 102.38), 74.19 (std = 35.30), and 2.58 (std = 1.44), respectively. We utilized a graph plotting MW against TPSA_(tot)_ and employed the color spectrum to visually represent the ascending values of mLogP (see Supplementary Fig. S2). This graphical representation shows that the selected random subset is quite representative of the whole PubChem database and encompasses the molecules with broad ranges of mLogP, MW and TPSA values.

### Data set classification

The flowchart of the classification process in this work is presented in Supplementary Fig. S1a. The classification of molecules in this work was performed within two different strategies: (1) classification based on molecule’s activities, and (2) classification based on therapeutic targets. For the first strategy, we developed five models to distinguish active inhibitors from their inactive molecules for each CDK target, separately. As for the second strategy, we utilized active inhibitors of CDK1, CDK2, CDK4, CDK5, and CDK9 to train a multiclass-classification model. This multiclass model is valuable for distinguishing between five groups of active inhibitors and identifying important pharmacophores. The cleaned dataset includes 773 active and 688 inactive molecules for CDK1, 1440 active and 817 inactive molecules for CDK2, 875 active and 289 inactive molecules for CDK4, 650 active and 1195 inactive molecules for CDK5, and 1273 active and 592 inactive molecules for CDK9. The .csv files for this cleaned data can be found in supplementary material section.

### Descriptor selection with the variable importance in projection (VIP) approach

The VIP method was employed for variable selection, in this work. The primary goal of the VIP approach is to identify the variables (features) that contribute the most to the observed variability in the target space of the dataset^26^. The calculation of VIP scores involves analyzing the contributions of each variable to the performance of the partial least squares (PLS) model, typically by considering factors such as component weights and the amount of explained variance^26^. Higher VIP scores indicate greater importance^26^. To select the number of optimized descriptors for each model in this work, classification models were built with 14 to 35 variables, and the model that exhibited the highest statistical performance was identified as the best model with the optimal number of variables. The CDK1, CDK2, CDK4, CDK5, and CDK9 active/inactive models were defined by 20, 23, 17, 25, and 21 descriptors, respectively. Additionally, the multiclass model based on the therapeutic target was developed by 31 VIP-selected descriptors.

### Model development

Supplementary Fig. S1c outlines the steps in model development procedure. After scaling the dataset it was randomly partitioned into 70% for training and cross-validation, and 30% for the test set^20^. In this study, to ensure that the test set data were not highly similar to the training set data, a similarity analysis was conducted between test set and training set molecules. The similarity values (based on Euclidean distance) between each molecule in test set and all molecules in the training set were calculated. Consequently, the most similar molecule in training set was identified, for each molecule in the test set. The distribution of the similarities between each test set member and its nearest member in the training set are given in Appendix A in supplementary material. Moreover, for all models developed in this work, the mean, and standard deviation of the maximum similarity values are presented in Appendix A of the supplementary materials. This analysis ensured that a considerable portion of the molecules in the test set are quite different from the training set molecules. This helps for validating the results of the models obtained in this work^27^. The selection of descriptors was carried out using VIP, as detailed in section “Model development”. Two machine learning techniques, CPANN and SKN, were then employed to construct classification models. The comprehensive theories behind the CPANN and SKN techniques can be found in the literature^28–32^. For the sake of brevity, we describe the methods in the next section, briefly.

#### CPANN and SKN models

The CPANN method combines supervised and unsupervised learning to perform classification and regression^33^. The network comprises two layers: the Kohonen input layer (competitive) and the output layer (Grossberg)^33^. The arrangement of the neurons involves precisely positioning one layer on top of the other, which leads to a direct correspondence between the Kohonen neurons and the output neurons^30^. When a new sample is introduced into the network, the closest neuron to the input vector is selected as the winning neuron^33^. The weights of all neurons in the input and output layers are then updated according to Eqs. (1) and (2), respectively^33^.

1 $$W_{ij}^{new} = w_{ij}^{old} + \eta (t) \cdot a(d_{j} - d_{c} ) \cdot (x_{i} - w_{ij}^{old} )$$

2 $$u_{ij}^{new} = u_{ij}^{old} + \eta (t) \cdot a(d_{j} - d_{c} ) \cdot (y_{i} - u_{ij}^{old} )$$

In these equations, *j* and *i* are indices used for neurons and weights within the network, while symbol *c* indicates the winning neuron^33^. For the input layer, the $w_{ij}^{old}$ and $w_{ij}^{new}$, are the weights of neuron *j* before and after modification, respectively^30,33^. Similarly, for the output layer, $u_{ij}^{old}$ and $u_{ij}^{new}$ represent the weights of neuron *j* before and after modifying the weights^30,33^. The *η(t)* denotes the learning rate, which is a function of iterations and gradually decreases as the algorithm progresses through subsequent iterations^33^. Here, (*d*_*j*_ − *d*_*c*_) represents the topological distance between neuron *j* and the winning neuron^30,33^. The term *a*(*d*_*j*_ − *d*_*c*_) shows the neighborhood function, where the value of *a* falls between 0 and 1^34^. The weights in both layers, which consist of the Kohonen layer and the output layer, are adjusted based on pairs of input and output vectors, denoted as *x* and *y*^33^*.* Upon completing the training process, the data samples tend to cluster within adjacent neurons in the vector space^33,35^. The Grossberg layer comprises weights corresponding to each class^33^. This layer of neurons shares structural similarities with the upper layer^33^. In the output layer, the number of weights in the neurons matches the number of classes in the target vector^33^.

The SKN creates a hybrid map, called *XY*_*Map*_, by combining the input map (*X*_*Map*_) and the output map (*Y*_*Map*_) using a self-supervised learning method^29^. The main purpose of SKN is to visualize the topology of the input samples and their corresponding output vector in the dimension-reduced maps^29^. This allows for easier interpretation and understanding of high-dimensional data^29^. In each training phase of the SKN algorithm, the map is treated as a competitive network^36^. The algorithm calculates the distance between each neuron in the map and the input vector using a distance function^29^. The neuron that has the smallest distance to the input data is considered as the best matching unit (BMU)^29^. The SKN model provides a way to classify and visualize high-dimensional data by creating a hybrid map that combines both input and output information^29^. There is a difference between CPANN and SKN models. The input and output layers remain distinct from each other in the CPANN while they are fused together in the SKN^37,38^. In this work, the number of neurons in squared toroidal space and epochs for SKN and CPANN models were determined through tenfold Venetian-Blinds cross-validation approach^37,38^.

### Model validation

The data set was randomly divided into 70% calibration (training) and 30% test set to validate the models^20^, as detailed in section “Model development”. The models were established using the training set compounds and were subsequently verified for prediction accuracy using the independent test set^20^. The stability and accuracy of the classification models were assessed using tenfold Venetian-Blinds cross-validation on the training set molecules^20^. The molecules within the test set were neither utilized during the development of the model nor the selection of important variables. To evaluate the statistical significance of all classifiers, various measures derived from the confusion matrix including specificity, sensitivity, precision, accuracy, and Matthew's correlation coefficient (MCC) were employed^20^. These evaluations were performed for the training process, cross-validation, and independent test sets. Ultimately, receiver operating characteristic (ROC) curves were created for SKN and CPANN models, to visually evaluate their validity and performance^20^. In this study, the robustness of the models was assessed through the implementation of the y-randomization test^39,40^. This test involved the random shuffling of the independent variable (y), followed by the construction of new models using the original data matrix^40^. It is anticipated that models built with a randomly shuffled y would demonstrate diminished predictive performance^40^. To ensure reliability, the y-randomization test was iterated 100 times, and the resulting mean error rates for the training, cross-validation, and test set were computed for the models. This rigorous approach provides valuable insights into the stability and predictive capability of the developed models.

### Meaningful tests

The Mann–Whitney *U*-test test was used to compare the distributions of selected molecular descriptors between active and inactive molecules^41^. This is a non-parametric test that does not require a priori assumption about the underlying data distributions^41^. Furthermore, the Kruskal–Wallis test was used to examine the disparities among the distribution of the selected molecular descriptors across five active CDK inhibitors^42^. These facilitated a comprehensive understanding of the relationship between molecular activity and the descriptors under investigation^42^.

### Application domain (AD)

The calculation of the AD for the models provides a comprehensive insight into the predictive reliability^43–45^. Several methods exist for calculating AD, but one of the most employed approaches involves determining the “leverage” values of the molecules^43–45^. The leverage method is a valuable tool for assessing the relative position of a molecule within the structural space^43–45^. This method provides insights to see if the model's predictions can be reliably extended to new molecules^43–45^. If a molecule falls within this domain, the predicted data are considered reliable because they're essentially interpolated by the model^45,46^. On the other hand, if a molecule falls outside this domain, the predicted data becomes unreliable due to model extrapolation^45,46^. Leverage quantifies the extent of extrapolation, indicating how far a molecule deviates from the model’s training set's structural centroid^45,46^. The leverage value for each compound is determined using the following equation^45,46^.

3 $$h_{i} = {\text{x}}_{{\text{i}}}^{{\text{t}}} \left( {{\text{X}}^{{\text{T}}} {\text{X}}} \right)^{ - 1} {\text{x}}_{{\text{i}}}$$

Here, **X** represents the descriptor matrix of the training set, and **x**_**i**_ is the descriptor row vector corresponding to the compound *i*. The quantitative determination of the model's domain limit is based on the critical threshold for leverage, which is calculated as follows^20^:

4 $$h^{*} = \frac{3(p + 1)}{n}$$

where, *n* represents the number of compounds in the training set, and p refers to the number of VIP-selected descriptors^20^. Compounds with leverage values greater than the calculated threshold value, denoted as h^*^ (*h* > h^*^), are identified as structurally influential or high-leverage compounds^20^. This approach helps identify compounds that significantly impact the analysis due to their structural characteristics^20^. In this work, we used the leverage approach for determining the number of molecules which fall within and outside of the AD of the models^20^.

### Virtual screening

As mentioned in the previous sections, to validate the developed models they were used for virtual screening of the random subset of the PubChem database including 2 million molecules. To achieve this, we added the active inhibitors of CDKs to the collected random subset of the PubChem database. Our aim was to retrieve active molecules through the utilization of classification models, and we conducted virtual screening accordingly. The evaluation of screening performance involved employing enrichment factors (EF), along with computing the area under the curve (AUC) values. EF_1%_ and EF_10%_ represent the number of actives found at the top 1% and 10% of the sorted PubChem database, divided by 1% and 10% of total actives in the whole database, respectively^24,47,48^.

### Software

The calculations were conducted using a computer system equipped with an Intel(R) Core (TM) i7-7700K CPU @ 4.2 GHz and 32 GB of random-access memory. All calculations reported in this study were performed using MATLAB 2018b^49^ and RStudio (desktop version: 2021.09.0 + 351)^50^. The CPANN and SKN models were executed in MATLAB with the Kohonen toolbox (version 4.4)^51,52^.

## Results and discussion

### Classification of compounds based on CDK inhibition activity

To discover the SAR patterns within the active and inactive sets of CDK1, CDK2, CDK4, CDK5, and CDK9 molecules, we developed five classification models using KNN, SVM, SKN and CPANN methods. These models classify the molecules into active and inactive groups and were constructed using the VIP-selected descriptors, effectively capturing the wide range of chemical diversity present in the dataset. The full names, brief definitions, and the subtypes of these descriptors are presented in Supplementary Tables S2–S6. The mean and standard deviation values within the active and inactive groups together with the Mann–Whitney *p*-values, for the selected descriptors are included in these Tables, as well. Most of the calculated *p*-values are below 0.05 and it shows the effectiveness of the VIP method in selecting discriminatory variables for developing binary active/inactive classifiers.

The statistical results for classification of molecules into active and inactive groups using CPANN and SKN models, along with the optimal parameters identified during the training, cross-validation, and testing processes, are concisely presented in Table 1. It is noteworthy that the statistical results for both CPANN and SKN methods are comparable and there is no superiority among them. The confusion matrices, applicability domains of the active/inactive classification models, and y-randomization results are presented in Tables S7, S8 and S9**,** respectively. The CPANN and SKN top maps together with the ROC curves and AUC values for five active/inactive classification models are shown in Supplementary Figs. S3 and S4, respectively. As can be seen, the similar molecules of the same activity classes were grouped together in the top maps and formed coherent classes. The trained SKN and CPANN models demonstrated logical separation between molecules based on their activity levels. High values of AUC were obtained (more than 0.87) which indicates the meaningful power of the developed models for discerning active molecules from inactive ones. It also shows the efficacy of the selected descriptors for building models. Moreover, the results on the ADs (see Table S8), together with the cross-validation, and the performance on an external validation test set collectively demonstrate the reliability of the predictions generated by the models in this section.

**Table 1:** The statistical results for the developed binary active/inactive classifiers for different CDK groups. The models were evaluated in terms of sensitivity, specificity, precision, accuracy, and Mathew-correlation coefficient (MCC).

Model	Class	Precision	Sensitivity	Specificity	Non-error rate	Accuracy	MCC*	AUC
		Training/cross validation/test	Training/cross validation/test	Training/cross validation/test	Training/cross validation/test	Training/cross validation/test	Training/cross validation/test	
CDK1								
SKN	Active	0.888/0.789/0.756	0.896/0.780/0.763	0.869/0.753/0.743	0.882/0.776/0.753	0.884/0.778/0.753	0.766/0.506/0.612	0.88
	Inactive	0.878/0.764/0.750	0.869/0.753/0.743	0.896/0.799/0.763				
CPANN	Active	0.858/0.780/0.787	0.918/0.854/0.875	0.824/0.721/0.752	0.871/0.788/0.814	0.875/0.793/0.815	0.749/0.583/0.633	0.96
	Inactive	0.896/0.810/0.851	0.825/0.721/0.752	0.918/0.854/0.875				
CDK2								
SKN	Active	0.943/0.872/0.872	0.960/0.898/0.915	0.895/0.762/0.775	0.928/0.830/0.845	0.937/0.849/0.862	0.863/0.669/0.703	0.93
	Inactive	0.927/0.805/0.845	0.895/0.762/0.775	0.961/0.898/0.915				
CPANN	Active	0.900/0.850/0.865	0.951/0.907/0.905	0.808/0.711/0.763	0.880/0.810/0.834	0.900/0.837/0.852	0.780/0.650/0.680	0.97
	Inactive	0.901/0810/0.828	0.808/0.711/0.763	0.951/0.907/0.905				
CDK4								
SKN	Active	0.966/0.946/0.947	0.977/0.959/0.976	0.893/0.826/0.844	0.935/0.893/0.910	0.957/0.927/0.942	0.881/0.800/0.847	0.94
	Inactive	0.926/0.866/0.927	0.893/0.826/0.844	0.977/0.959/0.976				
CPANN	Active	0.952/0.936/0.929	0.977/0.957/0.965	0.846/0.795/0.788	0.912/0.876/0.877	0.945/0.918/0.919	0.849/0.797/0.784	0.99
	Inactive	0.922/0.857/0.887	0.846/0.795/0.788	0.977/0.957/0.965				
CDK5								
SKN	Active	0.826/0.737/0.683	0.914/0.827/0.833	0.894/0.837/0.795	0.904/0.832/0.814	0.901/0.834/0.808	0.792/0.650/0.605	0.97
	Inactive	0.950/0.898/0.900	0.894/0.837/0.795	0.914/0.827/0.833				
CPANN	Active	0.825/0.741/0.672	0.921/0.807/0.781	0.893/0.845/0.798	0.907/0.826/0.789	0.903/0.831/0.792	0.796/0.641/0.562	0.97
	Inactive	0.953/0.888/0.873	0.893/0.845/0.798	0.921/0.807/0.781				
CDK9								
SKN	Active	0.931/0.904/0.875	0.946/0.908/0.886	0.849/0.791/0.733	0.897/0.849/0.809	0.915/0.871/0.837	0.803/0.701/0.624	0.90
	Inactive	0.879/0.799/0.754	0.849/0.791/0.733	0.946/0.908/0.886				
CPANN	Active	0.927/0.888/0.876	0.969/0.929/0.897	0.835/0.747/0.733	0.902/0.838/0.815	0.927/0.872/0.844	0.829/0.697/0.639	0.98

As shown in Table S9, the error rates observed during the y-randomization test for both CPANN and SKN model are notably high across the training, cross-validation, and test sets. Also, the average AUC values for y-randomization test have decreased compared to the original models. This finding substantiates that the favorable outcomes attained by these models do not stem from fortuitous correlation, thus affirming the reliability of the developed models. According to the aforementioned results, the descriptors chosen utilizing the VIP method could proficiently represent the structural characteristics of molecules in correlation to their activity levels. To visualize the abstract space represented by the VIP-selected descriptors for active/inactive models, principal component analysis (PCA) and t-distributed stochastic neighbor embedding (t-SNE) algorithms were conducted, and the results are presented in Supplementary Fig. S5. The first three PCs explain 33.1%, 28%, 47.5%, 32.6%, and 34.5% of the total variance of the selected descriptors, for CDK1, CDK2, CDK4, CDK5, and CDK9 groups, respectively. The first three principal components account for approximately 30% of the total variance in the data and reveal significant clustering patterns within this level of explained variance. The inspection of Supplementary Fig. S5a–e shows an acceptable distinction between active and inactive molecules in PC spaces. The dispersions of the molecules in the tSNE spaces show similar separation patterns. This separation is prominently evident in both PC and tSNE plots for the CDK2, CDK5, and CDK9 groups. The results obtained from these plots are consistent with the findings from the CPANN and SKN models, confirming that the selected descriptors encode valuable information about the activity levels of the molecules. For further validation, comparisons were made with other linear and non-linear models, including KNN and SVM, and the results are provided in the supplementary Table S10. It can be observed that the results obtained with SKN and CPANN surpass those of the KNN and SVM models.

### Common VIP descriptors in active/inactive classification models

**Figure 1:**
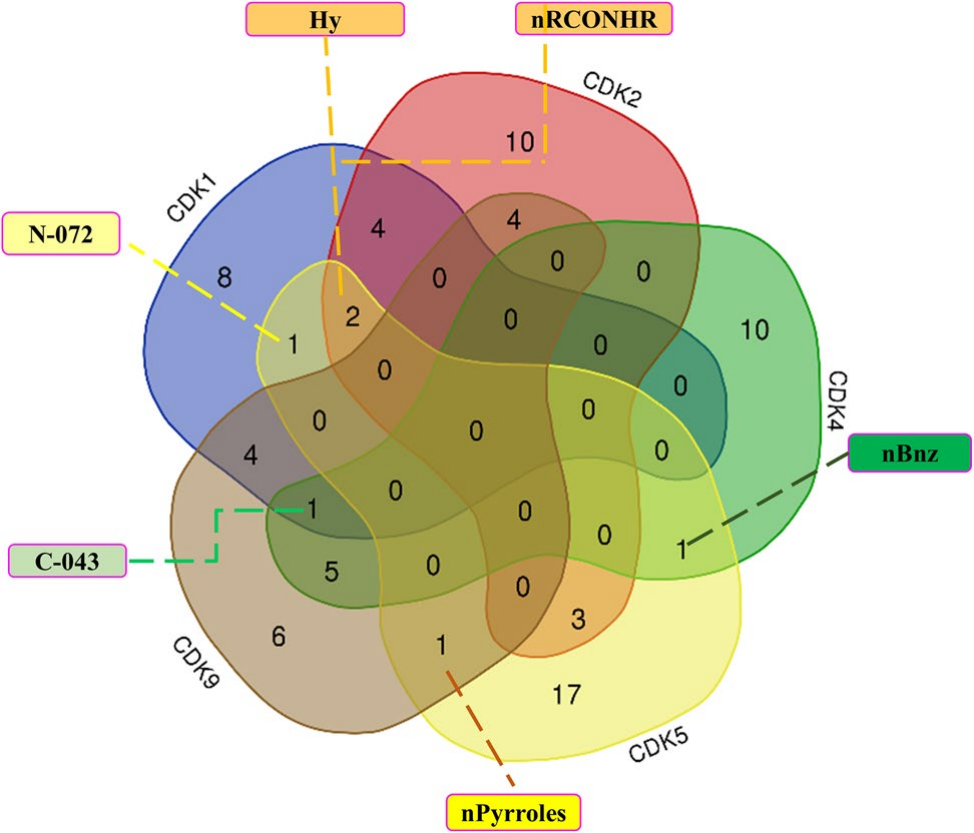
The Venn diagram of the VIP-selected descriptors used for developing active/inactive classifiers for five groups of CDK molecules: CDK1: blue, CDK2: crimson, CDK4: green, CDK5: yellow, CDK9: brown.

**Figure 2:**
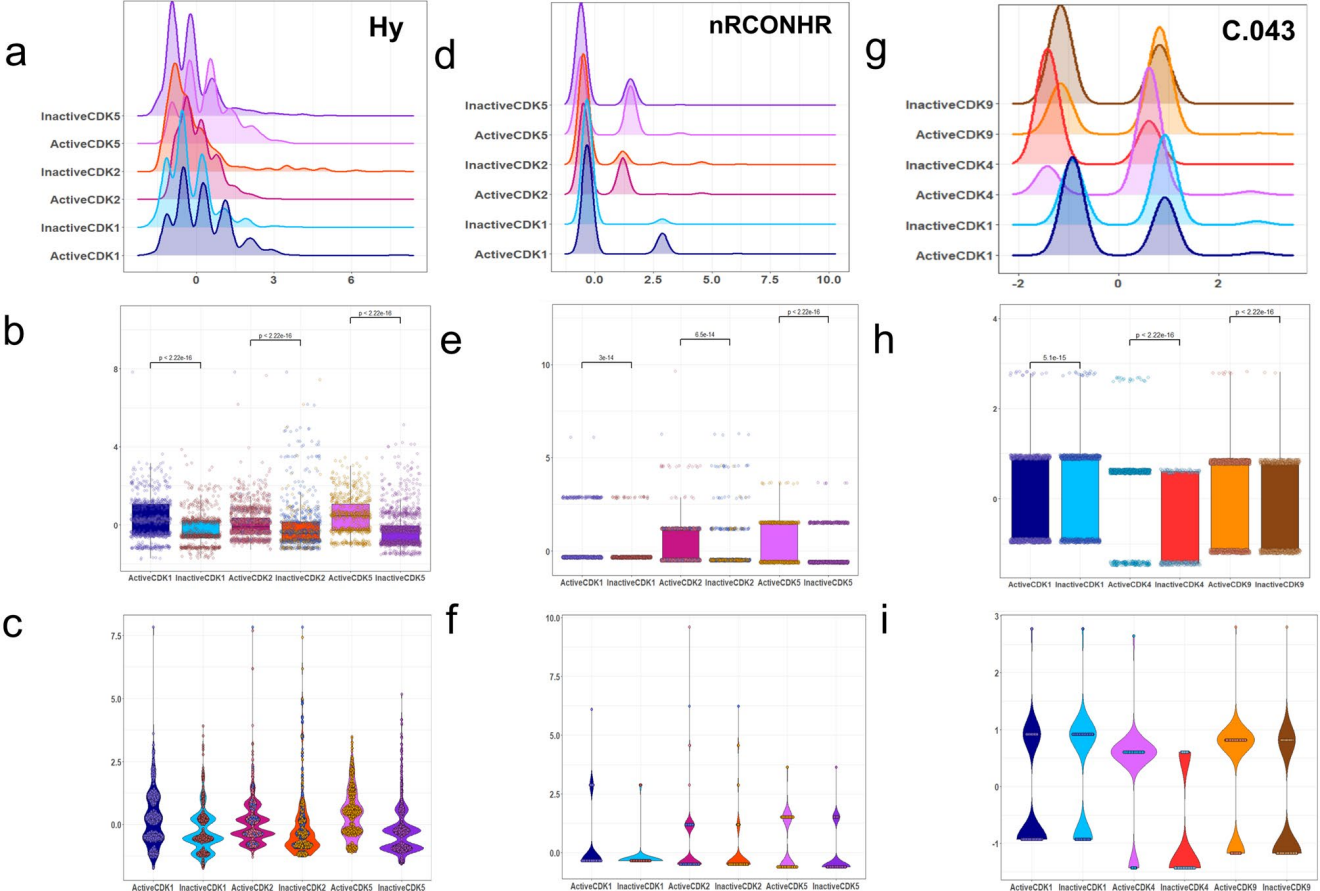
The density plot, box plot, and beeswarm plot (**a**–**c**) for Hy molecular descriptor for the active and inactive groups of CDK1, CDK2, and CDK5 molecules (**d**–**f**) for nRCONHR molecular descriptor for the active and inactive groups of CDK1, CDK2, and CDK5 molecules (**g**–**i**) for C-043 molecular descriptor for the active and inactive groups of CDK1, CDK4, and CDK9 molecules.

**Figure 3:**
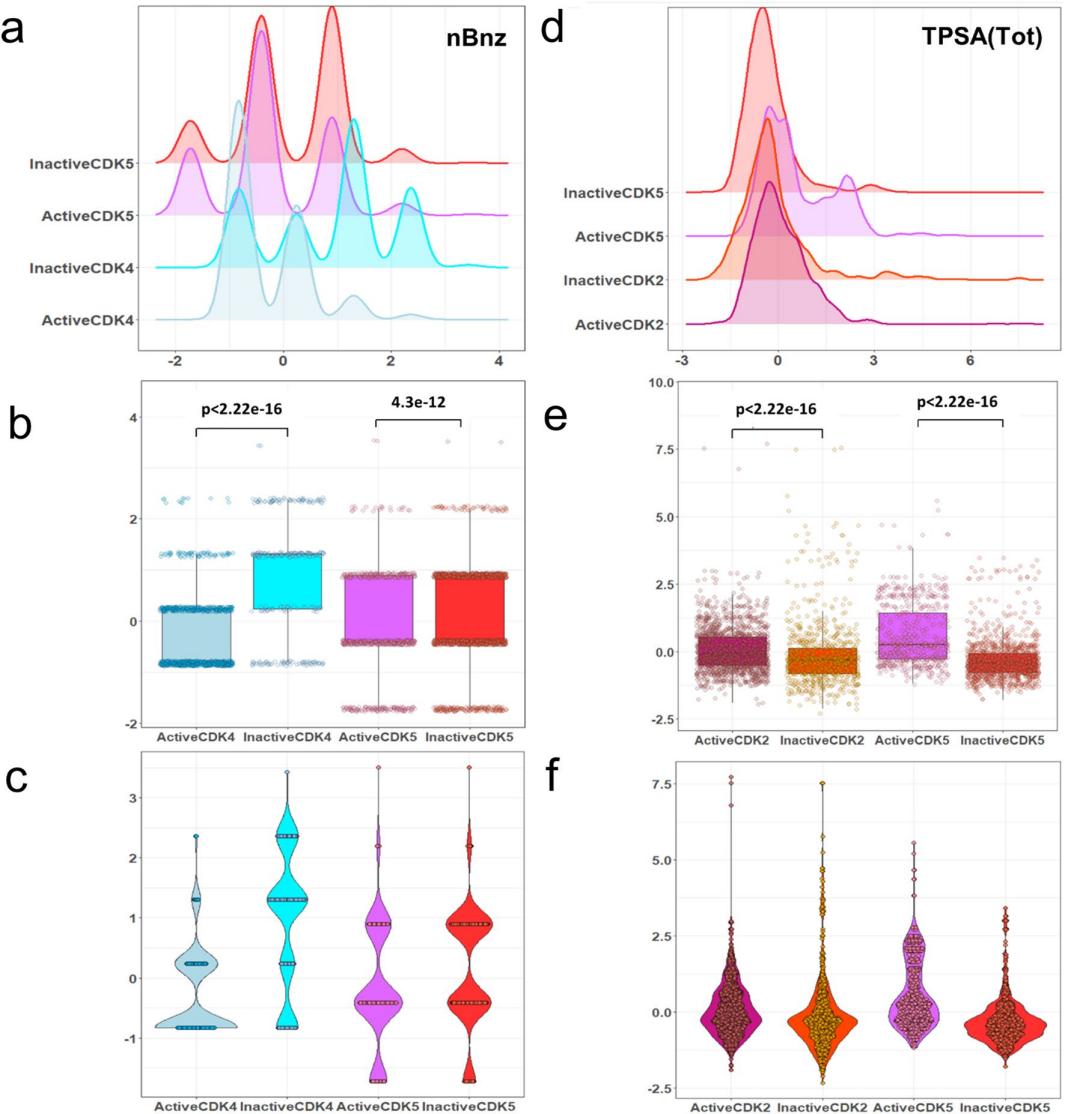
The density plot, box plot, and beeswarm plot (**a**–**c**) for nBnz molecular descriptor for the active and inactive groups of CDK4, and CDK5 molecules (**d**–**f**) for TPSA_(Tot)_ molecular descriptor for the active and inactive groups of CDK2 and CDK5 molecules.

As mentioned in the previous sections, we utilized the VIP approach to choose descriptors for the classification of active and inactive molecules within the five groups of CDK molecules. The presence of common descriptors across the discussed models implies their significance for describing CDK inhibitory activity. A Venn diagram showing common molecular descriptors for classifying active and inactive molecules for five groups of CDK molecules is shown in Fig. 1. The list of common descriptors between five active/inactive models are shown in Supplementary Table S11. As can be seen in Fig. 1 and Table S11, two molecular descriptors of Hy and nRCONHR appeared in active/inactive models for three groups of CDK1, CDK2, and CDK5. Hy represents the hydrophilic factor. This descriptor is defined by the following equation^53^:

5 $$Hy = \frac{{(1 + N_{Hy} ) \cdot log_{2} (1 + N_{Hy} ) + N_{C} \cdot \left( {\frac{1}{A} \cdot log_{2} \frac{1}{A}} \right) + \sqrt {\frac{{N_{Hy} }}{{A^{2} }}} }}{{log_{2} (1 + A)}}$$

where $N_{Hy}$ is the number of hydrophilic groups (–OH, –SH, –NH). $N_{C}$ is the number of carbon atoms and *A* is the number of hydrogen atoms^53^. The nRCONHR is the number of secondary amides (aliphatic)^54^. To gain a more comprehensive understanding of the distribution of the selected descriptors among different groups of active and inactive molecules, density, box, and beeswarm plots were employed. The distributions of the values of Hy across active and inactive molecules within the CDK1, CDK2, and CDK5 groups are shown in Fig. 2a–c. Upon examining this figure and Tables S2, S3, and S5, it becomes evident that the average values of Hy for the active molecules are higher than those of the inactive molecules for three CDK targets. This observation suggests that the increasing the value of this descriptor tends to shift molecules from an inactive to an active state. Qian et al. also reported in their recent study that an increase in this feature (Hy) is related to the activity of CDK inhibitors^16^. Figure 2d–f vividly illustrates the distribution of the values of nRCONHR within the active and inactive molecules for three groups of CDK1, CDK2, and CDK5 isoforms. Examining this figure and Tables S2, S3, S5 shows that average values of nRCONHR for active molecules are significantly higher than inactive molecules for the three mentioned targets. This observation suggests that the hydrophilic factor and the number of aliphatic amides of the second type may provide insights into designing active CDK1, CDK2, and CDK5 inhibitors. The molecular descriptor of C-043 appeared in three active/inactive models for CDK1, CDK4, and CDK9 groups. The C-043 shows the atom-centered fragment X--CR..X, where R shows any group linked through carbon, and X demonstrates any electronegative atom such as O, N, S, P, Se, and halogen^55^. In this notation, “--” denotes a single bond connecting the atom-centered fragment X with the central carbon atom (C), while “..” indicates a bond involving a delocalized or unspecified number of atoms or groups between the two X atoms^46,53^. Figure 2g–i illustrate the distribution of C-043 for CDK1, CDK4, and CDK9 active and inactive molecules. The average values of the C-043 descriptor are higher for active CDK4 and CDK9 inhibitors compared to their inactives, while the average value of this descriptor for the inactive CDK1 molecules is higher than those for active ones (see Fig. 2g–i and Tables S2, S4, and S6). This result suggest that this specific descriptor is likely to be effective in the design of selective CDK4 and CDK9 inhibitors. The descriptor nBnz, represents any aromatic rings, including both carbocyclic benzene and heterocyclic aromatic systems such as thiophene, pyridine, pyrazole, and others^53^. This descriptor was present in both active/inactive classification models for CDK4 and CDK5 isoforms. Examination of Fig. 3a–c and Tables S4 and S5 reveals that the numerical values of the nBnz are excessively high for inactive molecules of CDK4 and CDK5. Moreover, the mean values of this descriptor are comparatively lower for active inhibitors of CDK4 and CDK5 compared to inactive molecules. It suggests that increasing nBnz values may inversely impact the inhibition of CDK4 and CDK5 active molecules. In a recent research on CDK2 inhibitors, a benzene ring structure selected from Morgan fingerprints, showed a negative contribution to the model performance based on SHAP values for explaining the activity^16^. This emphasizes on the general importance of Benzene rings on the activity state of the CDK inhibitors and suggest lower benzene rings for better inhibition effect. The molecular descriptors TPSA_(Tot)_, O-060, and nArOR are common between the features of CDK2 and CDK5 active/inactive models. TPSA_(Tot)_ indicates the distribution of topological polar surface area using polar contributions N, O, S, P^56^. Figure 3d–f show the distribution of TPSA_(Tot)_ among the CDK2 and CDK5 active and inactive molecules. The significantly higher numerical values of TPSA_(Tot)_ in active CDK2 and CDK5 molecules suggest that this descriptor positively influences the activity of molecules for inhibiting these targets. Notably, a recent study had identified TPSA as an influential parameter for describing the activity of CDK2 inhibitors^16^. They showed that the TPSA, with an average positive SHAP value had a positive impact on the predicted outcome in the random forest (RF) and Deep Neural Network (DNN) models for modeling the activity of CDK2 inhibitors^16^. This is in harmony with our finding on higher average values of this descriptor within the active groups of CDK2 and CDK5 groups, compared to inactive ones. The density plot, box plot, and beeswarm plots of the distributions of the other common descriptors are shown in Figs. S6–S12 in the supplementary material.

To compare the ten groups of active and inactive molecules for CDK1, CDK2, CDK4, CDK5, and CDK9, the VIP-selected molecular descriptors were pooled from all five active/inactive classifiers, leading to the identification of a common set of 77 descriptors. The molecular descriptors for the ten groups were collected and scaled together. Scaling the data for all molecules together ensures a valid visualization since the molecules in newly scaled space can be compared together. The two- and three-dimensional PCA score and tSNE plots are shown in Supplementary Figs. S13 and S14, respectively. The first three PCs only explains 15.61% of the variance in the entire dataset, indicating a relatively low percentage of the variance coverage. A general view of the PCA plots shows overlapping between majority of the groups but detailed inspection exhibited reasonable clustering for some specific classes, including active molecules of CDK4, CDK5 and CDK9. In terms of the tSNE plots, the active CDK2, CDK4, and CDK9 groups show some distinct clustering patterns. The other groups of molecules do not exhibit suitable clustering patterns in both PCA and tSNE maps. This observation suggests a need for developing a distinct multiclass classifier for discriminating between active CDK inhibitors. This will lead to find the specific subspaces in chemical space in which the molecules will be clustered there based on their therapeutic targets. This modeling approach is discussed in subsequent section “Classification of compounds based on therapeutic targets”.

### Classification of compounds based on therapeutic targets

In this section, 5011 active inhibitors were classified into five groups based on their therapeutic targets. This data includes 773, 1440, 875, 650 and 1273 active molecules for CDK1, CDK2, CDK4, CDK5, and CDK9 targets. Common compounds were removed between different groups of active molecules, and the models were developed based on molecules uniquely associated with each target. We classified active inhibitors based on their therapeutic targets and developed four multi-class classification models using KNN, SVM, CPANN and SKN approaches. To construct these models, 31 descriptors were selected using the VIP method. Tables S12, S13, and S9 provide a comprehensive overview of the statistical results, confusion matrix, AD, and y-randomization results for the CPANN and SKN models. The result in Table S8 shows that most molecules are within the ADs of the models indicating the reliability of the prediction made by these models. Both SKN and CPANN classification methods achieved prediction accuracy values exceeding 0.80. Detailed inspection of the results shows that the SKN model achieved better accuracies compared to the CPANN model. The SKN top map shown in Fig. S15 illustrates the optimized SKN model for classifying active inhibitors. As can be seen in this figure, the active molecules within each class fall within the same group and all five groups show strong clustering patterns on the map. For further evaluation, the results of SKN and CPANN models were compared with those of KNN and SVM approaches (see Table S14). This shows superior performance of SKN and CPANN compared to those of KNN and SVM approaches.

**Figure 4:**
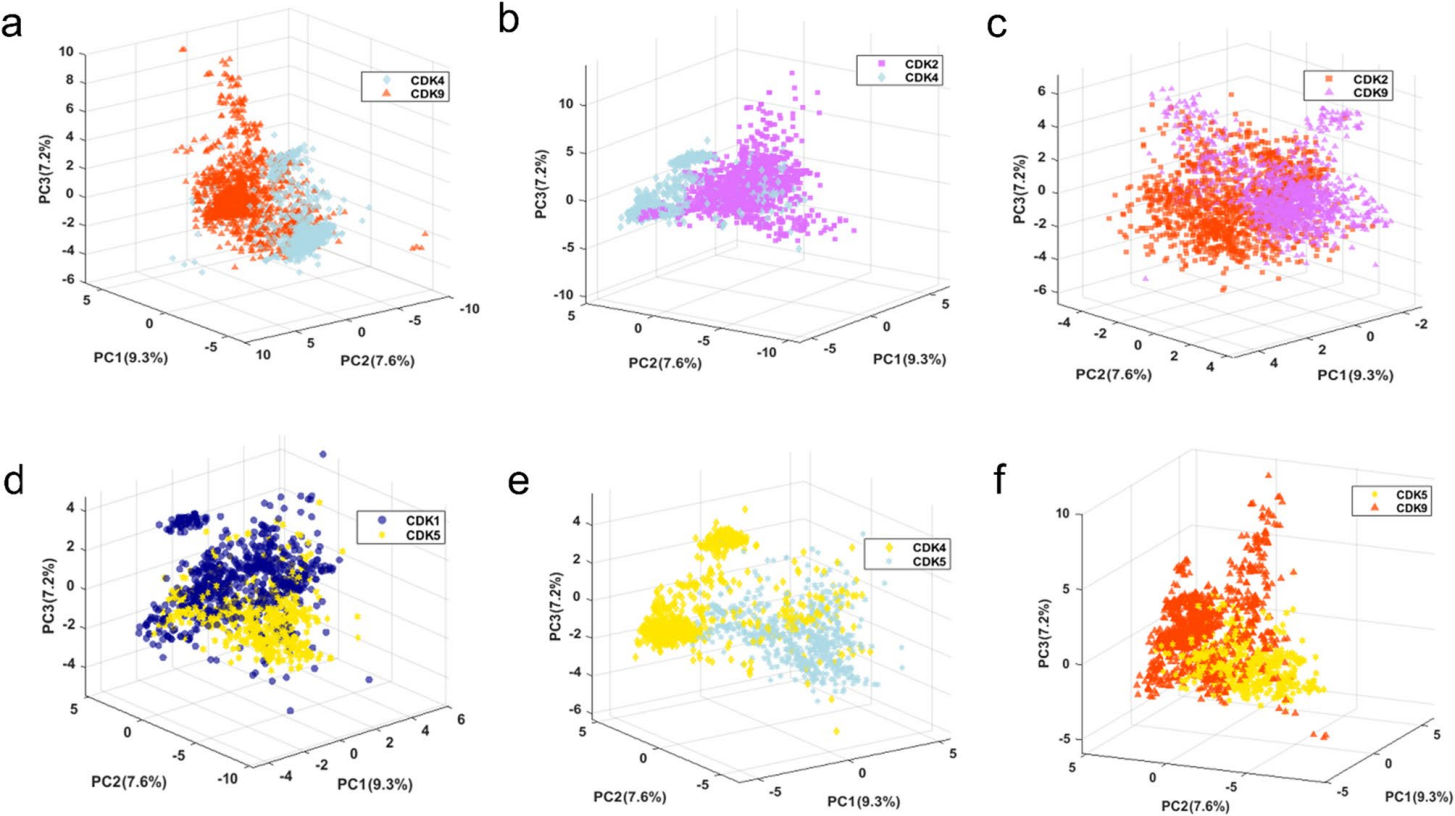
One-against-one visualization of the abstract PC space made by the 31 VIP-selected molecular descriptors used for multiclass classification of active CDK molecules (Dark blue: active CDK1, magenta: active CDK2, light blue: active CDK4, gold: active CDK5, orange red: active CDK9).

The abstract PCA space built using 31 VIP-selected descriptors is presented in Fig. S16. The first three PCs cover 24.1% of the whole variance of the selected descriptors. Since there are five groups of active molecules, it is hard to clearly see how they cluster in a three-dimensional score plot. To achieve clearer visualization, one-against-one PCA score plots for different active CDK inhibitors are provided in Fig. 4. Each section of this figure reveals clear separation between active CDK inhibitors in the subspace made by VIP-selected descriptors. Moreover, the tSNE plot using 31 descriptors is shown in Fig. S17. The active CDK4, CDK2 and CDK9 make clear clusters in the tSNE space of the 31 selected descriptors and a relative meaningful cluster is also seen for CDK1 group. The results of PCA and tSNE analysis on the selected descriptors suggest that the developed abstract space maps (i.e. PCA and tSNE) can be used for future drug design and synthesis projects as guiding tools to help the medicinal chemists focusing on compounds projected on specific cluster with desirable selectivity toward specific CDK targets.

### VIP selected descriptors in active classification models

**Figure 5:**
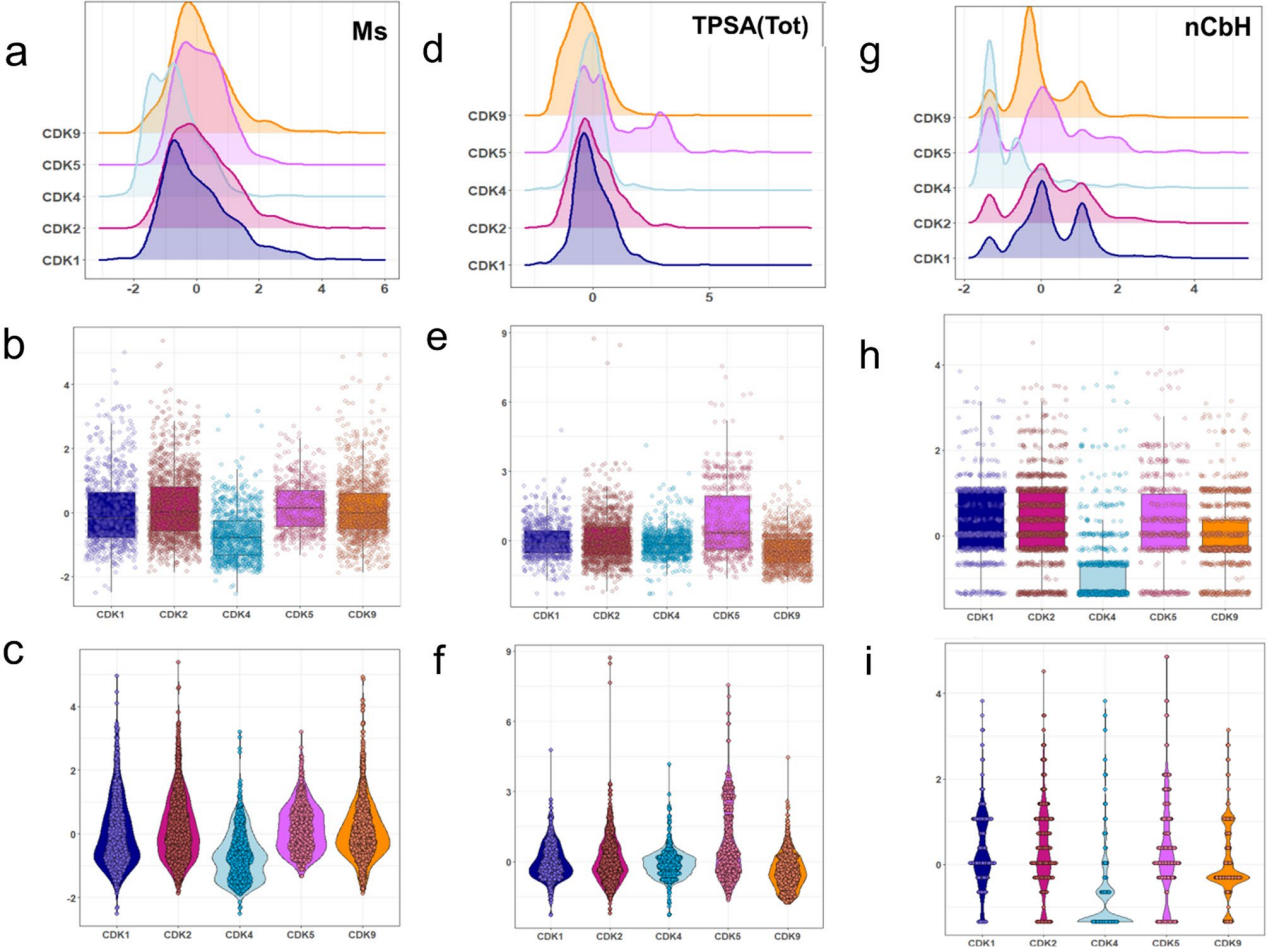
The density plot, box plot, and beeswarm plot (**a**–**c**) for Ms (**d**–**f**) for TPSA_(Tot)_ (**g**–**i**) for nCbH molecular descriptor(s) within five different groups of active CDK inhibitors.

The set of 31 VIP-selected descriptors for classification of active CDK inhibitors is provided in Table S15. This table comprises the full names, concise definitions, subtypes, mean values, standard deviations, and Kruskal–Wallis test *p*-values for these descriptors across five groups of active CDK inhibitors. Notably, all calculated *p*-values are below 0.05, showing the significant differences of the selected descriptors between five CDK groups. Among the 31 descriptors, the three descriptors with highest VIP values are described here and the distribution of the remaining features are presented in Supplementary Fig. S18. The first descriptor, Ms, represents the mean electrotopological state, which is associated with the polarity of atoms and steric accessibility. The distributions of this molecular descriptor in active inhibitors are shown in Fig. 5a–c. As can be seen in this figure, the distribution of the Ms for CDK4 group is skewed to lower values. Moreover, the distribution of this descriptor for CDK2 and CDK5 are inclined to higher values. This suggests that a molecule with high Ms value tends to potentially inhibit the CKD2 and CDK5 targets, while it is being less likely to inhibit a CDK4 target. As can be seen in the box plot (Fig. 5b), CDK2 and CDK9 have higher median values and CDK1 and CDK2 have wider interquartile ranges, indicating a greater variability in Ms values. The beeswarm plot (Fig. 5c) complements the box plot by displaying individual data points, avoiding overlap, and providing a clearer view of the data distribution. The next important descriptor is TPSA_(Tot)_. The distributions of this descriptor across the active CDK inhibitors are shown in Fig. 5d–f. The value of this molecular descriptor is meaningfully higher for CDK5 inhibitor compared to other groups. Moreover, the distribution of this descriptor for CDK9 group is intensely skewed to lower values. This suggests that increasing and decreasing the value of TPSA_(Tot)_ can potentially induce selectivity toward CDK5 and CDK9 targets, respectively. The next important descriptor is nCbH, representing the number of unsubstituted benzene rings. The nCbH descriptor exhibits higher values for the active CDK2 group compared to other groups. The positive correlation between this descriptor and activity highlights its significance in enhancing the effectiveness of the CDK2 active group, making it valuable for the design of selective drugs. The CDK2 and CDK4 model were further evaluated to assess the SAR patterns identified in this study. The detailed results of this evaluation are provided in Appendix B in the supplementary material.

Two distinct reports about the design of selective CDK inhibitors were found in the literature authored by Qian et al.^16^ and Liang et al.^17^. The former study described the BiLAT framework and compared it with machine learning models, including random forest (RF), SVM, and XGBoost, as well as deep learning models such as DNN, and Attentive FP^16^. Their work involved a dataset comprising 17,986 compounds targeting CDK1-2, CDK4, and CDK 5–9, sourced from PubChem, ChEMBL, and Binding DB^16^. They further improved model performance through data augmentation^16^. The study focused on 20 descriptors for CDK2, including hydrophobic and hydrophilic effects (SlogP_VSAk_), TPSA, and benzene ring structure (Morgan_389 and Morgan_1199)^16^. The research employed interpretation tools like self-attention module and SHAP method to visualize and elucidate key substructures affecting CDK inhibitor activity^16^. The results showed that the multitask BiLAT model outperformed the single-task version, with data augmentation proving beneficial for both single-task and multitask models^16^. In the later work, the primary objective was to rapidly identify selective CDK4/6 kinase inhibitors featuring a novel structural framework by ensuring effective target specificity while minimizing off-target effects^17^. The authors employed a multitask model, comprising a GENERATOR and a PREDICTOR, to create and evaluate potential compounds for their selectivity toward CDK4/6 kinase^17^. Using a dataset of 1000 known CDK4/6 inhibitors, the researchers trained and tested their models, achieving impressive results^17^. The study employed various algorithms, including support vector classification (SVC), RF, naive Bayes (NB), KNN, and multi-layer perceptron (MLP), within the PREDICTOR model to evaluate the potential activity of generated compounds^17^. Notably, the study identified seven molecules with high selectivity scores, highlighting the potential of this method to efficiently generate novel CDK4/6 inhibitors and aiding researchers in early-stage drug discovery^17^. Some VIP-selected descriptors in our work also appeared in previous studies (i.e. TPSA, benzene ring structure, hydrophobicity) emphasizing the invaluable roles of these parameters for describing activity/selectivity of CDK inhibitors. The major novelty of our work compared to previous studies relies on development of the abstract chemical spaces using selected descriptors as visual activity/selectivity maps. The molecules with different activity levels and therapeutic targets were distinctly clustered in these maps and it made it much easier to interpret the developed multivariate models. Moreover, we distinctly compared the distribution of the important descriptors across different groups of molecules using non-parametric significant tests, which was not achieved before. The activity/selectivity maps together with significant tests provided versatile tools for interpreting SKN and CPANN models in this work. Furthermore, our work explored the practical usage of the developed models for virtual screening of a random subset of PubChem database including ~ 2 million molecules. Screening of such a large database was not reported by Qian et al. and Liang et al. in their reports. We also conducted an extensive comparison of SAR patterns within and between active and inactive CDK groups. The specific methodology and approach we employed, along with our thorough analysis of a substantial dataset, contributed significantly to the novelty and importance of our research in the field.

### Virtual screening

Herein, we aim to demonstrate the practical applicability of the models developed in our study for the virtual screening of a large database. For this purpose, we randomly selected 2 million molecules from the PubChem database (see section “PubChem database”) which acted as the background database against which the active inhibitors were screened. The active inhibitors from each CDK group were added to the PubChem-R database, and the optimized classifiers were used to retrieve them by sorting the database.

**Figure 6:**
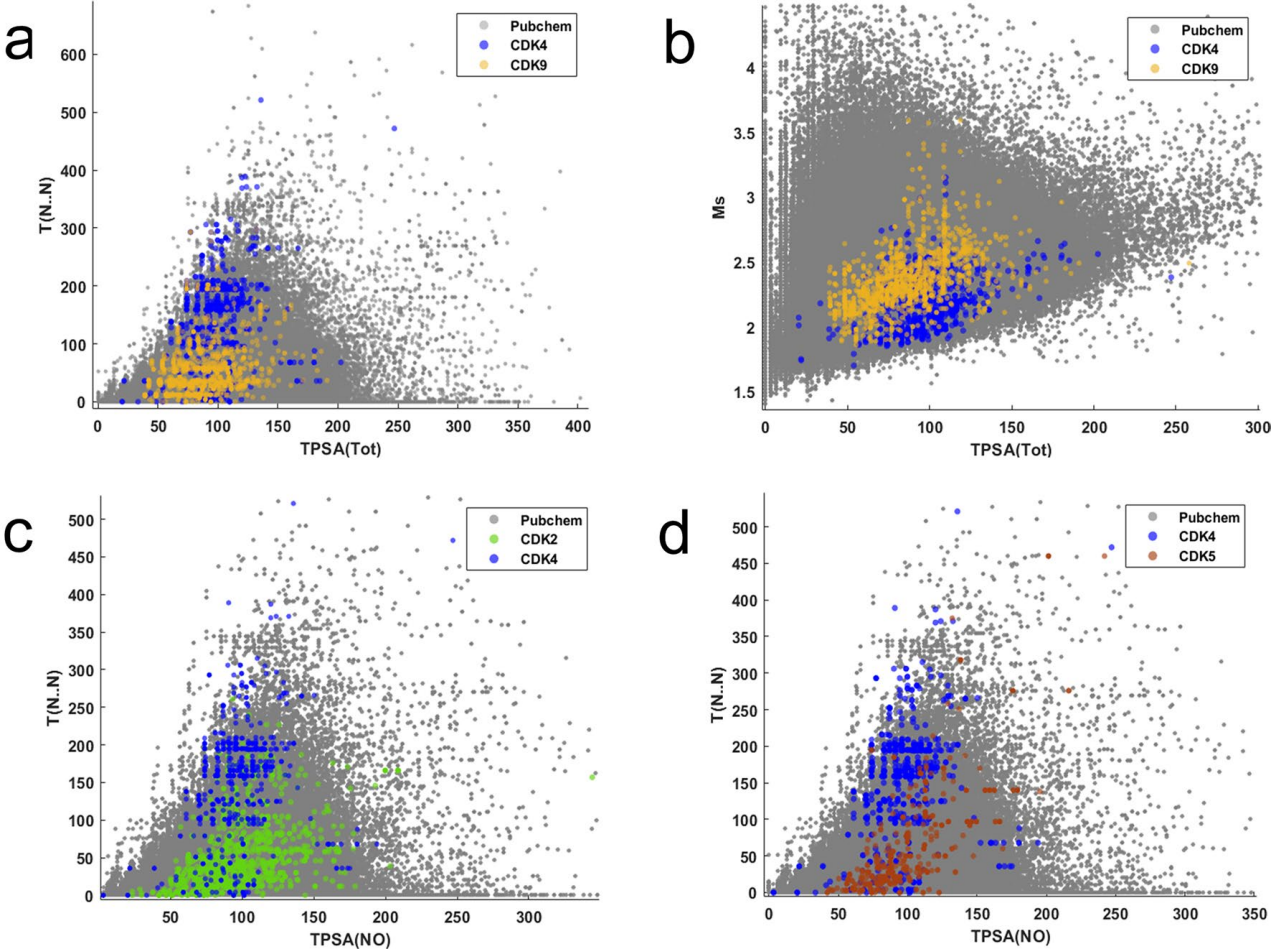
The distribution and coordinates of the PubChem-R and active CDK molecules within the two-dimensional subspaces created by one-by-one comparison of four VIP-selected molecular descriptors (**a**) TPSA_(Tot)_ versus T (N.. N), (**b**) TPSA_(Tot)_ versus Ms, (**c**) TPSA_(NO)_ versus T (N.. N), (**d**) TPSA_(NO)_ versus T (N.. N).

The ROC curves for screening the PubChem-R database using the CPANN and SKN active/inactive classifiers are shown in Fig. S19a,b, respectively. Additionally, Table S16 provides the calculated EF and AUC values for these models. This performance is attributable to the fact that the majority of active CDK4 inhibitors are concentrated within the top 1% of the sorted database. The ROC curves for screening of PubChem-R database using SKN and CPANN models developed for discriminating active molecules are presented in Fig. S19c,d. The calculated EF and AUC values for these models are summarized in Table S17. The AUC, EF_1%_, and EF_10%_ values for CDK4 and CDK9 groups are superior over other groups of active molecules. Figure 6 illustrates the distributions of the PubChem-R molecules and active CDK inhibitors in the chemical space made by distinct pairs of the VIP-selected molecular descriptors. Inspection of these figures reveals that the active CDK inhibitors are densely concentrated in specific regions of the selected chemical space of the PubChem database. As can be seen in Fig. 6, the active CDK4 molecules show higher values of topological distances between two nitrogen atoms (i.e. T(N..N)) in their structure compared to CDK2, CDK5, and CDK9 active molecules. Moreover, the values of the mean topological state (Ms) for CDK9 active molecules are significantly larger than those of CDK4 active molecules. These observations shed light on the distinct binding characteristics of CDK4 and CDK9, offering valuable information for drug design. The concept of “Chemography”, which involves exploring regions in chemical space occupied by molecules that bind to specific targets, was introduced in 2004 by Lipinski and Hopkins^57^. Figure 6 illustrates how this concept is achieved in this work where active CDK2, CDK4, CDK5 and CDK9 molecules are clustered in specific regions of the chemical space defined by VIP-selected descriptors. A significant difference exists between our results and the study reported by Qian et al.^16^ from the perspective of virtual screening. They assessed the model’s performance using Tox21^58^, SIDER^59^, and ClinTox^60^ databases, which consist of 7401, 1421, and 1377 compounds (10,199 in total)^16^, respectively. In contrast, our research involved virtual screening of an extensive dataset comprising 2,000,000 molecules randomly sourced from the PubChem database. To the best of our knowledge, this work is the first study to report the screening of a large database for retrieving active CDK inhibitors.

## Conclusion

This study lays the foundation for discovering the SARs within a diverse dataset of molecules targeting crucial subfamilies of CDKs, including CDK1, CDK2, CDK4, CDK5, and CDK9. Employing the VIP method, we identified important descriptors to develop binary active/inactive classifiers and multiclass classification models using the SKN and CPANN methods. The results indicated that molecular descriptors such as TPSA, nBnz, and Hy have substantial impacts on the activity and selectivity of molecules. For example, increasing the values of TPSA in a molecule probably leads to a significant improvement in the selectivity of CDK5 and CDK2. These findings contribute to a deeper understanding of the mechanisms underlying the inhibition of these targets. The accuracies of the developed SKN and CPANN models for the external test sets were more than 0.75 for all CDK targets. The resulting models demonstrated remarkable discriminatory power in distinguishing active CDK inhibitors from inactive ones, as well as effectively categorizing active molecules based on their therapeutic targets. Moreover, these models demonstrated robust performance for screening of a random subset of PubChem, comprising 2 million molecules. The SKN model achieved an average EF10% of 7.412 for activity-based classification, with AUC values ranging from 0.72 to 1.00. In therapeutic target-based classification, the SKN model reached an average EF10% of 7.033 and AUC range was between 0.84 and 0.96. The study's findings have enabled us to identify and describe distinctive subspaces for CDK inhibitors in chemical space. Our findings contribute valuable insights into the structural properties essential for explaining selective CDK inhibitors. The information obtained from this study is a stepping stone to help pharmacists and medicinal chemists to produce drugs with better efficacy and fewer side effects.

### Supplementary Information

Supplementary Information.

## Data Availability

The datasets used and/or analyzed during the current study available from the corresponding author on reasonable request.
