# Supplementary material for: Deriving general structure–activity/selectivity relationship patterns for different subfamilies of cyclin-dependent kinase inhibitors using machine learning methods
Source: Sci Rep. 2024 Jul 3;14:15315. doi: 10.1038/s41598-024-66173-z (PMC11222421; doi:10.1038/s41598-024-66173-z)
Supplement: Supplementary file 1 — Supplementary Information. [file 41598_2024_66173_MOESM1_ESM.pdf]

Supplementary Material

S.Kaveh [1], A. Mani-Varnosfaderani [1]*, M.S. Neiband [2]

1 Chemometrics and Cheminformatics Laboratory, Department of Analytical Chemistry, Tarbiat Modares University, Tehran, Iran

2 Department of Chemistry, Payame Noor University (PNU), P.O.Box 19395-4697, Tehran, Iran.

Supplementary Material

Figure S1. The flowcharts illustrate **(a)** data collection and preparation from the Binding DB. This includes 3D-optimization, descriptor calculation, pre-filtration, data cleaning and partitioning. The molecules were classified based on their activity state (left) and therapeutic targets (right) **(b)** the collection of molecules from the PubChem for virtual screening and model evaluation, **(c)** the procedure of preprocessing and splitting of the data set together with variable selection for the development of SKN and CPANN models for classification of CDK molecules in this work. The models were evaluated using 10-fold-CV, external test set and leverage approach.

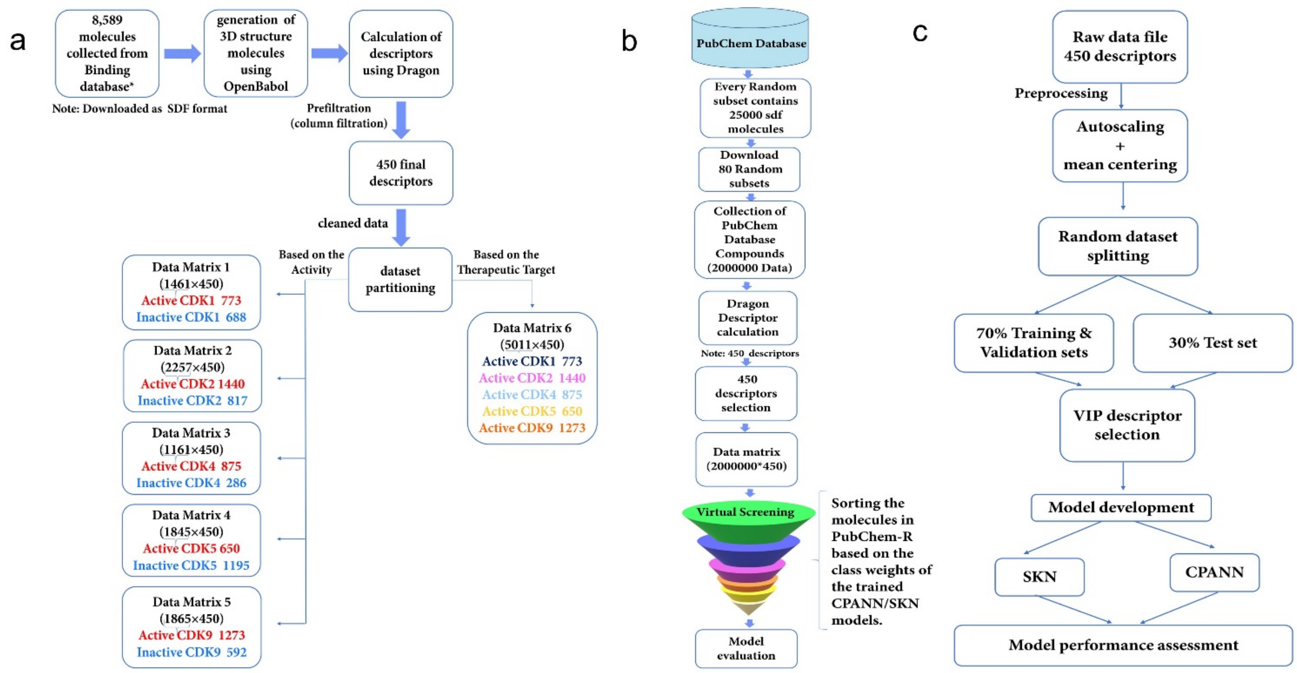

Supplementary Material

Figure S2. The scatter plot of MW *versus* TPSA $_{(Tot)}$ color-coded based on the values of MLOGP for two million randomly selected molecules from PubChem database.

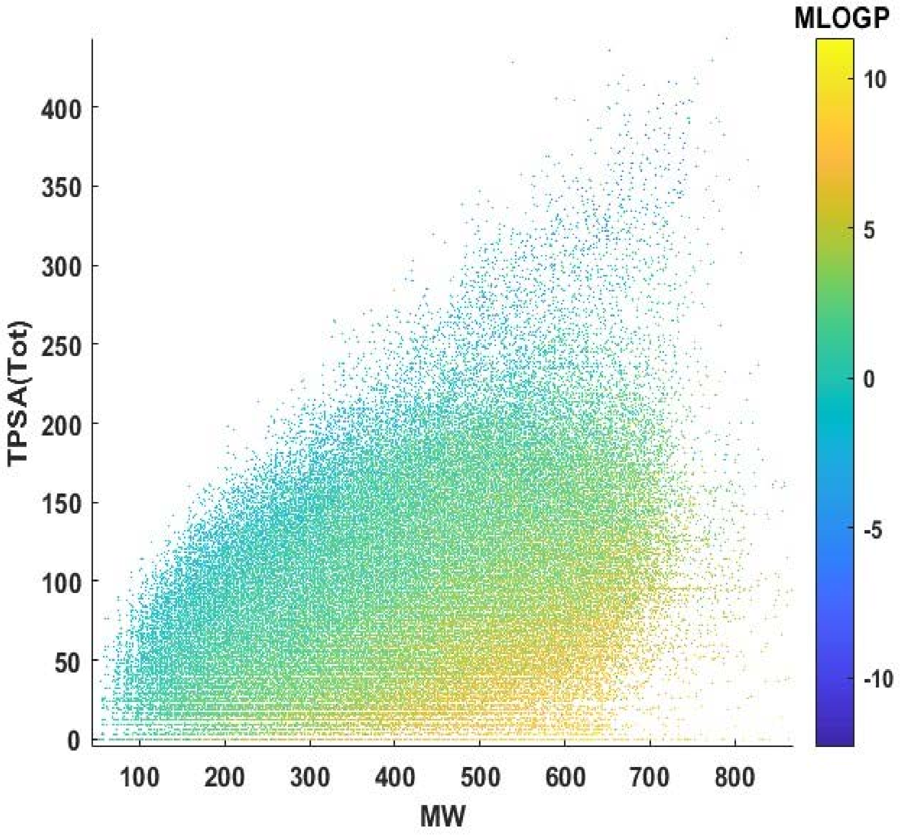

Supplementary Material

Figure S3: The CPANN maps for the classification of active and inactive molecules with map sizes and number of training epochs of **(a)** (26*26) and 350 for CDK1, **(b)** (28*28) and 100 for CDK2, **(c)** (28*28) and 50 for CDK4, **(d)** (22*22) and 100 for CDK5, **(e)** (26*26) and 150 for CDK9. Various map sizes and epochs were tested, and the optimal parameters were chosen. (Note: black hexagon refers to class 1: active inhibitors. White hexagon refers to class 2: inactive molecules.) **(f-j)** The ROC plots for the CPANN models for the classification of active and inactive molecules of five CDK groups :**(f)** CDK1, **(g)** CDK2, **(h)** CDK4, **(i)** CDK5, **(j)** CDK9.

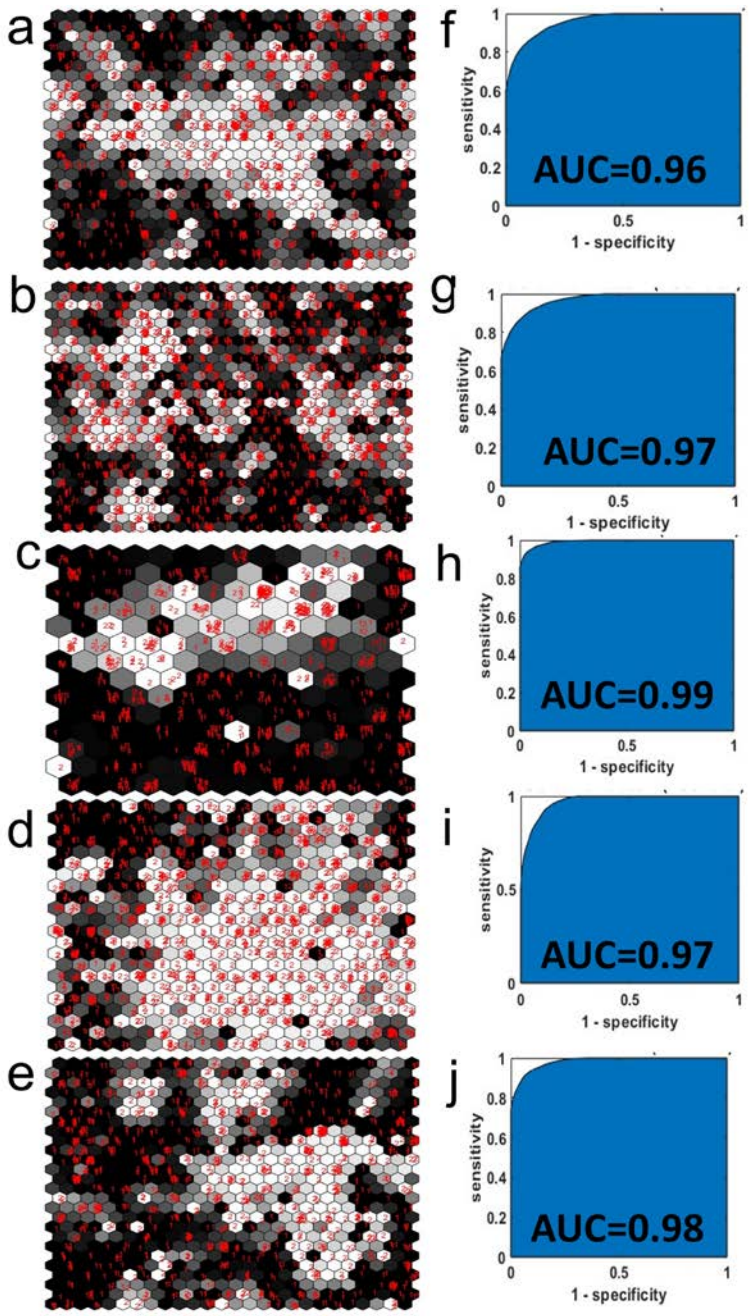

Supplementary Material

Figure S4: The SKN maps for the classification of active and inactive molecules with map sizes and number of training epochs of **(a)** (30*30) and 200 for CDK1, **(b)** (26*26) and 200 for CDK2, **(c)** (16*16) and 50 for CDK4, **(d)** (22*22) and 150 for CDK5, **(e)** (50*50) and 150 for CDK9. Various map sizes and epochs were tested, and the optimal parameters were chosen. (Note: Black hexagon refers to class 1: active inhibitors. White hexagon refers to class 2: inactive molecules. **(f-j)** The ROC plots for the SKN models for the classification of active and inactive five CDK: (**f:** CDK1, **g:** CDK2, **h:** CDK4, **i:** CDK5, **j:** CDK9).

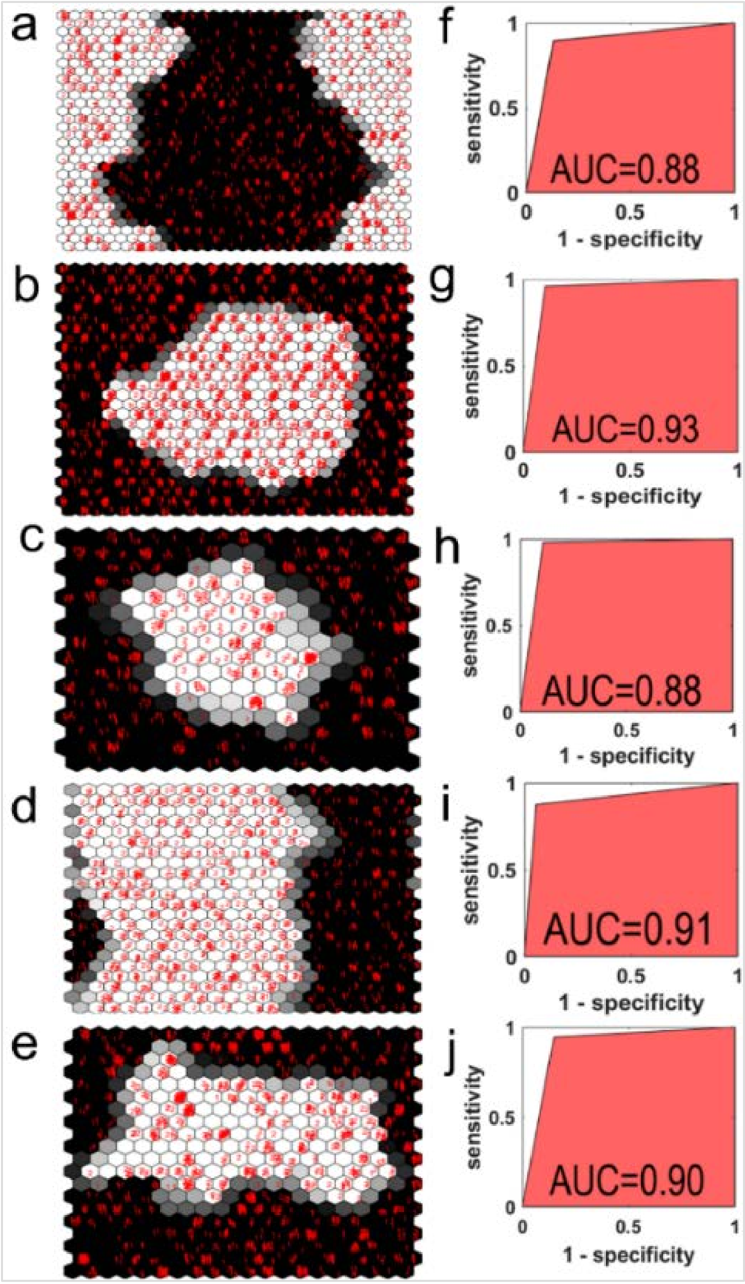

Supplementary Material

Figure S5: Projection of the active and inactive CDK molecules to the first three PCs made by the 20, 23, 17, 25, and 21 VIP selected descriptors for the active/inactive models for the binary classification of CDK molecules **(a)** CDK1 **(b)** CDK2 **(c)** CDK4 **(d)** CDK5 **(e)** CDK9. The three-dimensional tSNE space built using the VIP-selected molecular descriptors for classification of active and inactive **(f)** CDK1 **(g)** CDK2 **(h)** CDK4 **(i)** CDK5 **(j)** CDK9 molecules, respectively. Different perplexity values were examined for training the tSNE model and the best perplexity value was selected as 800, 1000, 1200, 1100, and 850 for **(f)** CDK1 **(g)** CDK2 **(h)** CDK4 **(i)** CDK5 **(j)** CDK9 molecules, respectively (Note: Red circles refer to active CDK molecules. Blue circles refer to inactive CDK molecules)

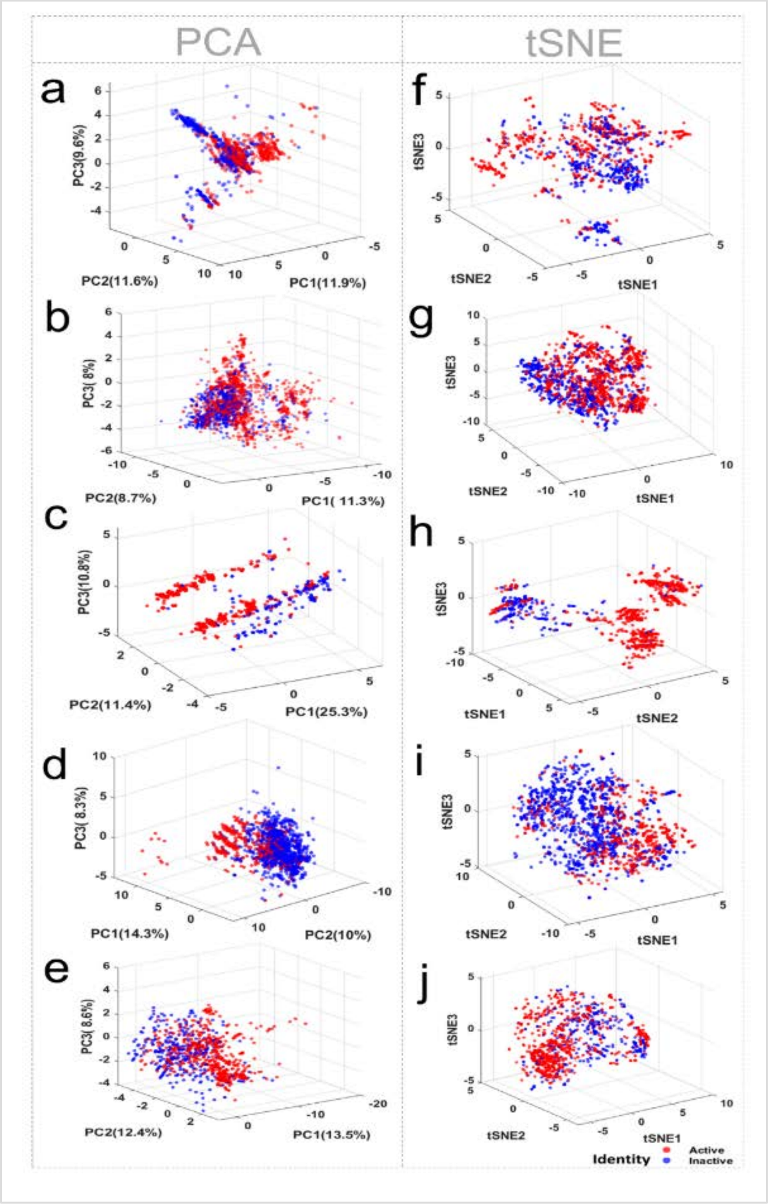

Supplementary Material

Figure S6: The density plot, box plot, and beeswarm plot **(a, b, c)** for nCconj **(d, e, f)** for S-110 **(g, h, i)** for nS **(j, k, l)** for T(N..S) molecular descriptor(s) for the active and inactive groups of CDK1 and CDK2 molecules

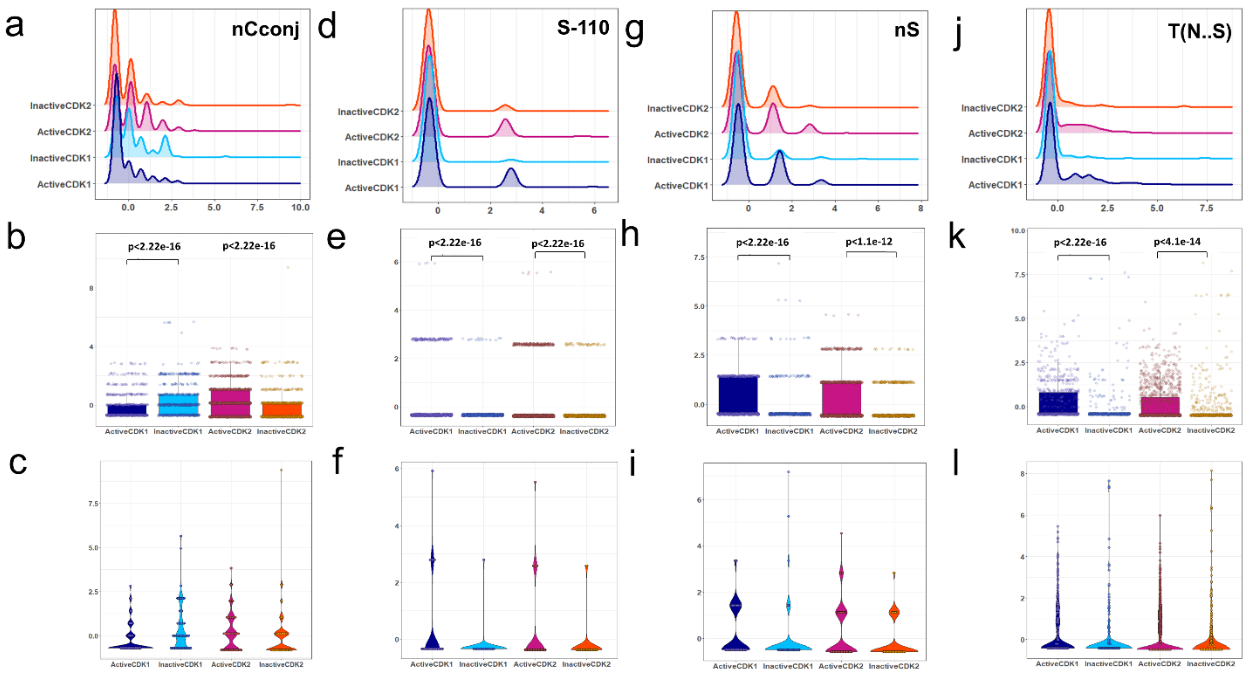

Supplementary Material

Figure S7. The density plot, box plot, and beeswarm plot **(a, b, c)** for N-072 molecular descriptor for the active and inactive groups of CDK1 and CDK5 molecules

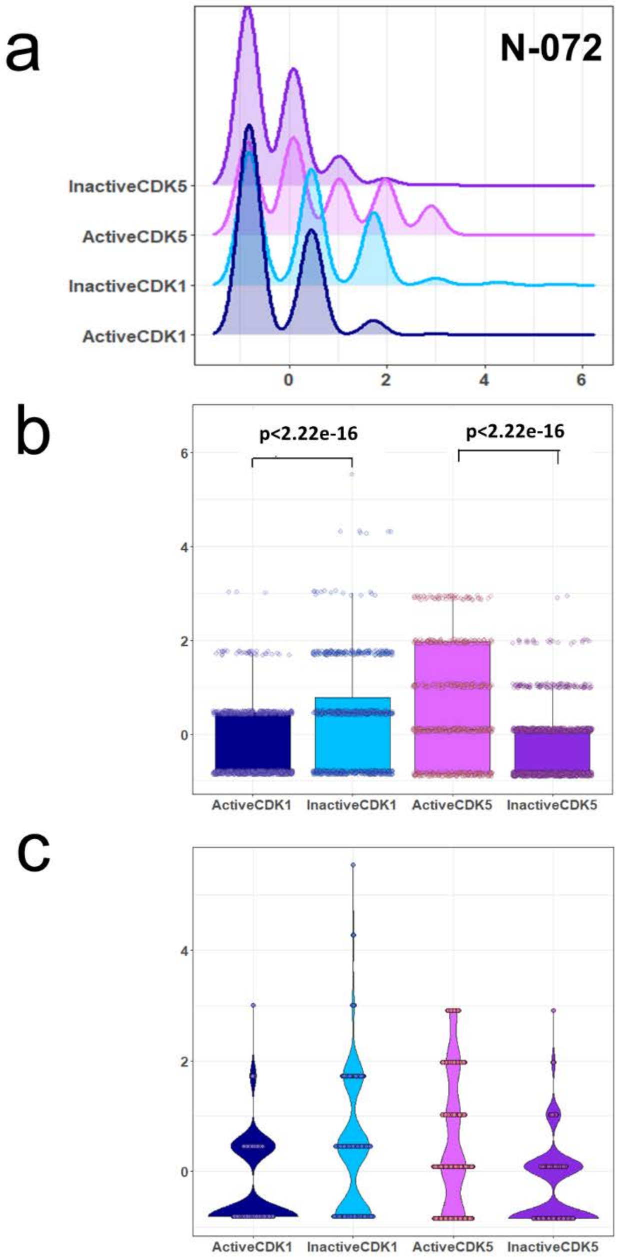

Supplementary Material

Figure S8. The density plot, box plot, and beeswarm plot **(a, b, c)** for T(N..F) **(d, e, f)** for nR=Ct **(g, h, i)** for H-051 **(j, k, l)** for nF molecular descriptor(s) for the active and inactive groups of CDK1 and CDK9 molecules

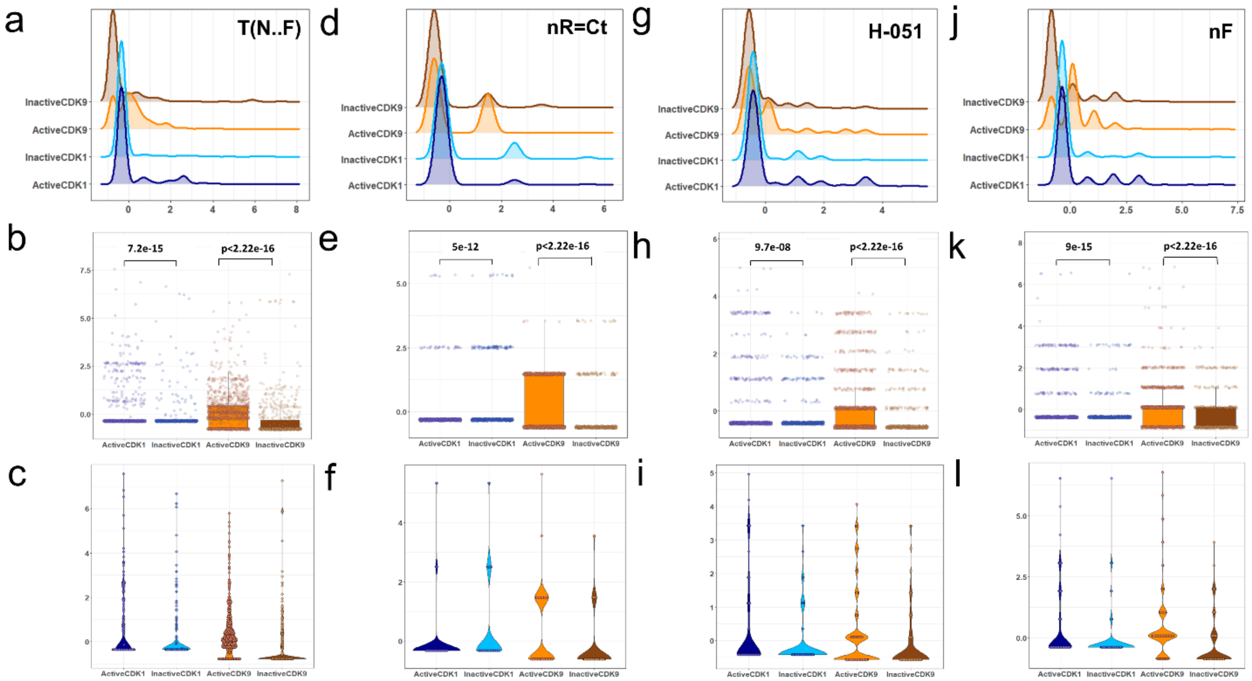

Supplementary Material

Figure S9. The density plot, box plot, and beeswarm plot **(a, b, c)** for O-060 (**d, e, f)** for nArOR molecular descriptor(s) for the active and inactive groups of CDK2 and CDK5 molecules

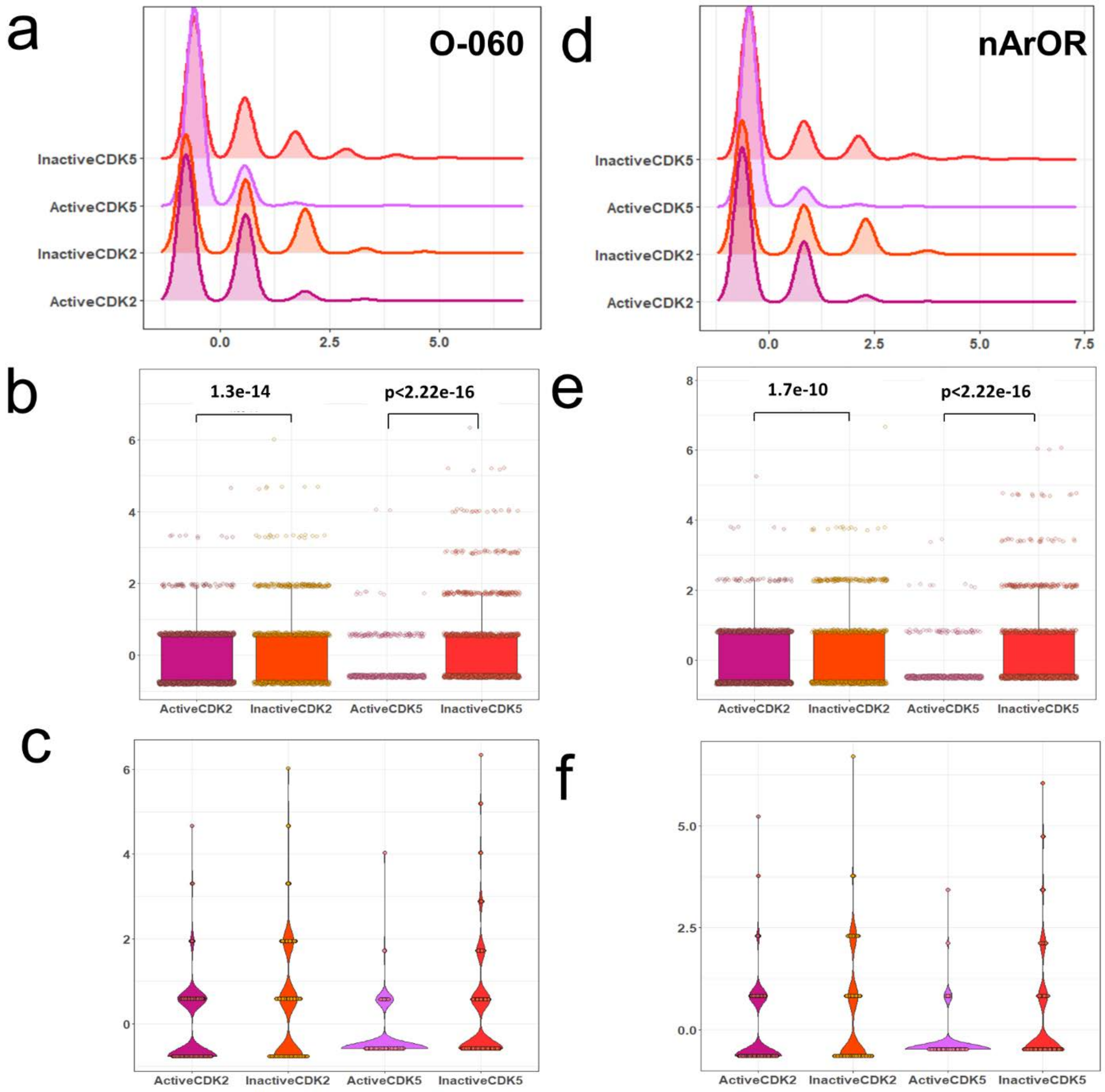

Supplementary Material

Figure S10. The density plot, box plot, and beeswarm plot **(a, b, c)** for C-029 (**d, e, f)** for F-084 **(g, h, i)** for T(O..F) **(j, k, l)** for C-034 molecular descriptor(s) for the active and inactive groups of CDK2 and CDK9 molecules

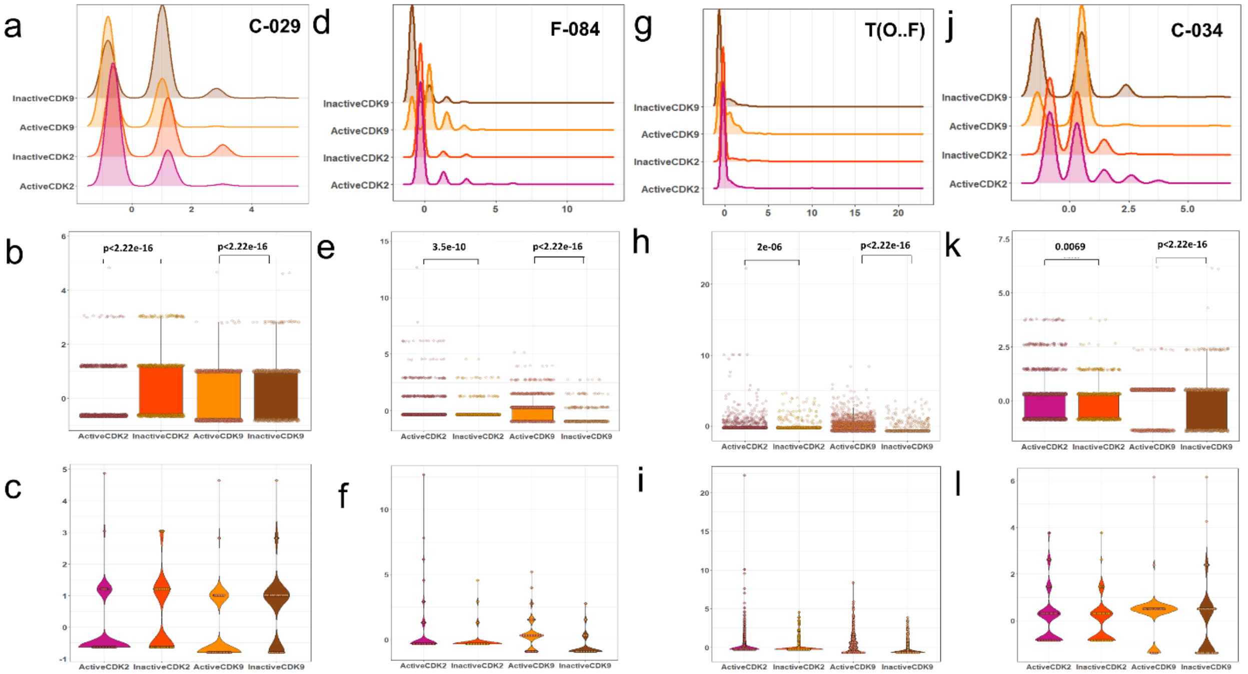

Supplementary Material

Figure S11. The density plot, box plot, and beeswarm plot **(a, b, c)** for C-027 **(d, e, f)** for nCbH **(g, h, i)** for nPyridines **(j, k, l)** for nPyrimidines **(m, n, o)** for C-032 molecular descriptor(s) for the active and inactive groups of CDK4 and CDK9 molecules

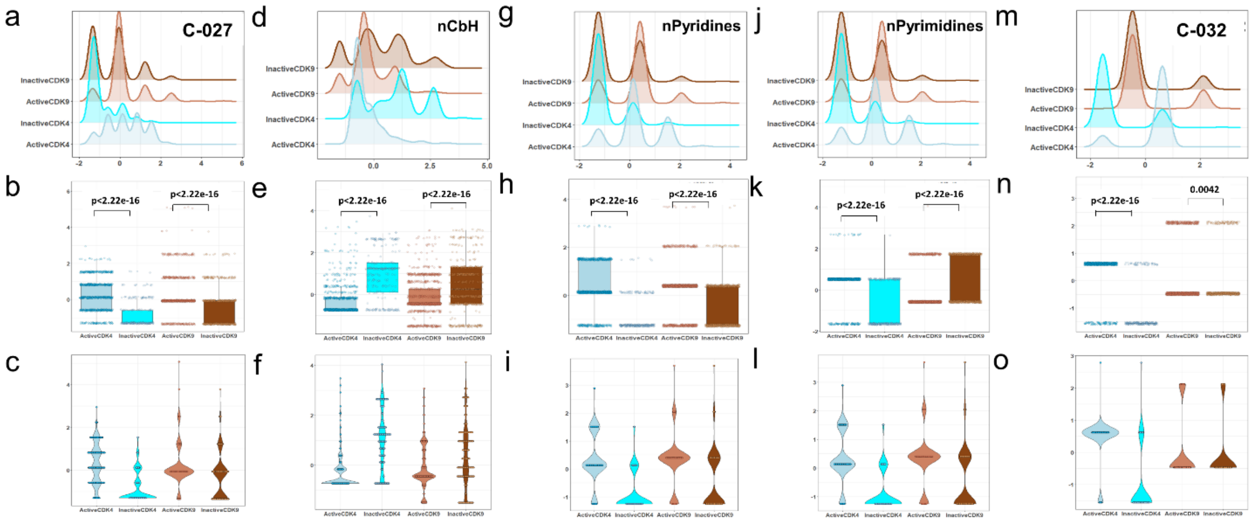

Supplementary Material

Figure S12. The density plot, box plot, and beeswarm plot **(a, b, c)** for nPyrroles molecular descriptor for the active and inactive groups of CDK5 and CDK9 molecules

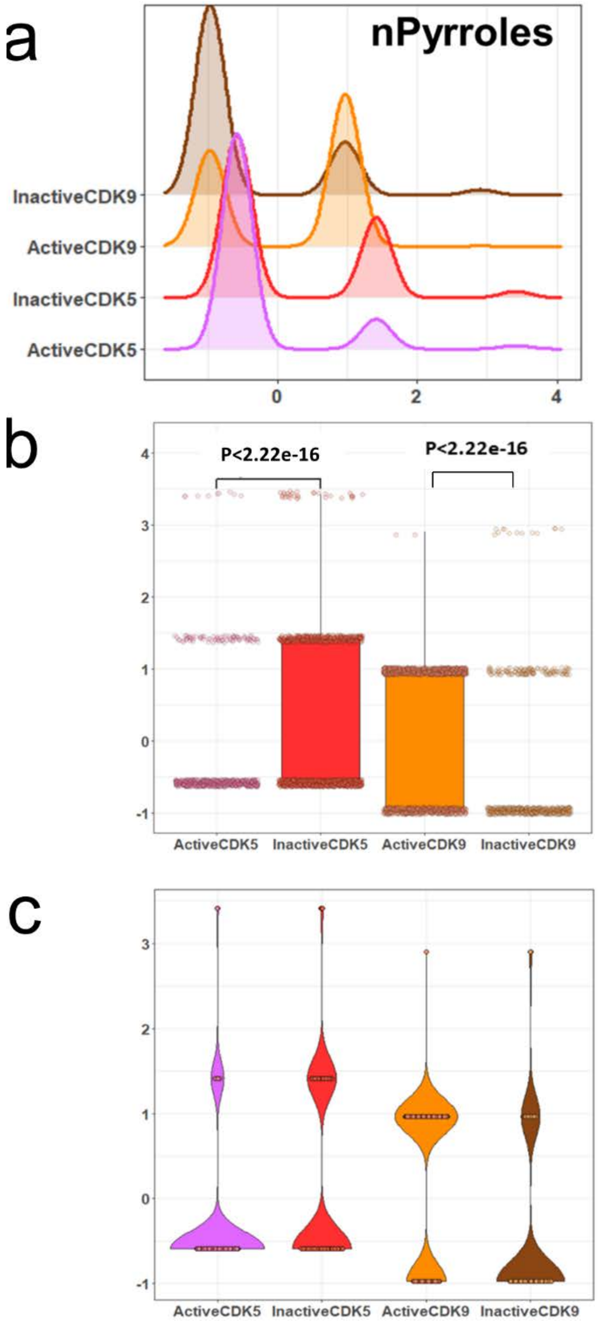

Supplementary Material

Figure S13. The PCA score plot for 77 unique molecular descriptors appeared in five active/inactive classifiers for CDK1, CDK2, CDK4, CDK5, and CDK9 categories **(a)** two-dimensional PCA (The first and second PCs explain 10.96% of the total variance of the data) **(b)** three-dimensional PCA (The first three PCs explain 15.61% of the total variance of the data.)

a

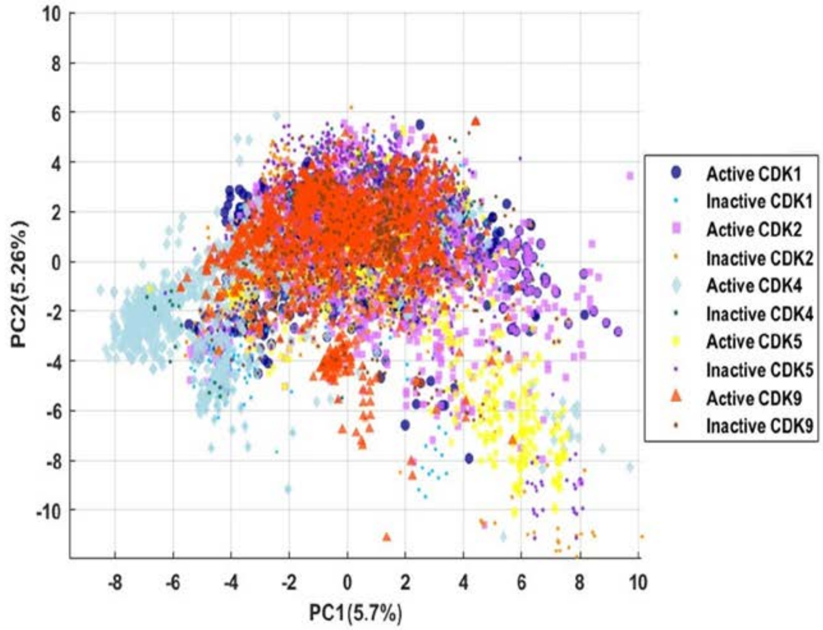

b

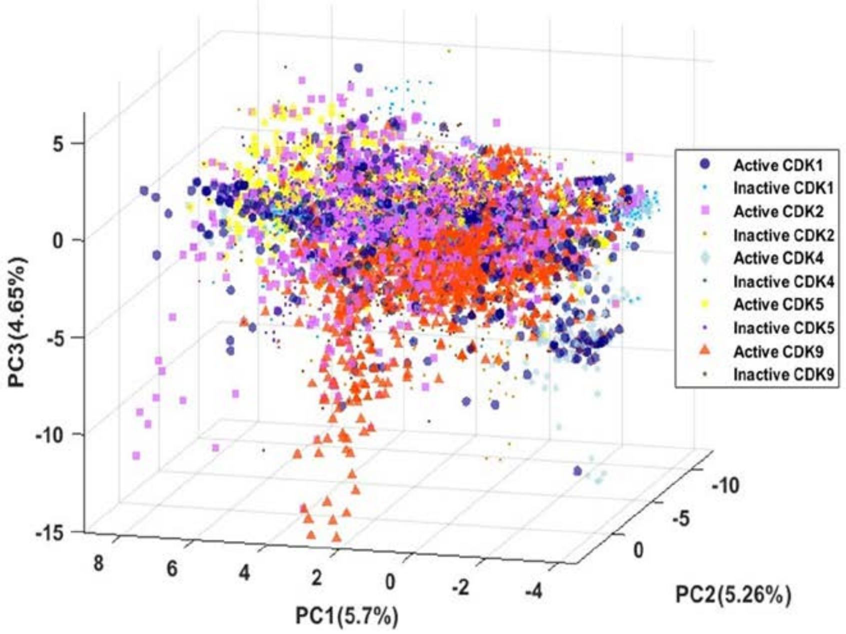

Supplementary Material

Figure S14. The tSNE plot for 77 unique molecular descriptors appeared in five active/inactive classifiers for CDK1, CDK2, CDK4, CDK5, and CDK9 categories **(a)** the two-dimensional tSNE map and the perplexity value of 2500 was used for this tSNE model **(b)** the three-dimensional tSNE map and the perplexity value of 2200 was used for this tSNE model

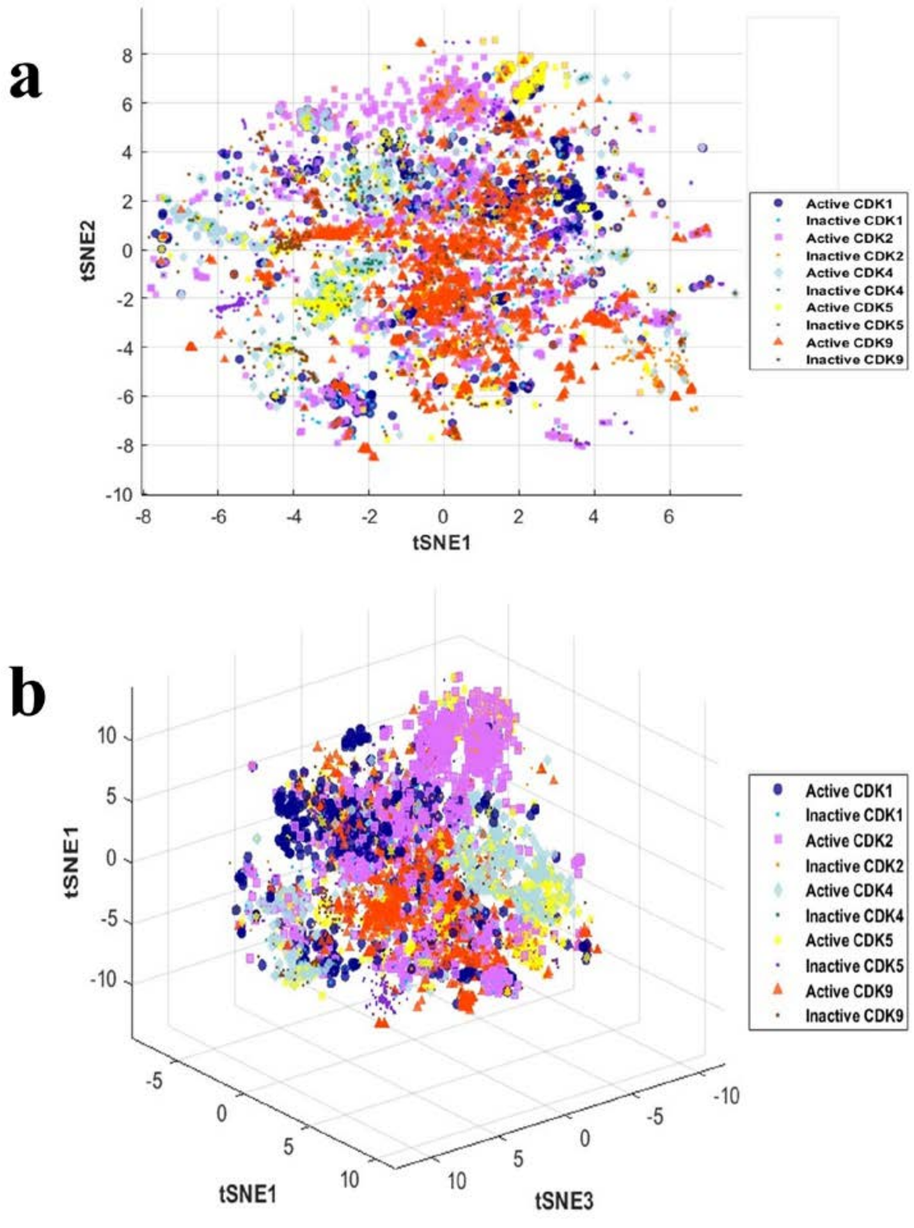

Supplementary Material

Figure S15. (a) The SKN map with the size of 25 × 25 neurons and 50 training epochs for multi-class classification of active CDK inhibitors. Slate blue, light coral, pale green, orchid, and deep pink hexagons refer to class 1, 2, 3, 4 and 5 as active CDK1, active CDK2, active CDK4, active CDK5 and active CDK9 inhibitors (Note: the multi-class SKN model was developed to categorize active CDK molecules based on their therapeutic targets.) **(b)** The ROC plots of the SKN model for the multi class classification of the active inhibitors for five groups of CDK inhibitors: CDK1(dark blue), CDK2 (medium violet red), active CDK4 (blue), active CDK5 (pink), active CDK9 (orange).

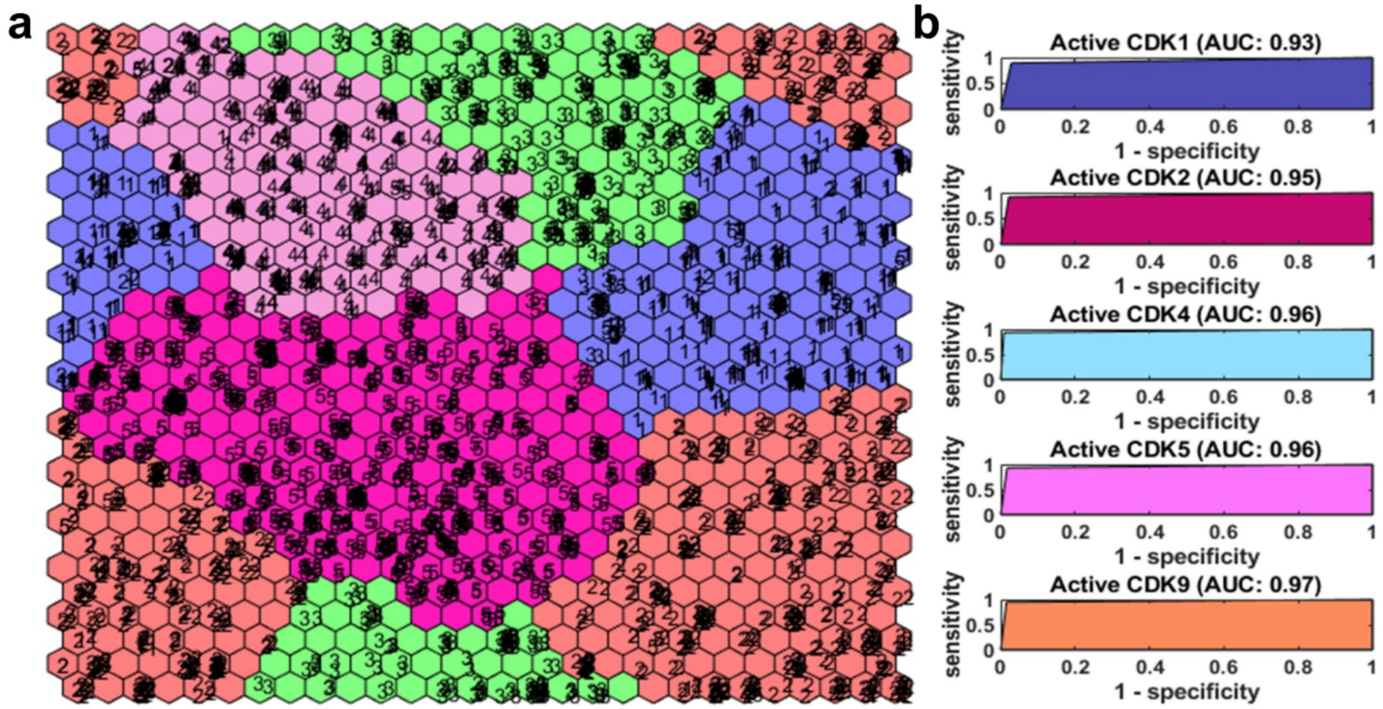

Figure S16. The projection of the active CDK inhibitors to the first three principal components made by the 31 VIP-selected descriptors for classification of inhibitors based on their therapeutic targets (The first three PCs explain 24.1% of the total variance of the data)

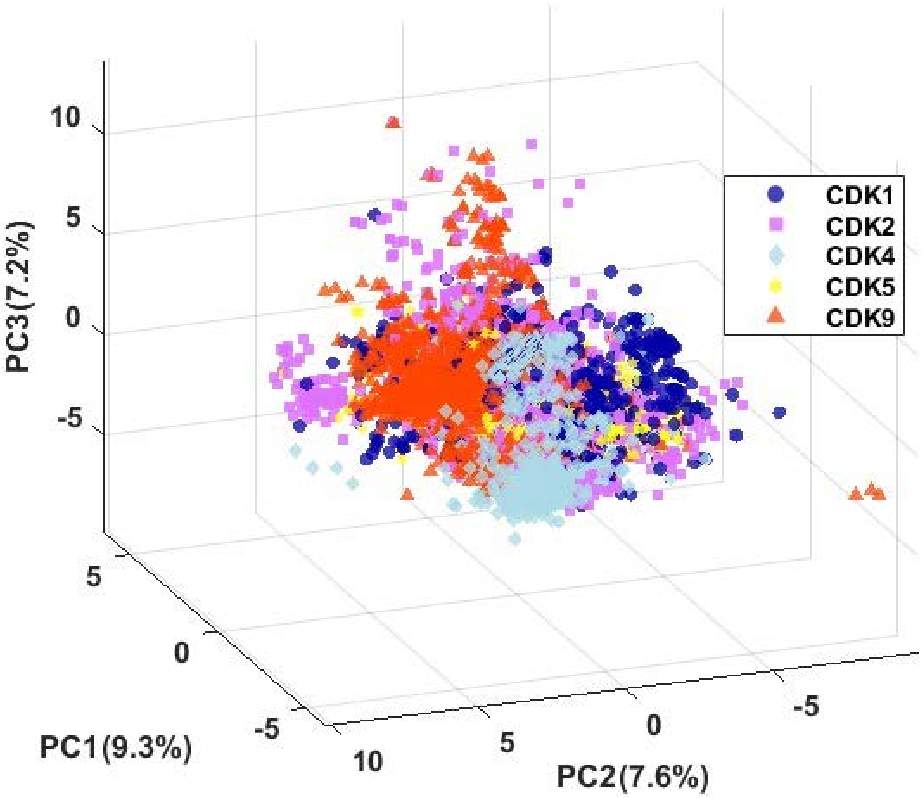

Figure S17. The three-dimensional tSNE space of the set of 31 VIP selected molecular descriptors for active CDK1, CDK2, CDK4, CDK5, and CDK9 inhibitors. For the tSNE model, the perplexity value of 850 was used. (Note: different perplexity values were examined for training the tSNE model and the best perplexity was selected)

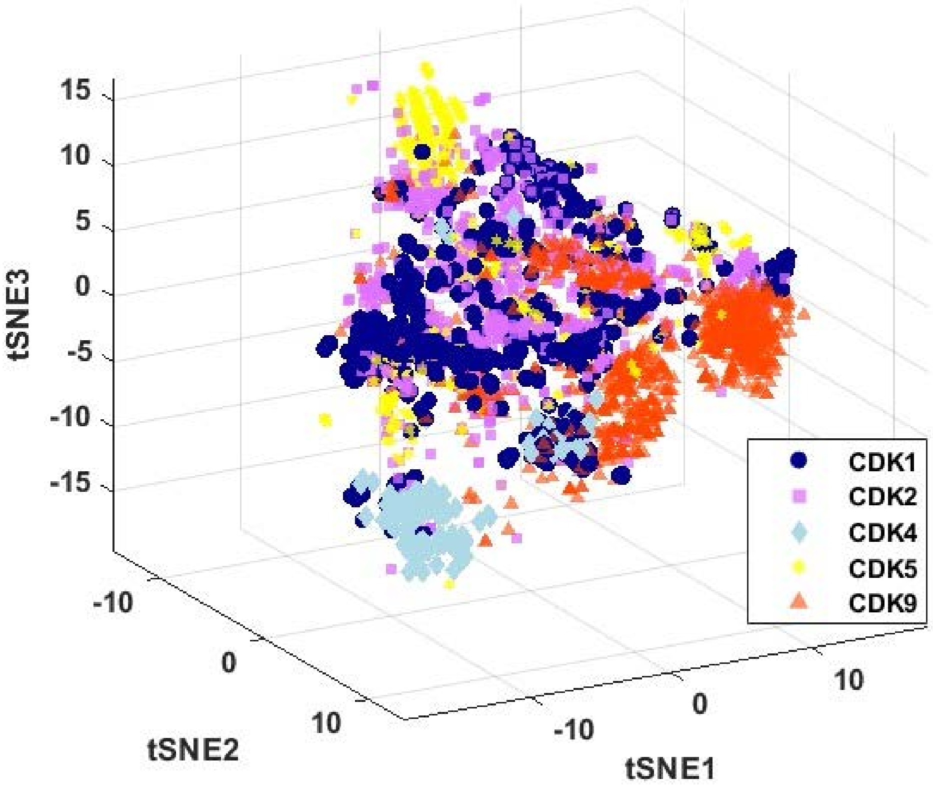

Supplementary Material

Figure S18. The density plot, box plot, and beeswarm plot for the VIP-selected molecular descriptors for the multiclassification of active CDK inhibitors. **(Sub1, sub2, sub3)** for nN **(sub4, sub5, sub6)** for T(N..N) **(sub7, sub8, sub9)** for nHDon **(sub10, sub11, sub12)** for nS **(sub13, sub14, sub15)** for nPyridines **(sub16, sub17, sub18)** for nPyrimidines **(sub19, sub20, sub21)** for nCb- **(sub22, sub23, sub24)** for nBnz **(sub25, sub26, sub27)** for nPyrroles **(sub28, sub29, sub30)** for nCbH **(sub31, sub32, sub33)** for S-107 **(sub34, sub35, sub36)** for H-049 **(sub37, sub38, sub39)** for T(N..S) **(sub40, sub41, sub42)** for T(O..F) **(sub43, sub44, sub45)** for T(O..S) **(sub46, sub47, sub48)** for nArNHR **(sub49, sub50, sub51)** for nPyrazoles **(sub52, sub53, sub54)** for C-029 **(sub55, sub56, sub57)** for nArX **(sub58, sub59, sub60)** for N-070 **(sub61, sub62, sub63)** for nR=Ct **(sub64, sub65, sub66)** for nCrt **(sub67, sub68, sub69)** for TPSA$_{(NO)}$ **(sub70, sub71, sub72)** for nX **(sub73, sub74, sub75)** for nF **(sub76, sub77, sub78)** for nRNHR **(sub79, sub80, sub81)** for nCconj **(sub82, sub83, sub84)** for T(N..O).

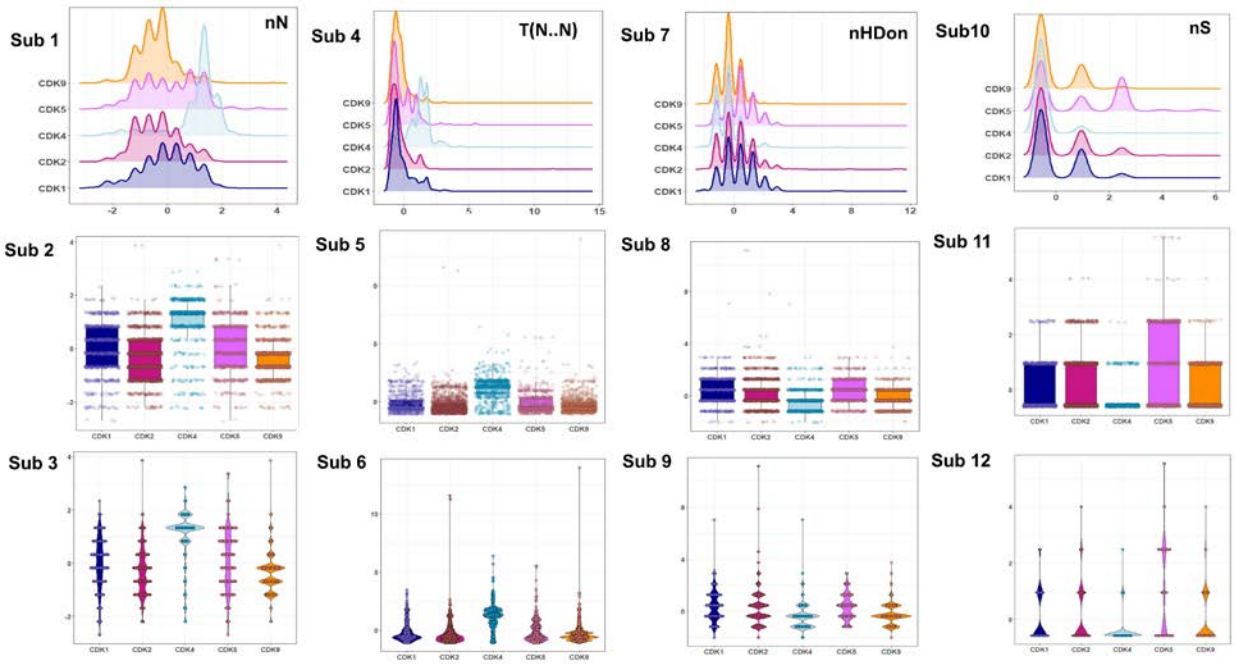

Supplementary Material

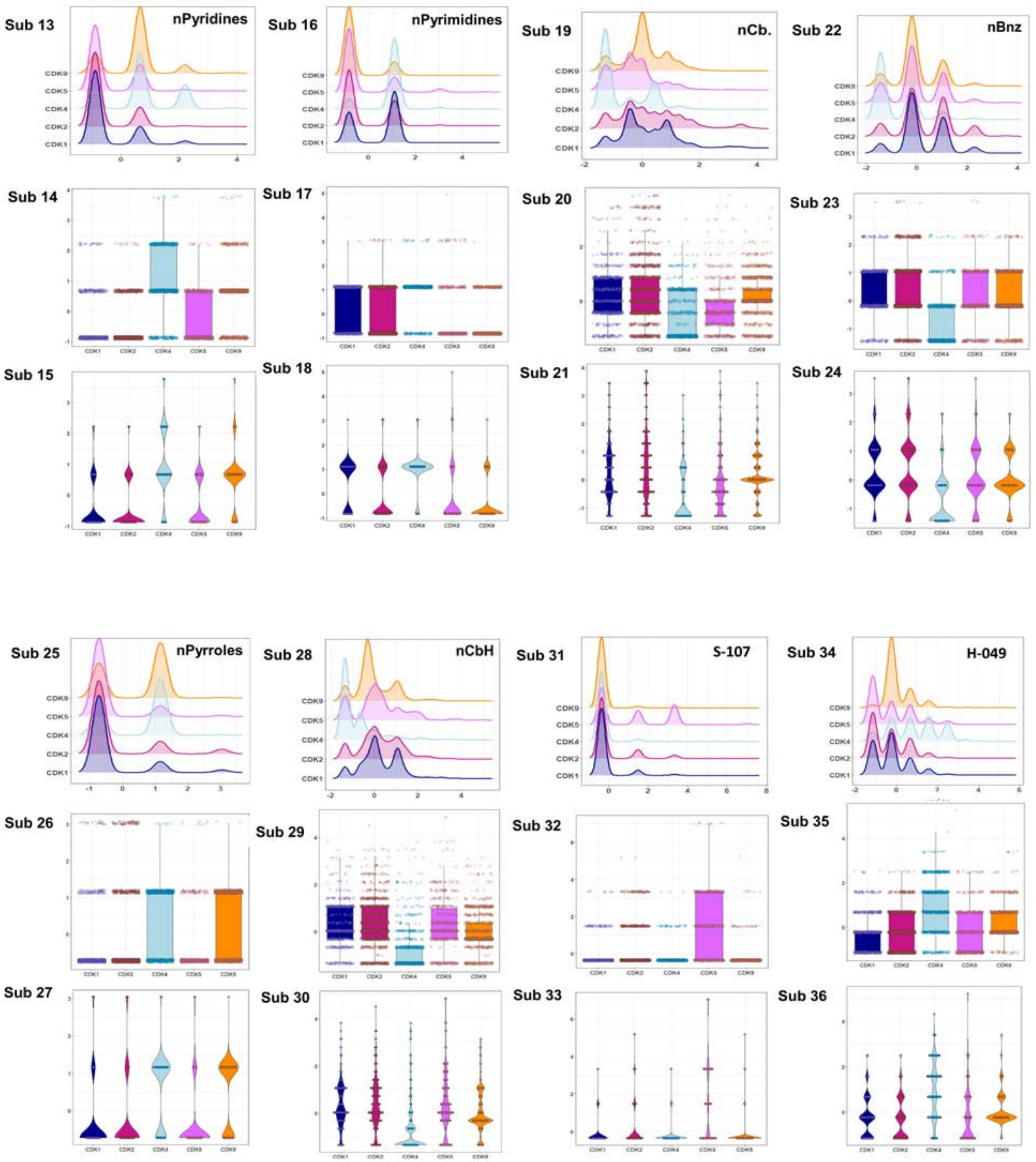

Supplementary Material

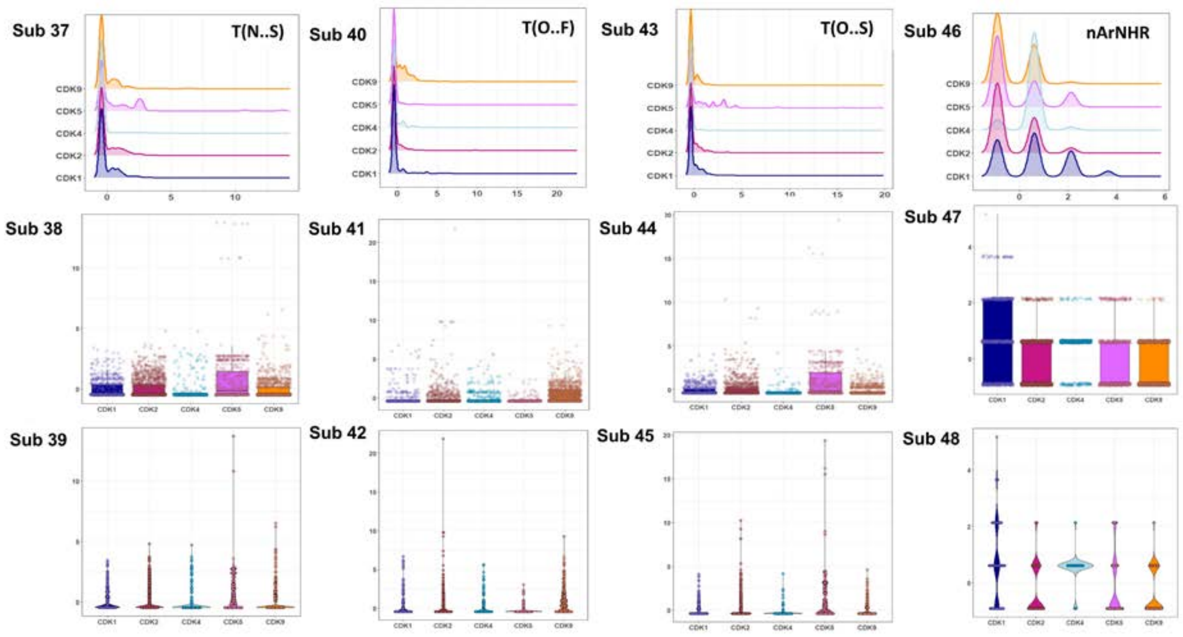

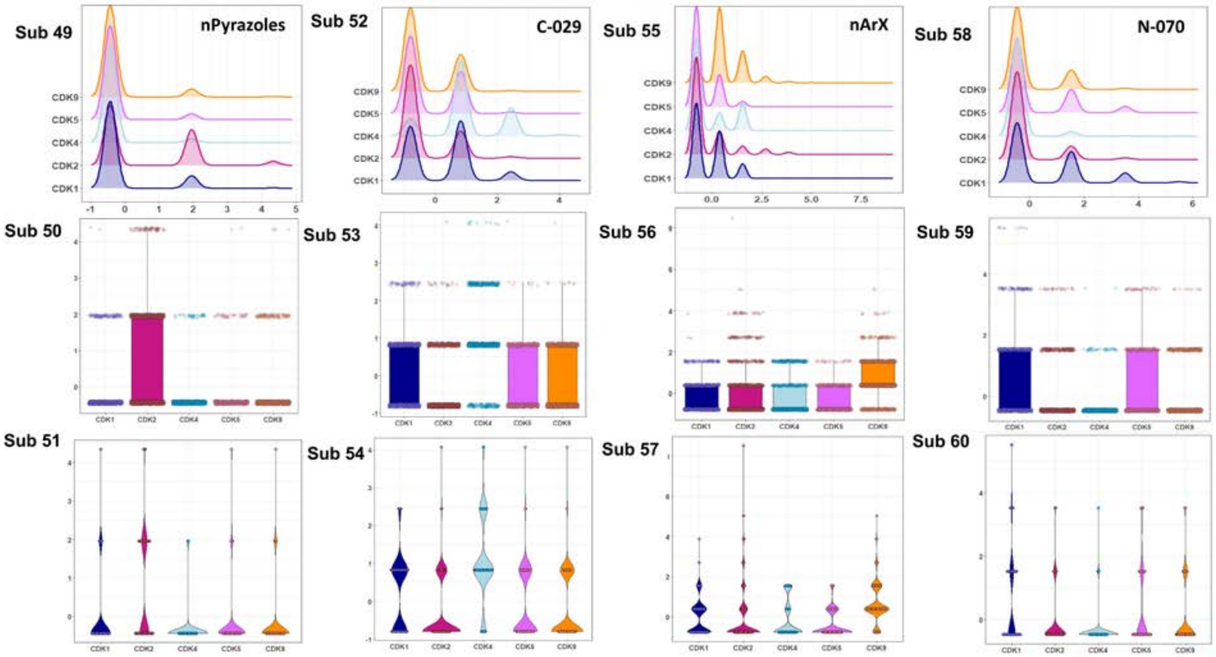

Supplementary Material

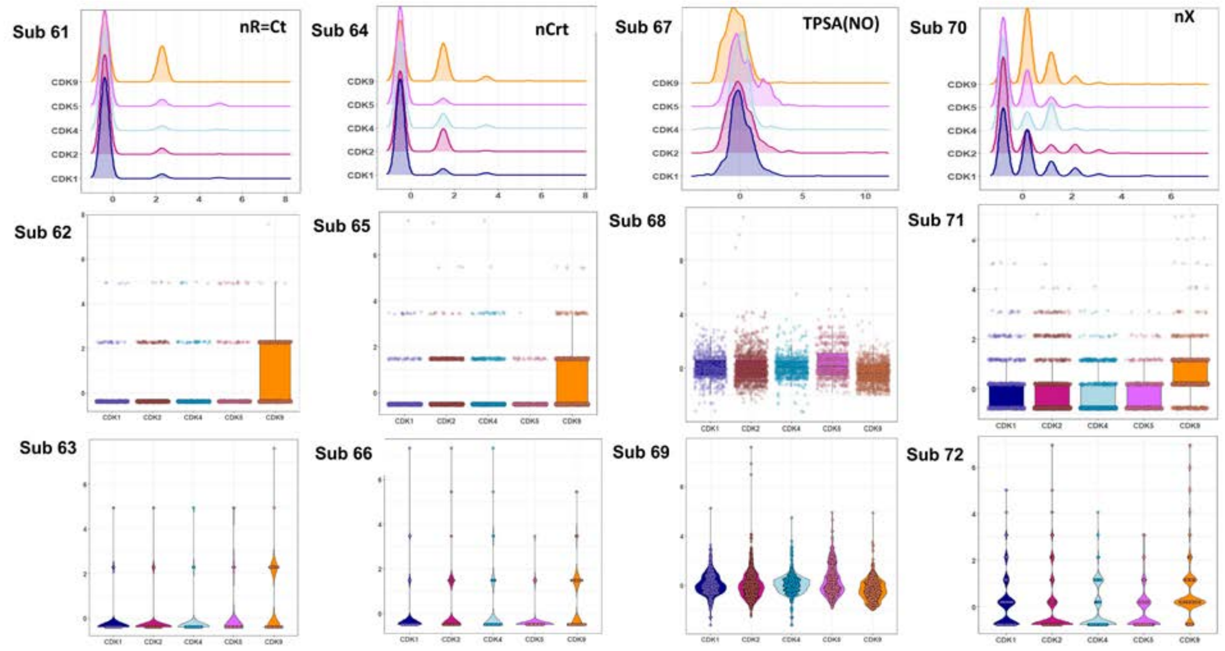

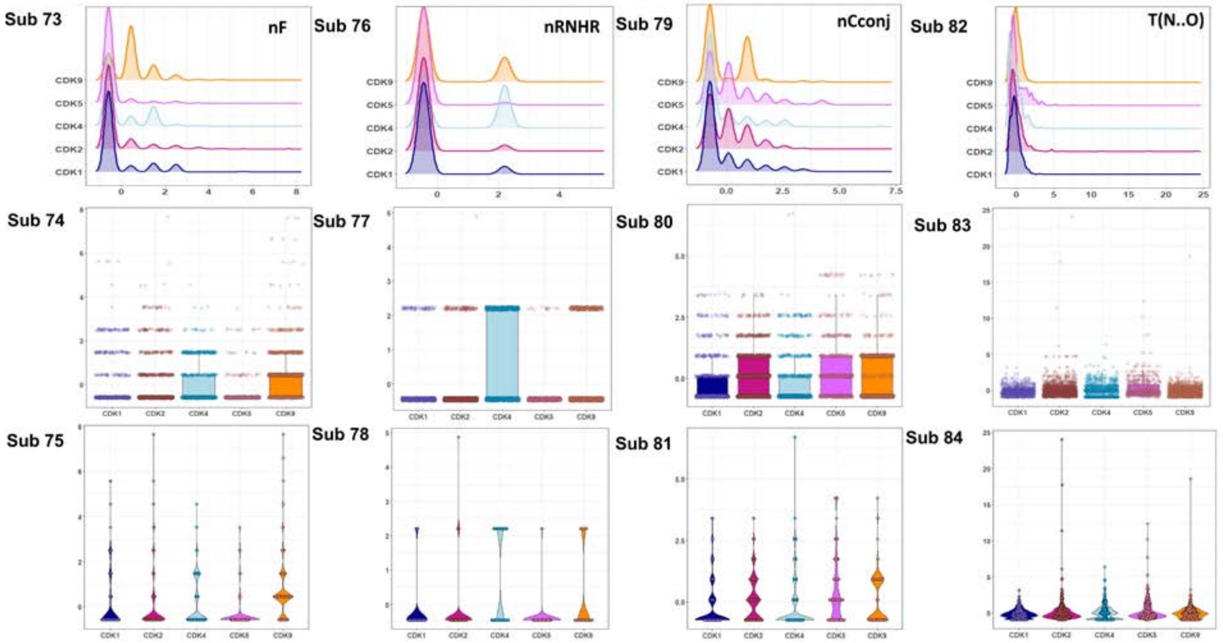

Supplementary Material

Figure S19. The receiver operating characteristic curves for screening of PubChem-R database using **(a)** CPANN and **(b)** SKN active/inactive classifiers. The receiver operating characteristic curves for screening of PubChem-R database using **(c)** CPANN and **(d)** SKN multi-class classifiers. (Note: the multi-class classifiers were developed to categorize active CDK molecules based on their therapeutic targets.)

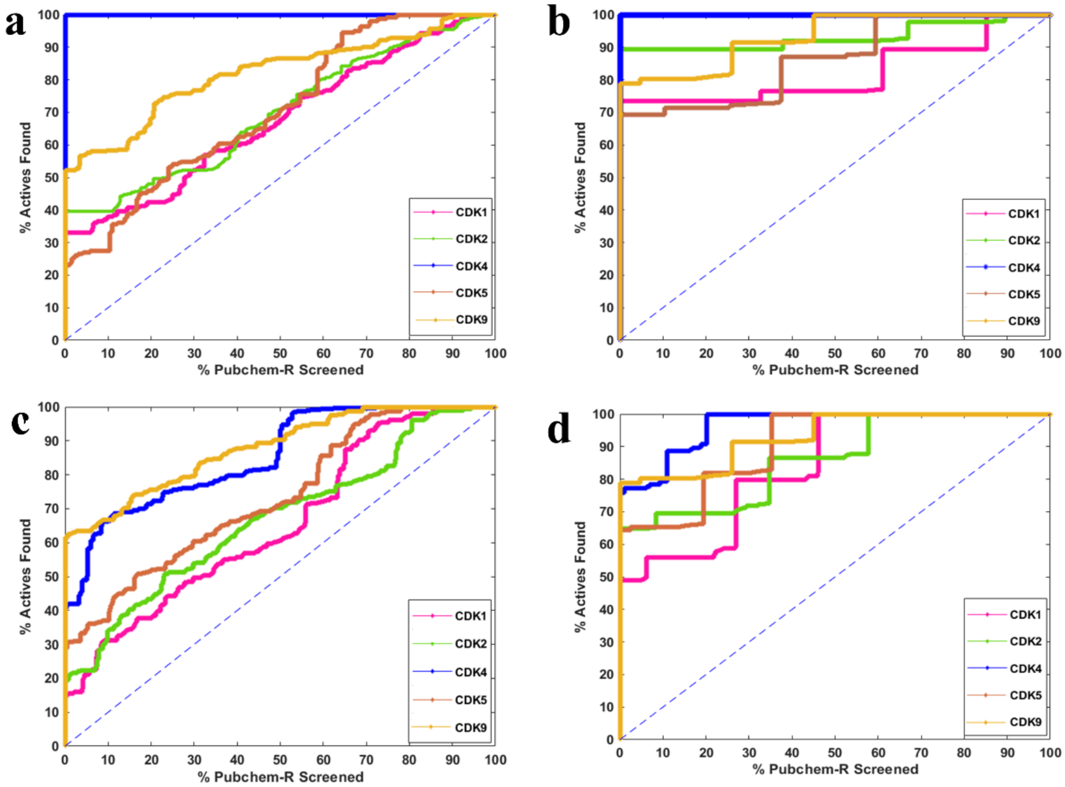

Supplementary Material

Table S1. The detailed enumeration of the collected CDK molecules in this work. The molecules in active and inactive groups were used for the development of the classification models.

Target	Active		Inactive		Total number of molecules	References
	Range of activity	Number of compounds	Range of activity	Number of compounds		
CDK1	IC_{50} =0-1500 nM	773	IC_{50}≥10000 nM	688	1461	(1, 2)
CDK2	IC_{50} = 0-1000 nM	1440	IC50≥10000 nM	817	2257	(3, 4)
CDK4	IC_{50} = 0-80 nM	875	IC_{50}≥800 nM	286	1161	(5, 6)
CDK5	IC_{50} =0-70 nM	650	IC_{50}≥1400 nM	1195	1845	(7, 8)
CDK9	IC_{50} =0-2000 nM	1273	IC_{50}≥10000 nM	592	1865	(9, 10)

Supplementary Material

Table S2. Definition, type, Mann-Whitney test *p*-value, mean, and standard deviation of 20 VIP-selected molecular descriptors for active/inactive classification of CDK1 molecules.

Selected molecular descriptors	Definition	Type	Mann–Whitney test *p*-value	Mean value for active CDK1 Molecules	Mean value for inactive CDK1 Molecules	Std value for active CDK1 Molecules	std value for inactive CDK1 Molecules
C-028	R--CR--X	Atom-centered fragments	**< 2.2e-16**	**0.5462012**	**0.3357558**	**0.7955921**	**0.5414956**
C-040	R-C(=X)-X / R-C#X / X=C=X	Atom-centered fragments	**< 2.2e-16**	**0.7597536**	**1.059593**	**0.8706294**	**0.950413**
nRCONHR	number of secondary amides (aliphatic)	Functional group counts	**3.026e-14**	**0.1047228**	**0.03924419**	**0.3107408**	**0.1943167**
nArCONR2	number of tertiary amides (aromatic)	Functional group counts	**< 2.2e-16**	**0.2299795**	**0.4200581**	**0.5199624**	**0.6603814**
C-043	X--CR..X	Atom-centered fragments	**5.102e-15**	**0.5017112**	**0.6148256**	**0.539689**	**0.5326717**
H-051	H attached to alpha-C	Atom-centered fragments	**9.656e-08**	**0.5482546**	**0.3125**	**1.299064**	**0.8563595**
S-110	R-SO2-R	Atom-centered fragments	**< 2.2e-16**	**0.1081451**	**0.02180233**	**0.319367**	**0.1461439**
nCconj	number of non-aromatic conjugated	Functional group counts	**< 2.2e-16**	**0.9890486**	**1.34593**	**1.419489**	**1.566418**
N-072	RCO-N< / >N-X=X	Atom-centered fragments	**< 2.2e-16**	**0.6406571**	**0.8909884**	**0.7852863**	**0.8905325**
T(O..S)	sum of topological distances between O..S	2D Atom Pairs	**< 2.2e-16**	**3.332649**	**1.146802**	**8.468731**	**4.958367**
T(N..S)	sum of topological distances between N..S	2D Atom Pairs	**< 2.2e-16**	**6.041752**	**2.901163**	**14.63933**	**12.64262**
nR=Ct	number of aliphatic tertiary C(sp^2)	Functional group counts	**< 2.2e-16**	**0.1129363**	**0.1758721**	**0.3534231**	**0.4243661**
C-017	=CR2	Atom-centered fragments	**5.196e-12**	**0.1229363**	**0.1854731**	**0.5524261**	**0.5241562**
T(N..F)	sum of topological distances between N..F	2D Atom Pairs	**7.22e-15**	**14.38467**	**6.755814**	**40.11516**	**30.32605**
C-002	CH2R2	Atom-centered fragments	**2.125e-06**	**1.978782**	**2.508721**	**2.562418**	**3.009434**
nS	number of Sulfur atoms	Constitutional indices	**< 2.2e-16**	**0.2553046**	**0.130814**	**0.5206499**	**0.4387138**
Hy	hydrophilic factor	Molecular properties	**< 2.2e-16**	**0.7258768**	**0.4818212**	**0.9136616**	**0.7797807**
C-005	CH3X	Atom-centered fragments	**< 2.2e-16**	**0.7679671**	**1.024709**	**0.9357221**	**1.002602**
nF	number of Fluorine atoms	Constitutional indices	**8.989e-15**	**0.3292266**	**0.1511628**	**0.8682537**	**0.6011622**
Qneg	total negative charge	Charge descriptors	**2.207e-09**	**-7.977299**	**-2.596449**	**73.56236**	**0.5823589**

Supplementary Material

Table S3. Definition, type, Mann-Whitney test *p*-value, mean, and standard deviation of 23 VIP-selected molecular descriptors for active/inactive classification of CDK2 molecules.

Selected molecular descriptors	Definition	Type	Mann–Whitney test *p*-value	Mean value for active CDK2 Molecules	Mean value for inactive CDK2 Molecules	Std value for active CDK2 Molecules	std value for inactive CDK2 Molecules
C-029	R--CX--X	Atom-centered fragments	< 2.2e-16	0.2479167	0.5128519	0.4630126	0.6302379
O-058	= O	Atom-centered fragments	< 2.2e-16	1.515278	1.133415	1.136196	1.173888
nDB	number of double bonds	Constitutional indices	< 2.2e-16	1.817361	1.367197	1.343909	1.433083
qnmax	maximum negative charge	Charge descriptors	0.002763	-0.3994194	-0.4152509	0.08627927	0.08487773
F-084	F attached to C1(sp^2)	Atom-centered fragments	3.545e-10	0.2604167	0.1003672	0.7050615	0.3894596
C-034	R--CR..X	Atom-centered fragments	0.00687	0.7923611	0.6315789	0.9383039	0.71752
H-046	H attached to C0(sp^3) no X attached to next C	Atom-centered fragments	0.1292	2.725	3.320685	3.791939	4.741605
C-035	R--CX..X	Atom-centered fragments	1.394e-08	0.1715278	0.08323133	0.3862046	0.2764008
nS	number of Sulfur atoms	Constitutional indices	1.079e-12	0.4104167	0.2141983	0.6416426	0.4503747
Ui	unsaturation index	Molecular properties	0.0187	4.254055	4.188356	0.3519936	0.474765
TPSA(Tot)	topological polar surface area using N, O,S,P polar contributions	Molecular properties	< 2.2e-16	102.618	94.52865	34.11922	47.86116
O-060	Al-O-Ar / Ar-O-Ar / R..O..R / R-O-C=X	Atom-centered fragments	1.288e-14	0.4541667	0.7625459	0.6097283	0.8841842
S-110	R-SO2-R	Atom-centered fragments	1.761e-13	0.1659722	0.05630355	0.3814065	0.2306482
Qmean	mean absolute charge (charge polarization)	Charge descriptors	4.504e-06	0.1000285	0.09810894	0.01574854	0.02154565
nRCONHR	number of secondary amides (aliphatic)	Functional group counts	6.488e-14	0.33125	0.2203182	0.5721069	0.6246006
nSO2N	number of sulfonamides (thio-/dithio)	Functional group counts	3.341e-12	0.1444444	0.04773562	0.3575412	0.2133369
T(N..S)	sum of topological distances between N..S	2D Atom Pairs	4.057e-14	10.21736	5.555692	17.67746	18.13889
nR09	number of 9-membered rings	Ring descriptors	< 2.2e-16	1.007639	0.6352509	17.67746	0.7270212
C-039	Ar-C(=X)-R	Atom-centered fragments	< 2.2e-16	0.1986111	0.05507956	0.4325187	0.2282752
nArOR	number of ethers (aromatic)	Functional group counts	1.725e-10	0.34375	0.5973072	0.5609754	0.828797
T(O..F)	sum of topological distances between O..F	2D Atom Pairs	2.04e-06	7.319444	4.701346	28.60066	16.85067
nCconj	number of non-aromatic conjugated C(sp^2)	Functional group counts	< 2.2e-16	1.007639	0.623011	1.09675	0.9987047
Hy	hydrophilic factor	Molecular properties	< 2.2e-16	0.8010174	0.5765557	1.004835	1.616969

Supplementary Material

Table S4. Definition, type, Mann-Whitney test *p*-value, mean, and standard deviation of 17 VIP-selected molecular descriptors for active/inactive classification of CDK4 molecules.

Selected molecular descriptors	Definition	Type	Mann–Whitney test *p*-value	Mean value for active CDK4 Molecules	Mean value for inactive CDK4 Molecules	Std value for active CDK4 Molecules	std value for inactive CDK4 Molecules
N-070	Ar-NH-Al	Atom-centered fragments	< 2.2e-16	0.1554286	0.3951049	0.4209362	0.6051044
nCbH	number of unsubstituted benzene C(sp^2)	Functional group counts	< 2.2e-16	2.934857	5.993007	3.681434	4.19523
C-032	X--CX--X	Atom-centered fragments	< 2.2e-16	0.6731429	0.2097902	0.478985	0.4247294
nBnz	number of benzene-like rings	Ring descriptors	3.677e-10	0.8857143	1.653846	0.9736643	1.070712
N-075	R--N--R / R--N--X	Atom-centered fragments	< 2.2e-16	2.876571	1.251748	1.758494	1.50073
T(N..N)	sum of topological distances between N..N	2D Atom Pairs	< 2.2e-16	115.9291	37.11888	80.65901	43.23652
nPyridines	number of Pyridines	Functional group counts	< 2.2e-16	0.8422857	0.2412587	0.7209162	0.4825122
C-027	R--CH--X	Atom-centered fragments	< 2.2e-16	1.673143	0.6118881	1.400604	0.9910469
nPyrimidines	number of Pyrimidines	Functional group counts	< 2.2e-16	0.7234286	0.3216783	0.4796425	0.4753783
nRNHR	number of secondary amines (aliphatic)	Functional group counts	< 2.2e-16	0.3097143	0.08741259	0.4626402	0.2829338
nCb-	number of substituted benzene C(sp^2)	Functional group counts	< 2.2e-16	2.349714	3.86014	2.42484	2.517591
N-067	Al2-NH	Atom-centered fragments	< 2.2e-16	0.3154286	0.1013986	0.4649521	0.3023849
H-049	H attached to C3(sp^3)/C2(sp^2)/C3(sp^2)/C3(sp)	Atom-centered fragments	< 2.2e-16	1.76	0.8251748	1.388742	1.132082
C-043	X--CR..X	Atom-centered fragments	< 2.2e-16	0.6628571	0.2727273	0.496605	0.4461424
N-073	Ar2NH / Ar3N / Ar2N-Al / R..N..R	Atom-centered fragments	< 2.2e-16	1.514286	0.8881119	0.75528	0.7216443
nCrs	number of ring secondary C(sp^3)	Functional group counts	< 2.2e-16	3.337143	0.9965035	3.217434	1.672268
W	detour index	topological descriptors	< 2.2e-16	6846.894	4487.024	2838.616	2274.042

Supplementary Material

Table S5. Definition, type, Mann-Whitney test *p*-value, mean, and standard deviation of 25 VIP-selected molecular descriptors for active/inactive classification of CDK5 molecules.

Selected molecular descriptors	Definition	Type	Mann–Whitney test *p*-value	Mean value for active CDK5 Molecules	Mean value for inactive CDK5 Molecules	Std value for active CDK5 Molecules	std value for inactive CDK5 Molecules
N-072	RCO-N< / >N-X=X	Atom-centered fragments	< 2.2e-16	1.486154	0.5899582	1.294992	0.7429622
O-060	Al-O-Ar / Ar-O-Ar / R..O..R / R-O-C=X	Atom-centered fragments	< 2.2e-16	3.089231	0.8117155	6.551071	3.808726
T(S..S)	sum of topological distances between S..S	2D Atom Pairs	< 2.2e-16	3.089231	0.8117155	6.551071	3.808726
nArOR	number of ethers (aromatic)	Functional group counts	< 2.2e-16	0.1169231	0.5020921	0.378778	0.8819542
C-026	R--CX--R	Atom-centered fragments	< 2.2e-16	1.033846	1.561506	1.124907	1.330681
H-047	H attached to $C1(sp^3)/C0(sp^2)$	Atom-centered fragments	0.003089	9.012308	9.748954	4.210908	4.581907
nRCONHR	number of secondary amides (aliphatic)	Functional group counts	< 2.2e-16	0.4123077	0.2125523	0.5259104	0.4213837
nHDon	number of donor atoms for H-bonds (N and O)	Functional group counts	< 2.2e-16	2.821538	1.884519	1.178452	1.164114
Hy	hydrophilic factor	Molecular properties	< 2.2e-16	0.8952262	0.312851	0.8320551	0.8146602
TPSA(Tot)	topological polar surface area using N, O, S, P polar contributions	Molecular properties	< 2.2e-16	128.7498	84.69926	53.85546	35.66826
C-044	X--CX..X	Atom-centered fragments	< 2.2e-16	0.5123077	0.08786611	0.7696091	0.3296794
AROM	aromaticity index	Geometrical descriptors	< 2.2e-16	0.86322	0.9171816	0.09327503	0.08160972
nThiazoles	number of Thiazoles	Functional group counts	< 2.2e-16	0.2923077	0.1054393	0.4717955	0.3153189
GNar	Narumi geometric topological index	Topological indices	< 2.2e-16	2.083211	2.119558	0.06994312	0.07732465
nCar	number of aromatic $C(sp^2)$	Functional group counts	0.007083	12.83385	13.03849	4.224088	3.601859
Rww	reciprocal hyper-detour index	topological descriptors.	< 2.2e-16	26.0307	16.07522	13.01385	8.515935
nBnz	number of benzene-like rings	Ring descriptors	4.342e-12	1.16	1.400837	0.7665584	0.7507669
LP1	Lovasz-Pelikan index (leading eigenvalue)	eigenvalue–based indices	1.744e-07	2.483775	2.495403	0.05574758	0.06636554
nCs	number of total secondary $C(sp^3)$	Functional group counts	< 2.2e-16	2.466154	1.03431	2.324836	1.742078
RNCG	relative negative charge	Charge descriptors	< 2.2e-16	0.1615092	0.2129013	0.05664406	0.05903639
STN	Sum of tN E-states	Atom-type E-state indices	0.3973	6.670411	6.630134	1.83208	1.673812
RBF	rotatable bond fraction	Constitutional indices	< 2.2e-16	0.1060708	0.07445272	0.04867696	0.04375798
nPyrroles	number of Pyrroles	Functional group counts	< 2.2e-16	0.1476923	0.3723849	0.3921839	0.5330679
PHI	Kier flexibility index	Topological indices	< 2.2e-16	6.217555	4.32925	2.655207	1.656357
PCR	ratio of multiple path count over path count	Walk and path counts	< 2.2e-16	1.502554	1.543071	0.08792831	0.1025405

Supplementary Material

Table S6. Definition, type, Mann-Whitney test *p*-value, mean, and standard deviation of 21 VIP-selected molecular descriptors for active/inactive classification of CDK9 molecules.

Selected molecular descriptors	Definition	Type	Mann–Whitney test *p*-value	Mean value for active CDK9 Molecules	Mean value for inactive CDK9 Molecules	Std value for active CDK9 Molecules	std value for inactive CDK9 Molecules
C-029	R--CX--X	Atom-centered fragments	2.2e-16	0.3197172	0.7077703	0.4863507	0.5881576
F-084	F attached to C1(sp^2)	Atom-centered fragments	< 2.2e-16	0.974077	0.2736486	0.806881	0.6040878
nArX	number of X on aromatic ring	Functional group counts	< 2.2e-16	1.19403	0.5574324	0.8053841	0.7848473
H-051	H attached to alpha-C	Atom-centered fragments	< 2.2e-16	1.017282	0.4814189	1.614016	1.177766
nF	number of Fluorine atoms	Constitutional indices	< 2.2e-16	1.088767	0.4881757	1.034381	0.9469134
nPyrroles	number of Pyrroles	Functional group counts	< 2.2e-16	0.615868	0.2567568	0.4898012	0.4849215
nPyridines	number of Pyridines	Functional group counts	< 2.2e-16	0.9010212	0.4493243	0.5548023	0.5967761
nPyrimidines	number of Pyrimidines	Functional group counts	< 2.2e-16	0.1649647	0.4290541	0.3734059	0.4953596
nX	number of halogen atoms	Constitutional indices	< 2.2e-16	1.307148	0.7753378	1.009742	1.059259
nCbH	number of unsubstituted benzene C(sp^2)	Functional group counts	< 2.2e-16	3.802042	5.248311	2.361263	3.467153
C-032	X--CX--X	Atom-centered fragments	0.004213	0.2003142	0.1452703	0.4003928	0.3526711
nArNHR	number of secondary amines (aromatic)	Functional group counts	7.562e-13	0.3778476	0.5929054	0.5103144	0.616867
T(O..F)	sum of topological distances between O..F	2D Atom Pairs	< 2.2e-16	23.15082	9.550676	28.41111	21.15739
C-043	X--CR..X	Atom-centered fragments	< 2.2e-16	0.6849961	0.3783784	0.4780426	0.4923153
C-027	R--CH--X	Atom-centered fragments	< 2.2e-16	1.175177	0.7820946	0.7332702	0.8027978
T(N..F)	sum of topological distances between N..F	2D Atom Pairs	< 2.2e-16	42.74548	20.34122	42.65027	50.18941
T(F..F)	sum of topological distances between F..F	2D Atom Pairs	1.521e-13	3.714061	0.9611486	15.29505	4.794615
T(S..F)	sum of topological distances between S..F	2D Atom Pairs	3.597e-13	4.528672	1.564189	11.25271	6.614546
C-034	R--CR..X	Atom-centered fragments	< 2.2e-16	0.7981147	0.5878378	0.4371825	0.6701673
nR=Ct	number of aliphatic tertiary C(sp^2)	Functional group counts	< 2.2e-16	0.351139	0.1655405	0.4905081	0.4309848
C-030	X--CH--X	Atom-centered fragments	< 2.2e-16	0.08091123	0.2466216	0.2728059	0.4314091

Supplementary Material

Table S7. The confusion matrix of the CPANN and SKN active/inactive models for the binary classification of CDK molecules for the training and prediction sets.

Model	Target	Predicted/real	Training set		Prediction set	
			Active	Inactive	Active	Inactive
SKN	CDK1	Active	**492**	57	**439**	110
		Inactive	62	**412**	117	**357**
CPANN	CDK1	Active	**504**	45	**469**	80
		Inactive	83	**391**	132	**342**
SKN	CDK2	Active	**976**	40	**912**	104
		Inactive	59	**505**	134	**430**
CPANN	CDK2	Active	**966**	50	**922**	94
		Inactive	108	**456**	163	**401**
SKN	CDK4	Active	**603**	14	**592**	25
		Inactive	21	**175**	34	**162**
CPANN	CDK4	Active	**966**	50	**591**	26
		Inactive	108	**456**	40	**156**
SKN	CDK5	Active	**409**	49	**347**	111
		Inactive	49	**784**	112	**721**
CPANN	CDK5	Active	**422**	36	**370**	88
		Inactive	89	**744**	129	**704**
SKN	CDK9	Active	**846**	48	**812**	82
		Inactive	62	**350**	86	**326**
CPANN	CDK9	Active	**867**	27	**831**	63
		Inactive	68	**344**	104	**308**

Table S8. The applicability domains calculated using the leverage approach for binary active/inactive classification of CDK molecules together with the applicability domain calculated for classification of molecules based on their therapeutic targets.

	Number of "in domain" train\` set molecules	Number of "out domain" test set molecules	Leverage
active/inactive CDK1	**960 (93.8%)/1023**	**63(6.1%)/438**	**0.0616**
active/inactive CDK2	**653(96.4%)/1580**	**24(3.5%)/677**	**0.0456**
active/inactive CDK4	**321(92.2%)/813**	**27(7.7%)/348**	**0.0664**
active/inactive CDK5	**544(98.1%)/1291**	**10(1.8%)/554**	**0.0604**
active/inactive CDK9	**519(92.8%)/1306**	**40(7.1%)/559**	**0.0505**
multi-class classification	**1464(97.4%)/3508**	**39(7.1%)/1503**	**0.0291**

Supplementary Material

Table S9. The mean values of error rate, non-error rate, accuracy, and AUC for the CPANN and SKN models after 100 iterations of y-randomization tests

Target	Model	Error rate	Non-error rate	Accuracy	AUC
		Training/ Cross Validation/ Test	Training/ Cross Validation/ Test	Training/ Cross Validation/ Test	
CDK1	CPANN	0.296/ 0.527/ 0.485	0.703/ 0.472/ 0.514	0.714/ 0.482/ 0.518	0.62
	SKN	0.627/ 0.465/ 0.564	0.618/ 0.534/ 0.435	0.381/ 0.542/ 0.438	0.60
CDK2	CPANN	0.436/ 0.495/ 0.463	0.563/ 0.504/ 0.536	0.668/ 0.608/ 0.627	0.64
	SKN	0.381/ 0.516/ 0.481	0.618/ 0.483/0.518	0.675/ 0.593/ 0.567	0.62
CDK4	CPANN	0.428/ 0.480/ 0.519	0.571/ 0.519/ 0.480	0.785/ 0.746/ 0.701	0.62
	SKN	0.456/ 0.498/ 0.494	0.543/ 0.501/ 0.505	0.774/ 0.738/ 0.732	0.55
CDK5	CPANN	0.420/ 0.501/ 0.513	0.579/ 0.498/ 0.486	0.665/ 0.586/ 0.586	0.66
	SKN	0.412/ 0.497/ 0.539	0.587/ 0.502/ 0.460	0.675/ 0.580/ 0.570	0.64
CDK9	CPANN	0.424/ 0.495/ 0.514	0.575/ 0.504/ 0.485	0.686/ 0.599/ 0.608	0.58
	SKN	0.414/ 0.503/ 0.511	0.585/ 0.496/ 0.48	0.676/ 0.593/ 0.586	0.57
Multi-Class model for Actives	CPANN	0.722/ 0.795/0.778	0.277/ 0.204/0.221	0.339/ 0.260/0.285	(CDK1):0.66/(CDK2):0.63 / (CDK4): 0.62/ (CDK5): 0.66 / (CDK9): 0.63
	SKN	0.727/ 0.794/ 0.798	0.272/ 0.205/0.201	0.309/ 0.235/ 0.232	(CDK1):0.55/(CDK2):0.56 /(CDK4): 0.54/ (CDK5): 0.53/ (CDK9):0.55

Table S10. The statistical results for the developed binary active/inactive classifiers for different CDK groups. The KNN and SVM models were evaluated in terms of sensitivity, specificity, precision, accuracy, and Mathew-correlation coefficient (MCC).

Model	Class	Precision	Sensitivity	Specificity	Non-error rate	Accuracy	MCC*
		Training/ Cross Validation/ Test	Training/ Cross Validation/ Test	Training/ Cross Validation/ Test	Training/ Cross Validation/ Test	Training/ Cross Validation/ Test	Training/ Cross Validation/ Test
CDK1							
KNN	Active	0.665/ 0.642/ 0.542	0.672/ 0.650/ 0.561	0.650/ 0.615/ 0.530	0.643/ 0.650/ 0.561	0.647/ 0.623/ 0.571	0.527/ 0.461/ 0.430
	Inactive	0.661/ 0.640/ 0.539	0.650/ 0.615/ 0.530	0.672/ 0.650/ 0.561			
SVM	Active	0. 916/ 0.795/ 0.882	0.930/ 0.830/ 0.848	0.884/ 0.753/ 0.738	0.870/ 0.781/ 0.804	0.869/ 0.790/ 0.761	0.709/ 0.555/ 0.509
	Inactive	0.902/ 0.793/ 0.772	0.884/ 0.753/ 0.738	0.930/ 0.830/ 0.878			
CDK2							
KNN	Active	0.666/ 0.664/ 0.585	0.618/ 0.611/ 0.612	0.544/ 0.542/ 0.502	0.631/ 0.627/ 0.612	0.656/ 0.651/ 0.615	0.651/ 0.630/0.523
	Inactive	0.635/ 0.623/ 0.645	0.544/ 0.542/ 0.502	0.618/ 0.611/ 0.612			
SVM	Active	0.960/ 0.884/ 0.880	0.974/ 0.907/ 0.900	0.932/ 0.785/ 0.814	0.953/ 0.846/ 0.857	0.905/ 0.863/0.851	0.751/ 0.700/ 0.641
	Inactive	0.950/ 825/ 0.830	0.932/ 0.785/ 0.814	0.974/ 0.907/0.900			
CDK4							
KNN	Active	0.647/ 0.642/ 0.643	0.664/ 0.662/ 0.669	0.531/ 0.516/ 0.533	0.698/ 0.689/ 0.601	0.632/ 0.627/ 0.633	0.612/ 0.597/ 0.524
	Inactive	0.581/ 0.574/ 0.503	0.531/ 0.516/ 0.833	0.664/ 0.662/ 0.669			
SVM	Active	0.948/ 0.946/ 0.942	0.959/ 0.949/ 0.945	0.846/ 0.795/ 0.788	0.836/ 0.890/ 0.889	0.929/ 0.921/ 0.916	0.806/ 0.784/ 0.781
	Inactive	0.867/ 0.840/ 0.842	0.836/ 0.831/ 0.833	0.977/ 0.957/ 0.965			
CDK5							
KNN	Active	0.624/ 0.600/ 0.611	0.656/ 0.646/ 0.618	0.667/ 0.650/0.611	0.594/ 0.583/ 0.544	0.594/ 0.583/ 0.544	0.536/ 0.515/ 0.450
	Inactive	0.526/ 0.523/ 0.559	0.567/ 0.550/ 0.511	0.556/ 0.546/ 0.518			
SVM	Active	0.892/ 0.788/ 0.734	0.927/ 0.821/ 0.822	0.918/ 0.777/ 0.742	0.933/ 0.849/ 0.832	0.934/ 0.857/ 0.835	0.756/ 0.691/ 0.521
	Inactive	0.859/ 0.699/ 0.699	0.918/ 0.777/ 0.742	0.927/ 0.821/ 0.822			
CDK9							
KNN	Active	0.782/ 0.681/ 0.515	0.731/ 0.737 / 0.610	0.630/ 0.625/ 0.602	0.701/ 0.631/ 0.566	0.715/ 0.671/ 0.581	0623/ 0.592/ 0.530
	Inactive	0.731/ 0.642/ 0.513	0.630/ 0.625/ 0.602	0.731/ 0.737/ 0.610			
SVM	Active	0.838/ 0.797/ 0.789	0.862/ 0.830/ 0.794	0.865/ 0.769/ 0.666	0.813/ 0.820/ 0.730	0.831/ 0.859/ 0.753	0.738/ 0.676/ 0.603
	Inactive	0.812/0.736/0.675	0.854/0.769/0.666	0.862/0.830/0.794			

Supplementary Material

Table S11. Common molecular descriptors appeared in active/inactive models for different CDK groups. For example, two descriptors of Hy and nRCONHR were common in three active/inactive binary classifiers for CDK1, CDK2, CDK5 targets.

Targets Name	Number of Common Descriptors	Common Descriptors Name
CDK1, CDK2, CDK5	**2**	Hy, nRCONHR
CDK1, CDK4, CDK9	**1**	C-043
CDK1, CDK2	**4**	nCconj, S-110, nS, T(N..S)
CDK1, CDK5	**1**	N-072
CDK1, CDK9	**4**	T (N.. F), nR=Ct, H-051, nF
CDK2, CDK5	**3**	O-060, nArOR, TPSA(Tot)
CDK2, CDK9	**4**	C-029, F-084, T (O.. F), C-034
CDK4, CDK5	**1**	nBnz
CDK4, CDK9	**5**	C-027, nCbH, nPyridines, nPyrimidines, C-032
CDK5, CDK9	**1**	nPyrroles

Table S12. The results of SKN and CPANN models for multi-class classification of active CDK inhibitors in the training, cross-validation, and test sets. (Note: the multi-class SKN and CPANN models were developed to categorize active CDK molecules based on their therapeutic targets.)

Model	Class	Precision	Sensitivity	Specificity	Non-error rate	Accuracy	AUC
		Training/ Cross Validation/ Test	Training/ Cross Validation/ Test	Training/ Cross Validation/ Test	Training/ Cross Validation/ Test	Training/ Cross Validation/ Test	
SKN	Actives CDK1	0.843/ 0.763/ 0.843	0.891/ 0. 700/ 0.891	0.970/ 0.960/ 0.970	0.923/ 0.804/ 0.923	0.925/ 0.816/ 0.925	0.93
	Actives CDK 2	0.946/ 0.807/ 0.946	0.918/ 0.825/ 0.918	0.978/ 0.918/ 0.978			0.95
	Actives CDK4	0.946/ 0.845/ 0.946	0.929/ 0.837/ 0.929	0.990/ 0.972/ 0.990			0.96
	Actives CDK5	0.896/ 0.764/ 0.896	0.931/ 0.779/ 0.931	0.983/ 0.962/ 0.983			0.96
	Actives CDK9	0.957/ 0.864/ 0.957	0.947/ 0.880/ 0.947	0.984/ 0.950/ 0.984			0.97
CPANN	Actives CDK1	0.788/ 0.707/ 0.696	0.753/ 0.707/ 0.739	0.962/ 0.946/ 0.941	0.841/ 0.798/ 0.816	0.852/ 0.811/ 0.822	0.97
	Actives CDK2	0.817/ 0.775/ 0.785	0.880/ 0.820/ 0.823	0.921/ 0.905/ 0.906			0.98
	Actives CDK4	0.850/ 0.838/ 0.857	0.915/ 0.896/ 0.894	0.963/ 0.960/ 0.973			0.99
	Actives CDK5	0.834/ 0.774/ 0.786	0.791/ 0.722/ 0.774	0.977/ 0.969/ 0.966			0.99
	Actives CDK9	0.953/0.923/ 0.952	0.867/ 0.846/ 0.852	0.986/ 0.976/ 0.984			0.99

Supplementary Material

Table S13. The confusion matrix of the CPANN and SKN multi-class classifiers for the training and prediction sets. (Note: the multi-class classifiers were developed to categorize active CDK molecules based on their therapeutic targets.)

Model	Predicted/real	Training set					Predicted/real	Prediction set				
		Actives CDK1	Actives CDK2	Actives CDK4	Actives CDK5	Actives CDK9		Actives CDK1	Actives CDK2	Actives CDK4	Actives CDK5	Acti CDK
CPANN	Actives CDK1	**409**	54	51	28	1	Actives CDK1	**170**	26	15	16	3
	Actives CDK2	44	**880**	10	34	31	Actives CDK2	41	**363**	5	19	13
	Actives CDK4	19	32	**592**	3	1	Actives CDK4	11	12	**204**	1	0
	Actives CDK5	27	60	2	**353**	4	Actives CDK5	14	28	3	**158**	1
	Actives CDK9	20	50	41	5	**757**	Actives CDK9	8	33	11	7	**341**
SKN	Actives CDK1	**205**	12	5	6	2	Actives CDK1	**170**	26	15	16	3
	Actives CDK2	14	**405**	1	12	9	Actives CDK2	41	**363**	5	19	13
	Actives CDK4	11	2	**212**	0	3	Actives CDK4	11	12	**204**	1	0
	Actives CDK5	6	3	2	**190**	3	Actives CDK5	14	28	3	**158**	1
	Actives CDK9	7	6	4	4	**379**	Actives CDK9	8	33	11	7	**341**

Table S14. The results of KKN and SVM models for multi-class classification of active CDK inhibitors in the training, validation, and test sets. (Note: the multi-class SKN and CPANN models were developed to categorize active CDK molecules based on their therapeutic targets.)

Model	Class	Precision	Sensitivity	Specificity	Non-error rate	Accuracy
		Training/ Cross Validation/ Test	Training/ Cross Validation/ Test	Training/ Cross Validation/ Test	Training/ Cross Validation/ Test	Training/ Cross Validation/ Test
KKN	Actives CDK1	0.643/ 0.563/ 0.543	0.691/ 0.600/ 0.491	0.770/ 0.660/ 0.570	0.623/ 0.604/ 0.523	0.625/ 0.616/ 0.525
	Actives CDK 2	0.746/ 0.707/ 0.646	0.628/ 0.615/ 0.618	0.678/ 0.618/ 0.578		
	Actives CDK4	0.646/ 0.645/ 0.624	0.629/ 0.613/ 0.601	0.690/ 0.612/ 0.590		
	Actives CDK5	0.606/ 0.604/ 0.601	0.631/ 0624/ 0.603	0.613/ 0.602/ 0.583		
	Actives CDK9	0.657/ 0.644/ 0.557	0.647/ 0.608/ 0.547	0.684/ 0.650/ 0.584		
SVM *	Actives CDK1	0.768/ 0.677/ 0.656	0.743/ 0.737/ 0.723	0.862/ 0.846/ 0.841	0.830/ 0.758/ 0.720	0.755/ 0.728/ 0.721
	Actives CDK2	0.827/ 0.764/ 0.761	0.780/ 0.720/ 0.713	0.821/ 0.805/ 0.801		
	Actives CDK4	0.821/ 0.738/ 0.720	0.815/ 0.796/ 0.793	0.863/ 0.860/ 0.773		
	Actives CDK5	0.824/ 0.750/ 0.726	0.691/ 0.622/ 0.614	0.817/ 0.819/ 0.776		
	Actives CDK9	0.853/0.820/ 0.812	0.854/ 0.746/ 0.742	0.886/ 0.776/ 0.764		

* Note: For multi-class classification using SVM, a one-against-one strategy was applied and the decision about multiple classes was achieved by majority voting among all five binary models.

Supplementary Material

Table S15. Definition, type, Kruskal Wallis test *p*-value, mean, and standard deviation of 31 VIP-selected molecular descriptors for multiclassification of active CDK inhibitors.

Selected molecular descriptors	Definition	Type	Kruskal Wallis test *p*-value	Average value for active CDK1	Std value for active CDK1	Average value for active CDK2	Std value for active CDK2	Average value for active CDK4	Std value for active CDK 4	Average value for active CDK5	Std value for active CDK5	Average value for active CDK9	Std value for active CDK9
nPyrazoles	number of Pyrazoles	Functional group counts	< 2.2e-16	0.1306598	0.3448424	0.4375	0.571731	0.03771429	0.1906133	0.06461538	0.258257	0.08405342	0.2886837
nPyridines	number of Pyridines	Functional group counts	< 2.2e-16	0.2548512	0.5074501	0.2208333	0.4345846	1.121143	0.6551668	0.3184615	0.5012749	0.9010212	0.5548023
nPyrimidines	number of Pyrimidines	Functional group counts	< 2.2e-16	0.6274256	0.4864732	0.3256944	0.4862596	0.9017143	0.3572515	0.2692308	0.5351794	0.1649647	0.3734059
nCb-	number of substituted benzene C(sp^2)	Functional group counts	< 2.2e-16	3.39586	2.117307	3.779861	2.679189	1.444571	1.967721	2.376923	1.966066	3.216025	1.786478
nBnz	number of benzene-like rings	Ring descriptors	< 2.2e-16	1.345408	0.7151001	1.440278	0.8364789	0.4891429	0.6821078	1.16	0.7665584	1.169678	0.645101
nPyrroles	number of Pyrroles	Functional group counts	< 2.2e-16	0.1772316	0.4505599	0.2423611	0.5353494	0.6422857	0.493708	0.1476923	0.3921839	0.615868	0.4898012
nCbH	number of unsubstituted benzene C(sp^2)	Functional group counts	< 2.2e-16	4.645537	2.566443	4.739583	2.788259	1.483429	2.462652	4.543077	3.210464	3.802042	2.361263
C-029	R--CX--X	Atom-centered fragments	< 2.2e-16	0.6274256	0.6115242	0.2479167	0.4630126	1.107429	0.6345341	0.3784615	0.5220832	0.3197172	0.4863507
nArX	number of X on aromatic ring	Functional group counts	< 2.2e-16	0.5692109	0.6993217	0.4944444	0.9394288	0.52	0.8007434	0.3092308	0.5394236	1.19403	0.8053841
N-070	Ar-NH-Al	Atom-centered fragments	< 2.2e-16	0.5692109	0.6993217	0.1625	0.4117582	0.04685714	0.222012	0.3492308	0.5923558	0.2081697	0.4305867
S-107	R2S / RS-SR	Atom-centered fragments	< 2.2e-16	0.0931436	0.3284712	0.2118056	0.5141496	0.04342857	0.2201251	0.8184615	0.9458192	0.03534957	0.2232713
TPSA(Tot)	topological polar surface area using N,O,S,P polar contributions	Molecular properties	< 2.2e-16	100.496	27.31063	102.618	34.11922	97.23442	21.26354	128.7498	53.85546	86.01498	26.12662
H-049	H attached to C3(sp^3)/C2(sp^2)/C3(sp^2)/C3(sp)	Atom-centered fragments	< 2.2e-16	0.9754204	0.9151382	0.8673611	0.9496226	2.260571	1.28741	0.9292308	1.119063	1.339356	0.7210875
nR=Ct	number of aliphatic tertiary C(sp^2)	Functional group counts	< 2.2e-16	0.05692109	0.2632388	0.06458333	0.259622	0.06057143	0.2701591	0.1261538	0.4147797	0.351139	0.4905081
nN	number of Nitrogen atoms	Constitutional indices	< 2.2e-16	5.335058	1.848617	4.701389	1.643653	7.491429	1.747403	5.481538	2.085343	4.581304	1.336426
nCrt	number of ring tertiary C(sp^3)	Functional group counts	< 2.2e-16	0.1060802	0.389801	0.2458333	0.4737553	0.2102857	0.4986336	0.06153846	0.2468241	0.4728987	0.6112899
TPSA(NO)	topological polar surface area using N, O polar contributions	Molecular properties	< 2.2e-16	95.47292	25.81434	94.92551	31.13264	95.62955	21.58207	104.2966	33.75166	82.79189	22.83407
nX	number of halogen atoms	Constitutional indices	< 2.2e-16	0.44292	25.81434	0.6006944	1.072558	0.5897143	0.8998522	0.4923077	0.7616193	1.307148	1.009742
nArNHR	number of secondary amines (aromatic)	Functional group counts	< 2.2e-16	0.534282	0.7165164	0.1625	0.4117582	0.04685714	0.222012	0.3492308	0.5923558	0.2081697	0.4305867
nF	number of Fluorine atoms	Constitutional indices	< 2.2e-16	0.4877102	1.025032	0.3673611	0.8973229	0.5485714	0.8928176	0.1323077	0.5187123	1.088767	1.034381
T(N..N)	sum of topological distances between N..N	2D Atom Pairs	< 2.2e-16	63.75809	59.50033	50.0375	55.36645	154.3086	67.08459	67.80769	67.61366	51.8586	51.41656
nRNHR	number of secondary amines (aliphatic)	Functional group counts	< 2.2e-16	0.07891332	0.269778	0.06041667	0.241238	0.4297143	0.4953184	0.02307692	0.1502635	0.2458759	0.4307745
nHDon	number of donor atoms for H-bonds (N and O)	Functional group counts	< 2.2e-16	2.874515	1.300074	2.671528	1.385829	1.945143	0.9706015	2.821538	1.178452	2.133543	0.8482769
nR05	number of 5-membered rings	Ring descriptors	< 2.2e-16	1.260026	0.8850623	1.434028	0.866219	1.652571	0.8452192	1.5	0.6759118	1.05813	0.6229989
T(N..S)	sum of topological distances between N..S	2D Atom Pairs	< 2.2e-16	8.836999	15.69873	10.21736	17.67746	2.723429	12.32266	24.74308	41.41035	7.863315	16.7144
nCconj	number of non-aromatic conjugated C(sp^2)	Functional group counts	< 2.2e-16	0.6714101	1.188534	1.007639	1.09675	0.6525714	1.222586	1.181538	1.419965	0.8931658	1.168733

T(O..F)	sum of topological distances between O..F	2D Atom Pairs	< 2.2e-16	6.573092	22.53331	7.319444	28.60066	5.816	17.59321	1.616923	7.924685	23.15082	28.41111
Ms	mean topological state	It is among the constitutional descriptors	< 2.2e-16	2.351229	0.2813562	2.386424	0.2599491	2.158731	0.1945031	2.381615	0.1891951	2.368452	0.2442635
T(N..O)	sum of topological distances between N..O	2D Atom Pairs	5.011e-07	58.27555	47.71814	70.92222	87.56717	64.56	68.68143	88.36923	95.93727	63.86489	59.41586
T(O..S)	sum of topological distances between O..S	2D Atom Pairs	< 2.2e-16	5.278137	10.28148	6.256944	14.96846	0.6182857	4.298436	19.98	34.18203	3.711705	8.539181
nS	number of Sulfur atoms	Constitutional indices	< 2.2e-16	0.3661061	0.5614932	0.4104167	0.6416426	0.07542857	0.2769186	0.9107692	1.004477	0.2835821	0.4989193

Table S16. The area under the receiver characteristic curves and EF values obtained for screening of PubChem-R database using SKN and CPANN active/inactive classifiers.

CDK	Model	AUC	$EF_{1\%}$	$EF_{10\%}$
CDK1	CPANN	0.6749	33.1177	3.7775
	SKN	0.8213	73.4799	7.3480
CDK2	CPANN	0.6978	39.5833	3.9583
	SKN	0.9319	89.3056	8.9306
CDK4	CPANN	1	100	10
	SKN	1	100	10
CDK5	CPANN	0.7078	22.9231	2.6923
	SKN	0.7217	18.1538	2.2
CDK9	CPANN	0.8207	52.2388	5.7188
	SKN	0.9144	85.8602	8.5860
Average	CPANN	0.78024	49.57258	5.22938
	SKN	0.87786	73.3599	7.41292

Supplementary Material

Table S17. The area under the receiver characteristic curves and the EF values obtained for screening of PubChem-R database using SKN and CPANN multi-class classifiers. (Note: the multi-class classifiers were developed to categorize active CDK molecules based on their therapeutic targets.)

CDK	Model	AUC	EF $_{1\%}$	EF $_{10\%}$
**CDK1**	CPANN	**0.6539**	**15.6533**	**3.1307**
	SKN	**0.8401**	**48.9004**	**5.7309**
**CDK2**	CPANN	**0.6690**	**20.4167**	**3.4028**
	SKN	**0.8622**	**64.7222**	**6.9444**
**CDK4**	CPANN	**0.8544**	**41.8286**	**6.6400**
	SKN	**0.9659**	**77.1429**	**7.9314**
**CDK5**	CPANN	**0.7333**	**30.6154**	**3.6923**
	SKN	**0.9047**	**64.3077**	**6.5231**
**CDK9**	CPANN	**0.8790**	**62.5295**	**6.6771**
	SKN	**0.9328**	**78.7903**	**8.0361**
**Average**	CPANN	**0.75792**	**34.2087**	**4.70858**
	SKN	**0.90114**	**66.7727**	**7.03316**

Supplementary Material

References:

1. Sonawane YA, Taylor MA, Napoleon JV, Rana S, Contreras JI, Natarajan A. Cyclin Dependent Kinase 9 Inhibitors for Cancer Therapy. Journal of Medicinal Chemistry. 2016;59(19):8667-84.
2. Shi Y, Park J, Lagisetti C, Zhou W, Sambucetti LC, Webb TR. A triple exon-skipping luciferase reporter assay identifies a new CLK inhibitor pharmacophore. Bioorganic & Medicinal Chemistry Letters. 2017;27(3):406-12.
3. Lee J, Park T, Jeong S, Kim K-H, Hong C. 3-Hydroxychromones as cyclin-dependent kinase inhibitors: Synthesis and biological evaluation. Bioorganic & Medicinal Chemistry Letters. 2007;17(5):1284-7.
4. Nekardová M, Vymětalová L, Khirsariya P, Kováčová S, Hylsová M, Jorda R, et al. Structural basis of the interaction of cyclin-dependent kinase 2 with roscovitine and its analogues having bioisosteric central heterocycles. ChemPhysChem. 2017;18(7):785-95.
5. Stevens KL, Reno MJ, Alberti JB, Price DJ, Kane-Carson LS, Knick VB, et al. Synthesis and evaluation of pyrazolo[1,5-b]pyridazines as selective cyclin dependent kinase inhibitors. Bioorganic & Medicinal Chemistry Letters. 2008;18(21):5758-62.
6. Aubry C, Wilson AJ, Emmerson D, Murphy E, Chan YY, Dickens MP, et al. Fascaplysin-inspired diindolyls as selective inhibitors of CDK4/cyclin D1. Bioorganic & Medicinal Chemistry. 2009;17(16):6073-84.
7. Teng M, Jiang J, He Z, Kwiatkowski NP, Donovan KA, Mills CE, et al. Development of CDK2 and CDK5 dual degrader TMX-2172. Angewandte Chemie. 2020;132(33):13969-74.
8. Kaller MR, Zhong W, Henley C, Magal E, Nguyen T, Powers D, et al. Design and synthesis of 6-oxo-1,6-dihydropyridines as CDK5 inhibitors. Bioorganic & Medicinal Chemistry Letters. 2009;19(23):6591-4.
9. Czudor Z, Balogh M, Bánhegyi P, Boros S, Breza N, Dobos J, et al. Novel compounds with potent CDK9 inhibitory activity for the treatment of myeloma. Bioorganic & Medicinal Chemistry Letters. 2018;28(4):769-73.
10. Gao J, Fang C, Xiao Z, Huang L, Chen C-H, Wang L-T, et al. Discovery of novel 5-fluoro-N2,N4-diphenylpyrimidine-2,4-diamines as potent inhibitors against CDK2 and CDK9. MedChemComm. 2015;6(3):444-54.

Appendix A

Appendix A

In order to conduct a comprehensive analysis of similarity of the training and test set molecules, we examined the relationships between each member of the test set and its nearest counterpart in the training set, in this work. By evaluating the similarity of each test molecule against every training molecule and identifying the training molecule with the highest similarity for each test instance, a meticulous comparison was achieved. We calculated the similarity of molecules using their Euclidean distances by the following formula:

$$D_{A,B} = \left[\sum_{j=1}^{n}(x_{jA} - x_{jB})^2\right]^{\frac{1}{2}}$$

Each vector, A and B, represent a typical molecule and consists of n elements or components, denoted as x_j^A and x_j^B respectively, where j ranges from 1 to n. For each dimension j, the formula computes the difference between the corresponding elements of the two vectors: $(x_j^A - x_j^B)$[h]. The similarities and distances were interconverted using the following equation:

$$\text{Similarity} = \frac{1}{1 + distance}$$

It is worth to mention that, we built active/inactive CDK1, CDK2, CDK4, CDK5, and CDK9 models using 20, 23, 17, 25, and 21 VIP-selected descriptors, respectively. In addition, we constructed the active multiclass model by 31 VIP-selected descriptors. We used these descriptors for calculation of similarity values first. We then further expanded the descriptor space to 208, 207, 195, 205, 204, and 198 to those descriptor sets before the VIP variable selection procedure. Finally, we made comparison between the similarity distributions obtained in both descriptor spaces.

As mentioned above, we calculated the similarity between each molecule in test set and all molecules in the training set. Consequently, the most similar molecule in training set was identified, for each molecule in the test set. We plotted the histogram of the similarities between each test set member and its nearest member in the training set. Moreover, we calculated the

average and standard deviations of the calculated similarity values (see Fig. A1 and Table A1 and A2, below).

The distribution of similarities for test set molecules for the data related to the CDK1 active/inactive model, is shown in Fig. A1(a). The similarity values for this figure were calculated using 20 VIP-selected descriptors. As can be seen in this figure, 160 out of 438 molecules in the test sets had similarities below 0.6 which comprises 36.52% of the whole test set for the binary active/inactive classifier for CDK1. This model shows an error rate of 0.18 in the test set, emphasizing many molecules in the test set with not much similarity to the training set are truly predicted by the model. It shows the robustness of the developed model even for molecules which are dissimilar to the training set. Further analysis using 208 descriptors (Fig. A1(g)) revealed significantly lower similarity values, indicating greater molecular diversity in expanded descriptor space. The observed decrease in similarity with the expanded descriptor space underscores the distinctiveness of the molecules in the test set.

As another example, the distribution of the similarity values in the space of 23 VIP-selected descriptors for the test set molecules for binary active-inactive classifier for CDK2 is shown in Fig A1(b). As can be seen in this figure, 334 out of 677 molecules in the test set have similarity values less than 0.6. These molecules comprise 49.33% of the whole test set. The error rate for the binary active/inactive classifier for CDK2 molecules is 0.13 for the test set and it implies that many dissimilar molecules in the test set were truly predicted. Further investigation using 207 descriptors, as shown in Fig. A1(h), revealed significantly lower similarity values, with 99.11% of the molecules being dissimilar (with a similarity threshold of 0.6). This indicates inherent differences among the molecules.

Here, we restrain from discussing all figures for the sake of brevity and emphasize that the observed patterns for most of the models were quite similar. We could observe that always in all models the percentages of dissimilar molecules in the test set (similarity threshold below 0.5~0.6) were much higher than the error rate of the models. It shows that the models could truly predict a remarkable portion of dissimilar molecules in the test sets.

Appendix A

Fig. A1. The histogram of the distribution of maximum similarities based on Euclidean distance between test set member and their nearest training set member for (a) active/inactive CDK1 model, (b) active/inactive CDK2 model, (c) active/inactive CDK4 model, (d) active/inactive CDK5 model (e) active/inactive CDK9 model (f) multi-class classification model for actives. The distributions in (a-f) were calculates using 20, 23, 17, 25, 21, and 31 VIP-selected molecular descriptors, respectively. The distributions in (a-f) were calculates using 208, 207, 195, 205, 204, and 198 correlation-filtered molecular descriptors, respectively.

Appendix A

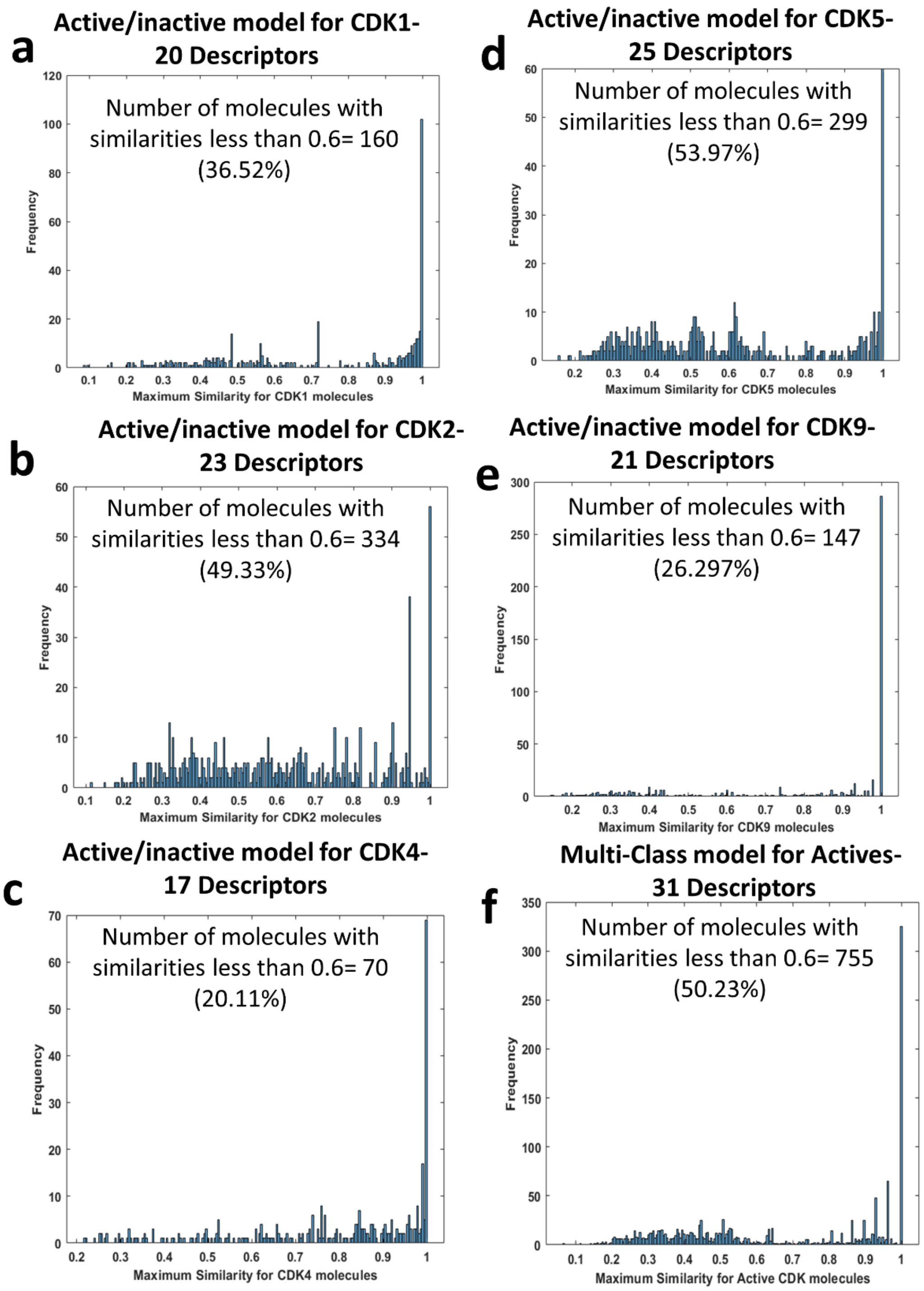

a
Active/inactive model for CDK1-
20 Descriptors
Number of molecules with
similarities less than 0.6= 160
(36.52%)
Frequency
Maximum Similarity for CDK1 molecules
b
Active/inactive model for CDK2-
23 Descriptors
Number of molecules with
similarities less than 0.6= 334
(49.33%)
Frequency
Maximum Similarity for CDK2 molecules
c
Active/inactive model for CDK4-
17 Descriptors
Number of molecules with
similarities less than 0.6= 70
(20.11%)
Frequency
Maximum Similarity for CDK4 molecules
d
Active/inactive model for CDK5-
25 Descriptors
Number of molecules with
similarities less than 0.6= 299
(53.97%)
Frequency
Maximum Similarity for CDK5 molecules
e
Active/inactive model for CDK9-
21 Descriptors
Number of molecules with
similarities less than 0.6= 147
(26.297%)
Frequency
Maximum Similarity for CDK9 molecules
f
Multi-Class model for Actives-
31 Descriptors
Number of molecules with
similarities less than 0.6= 755
(50.23%)
Frequency
Maximum Similarity for Active CDK molecules

Appendix A

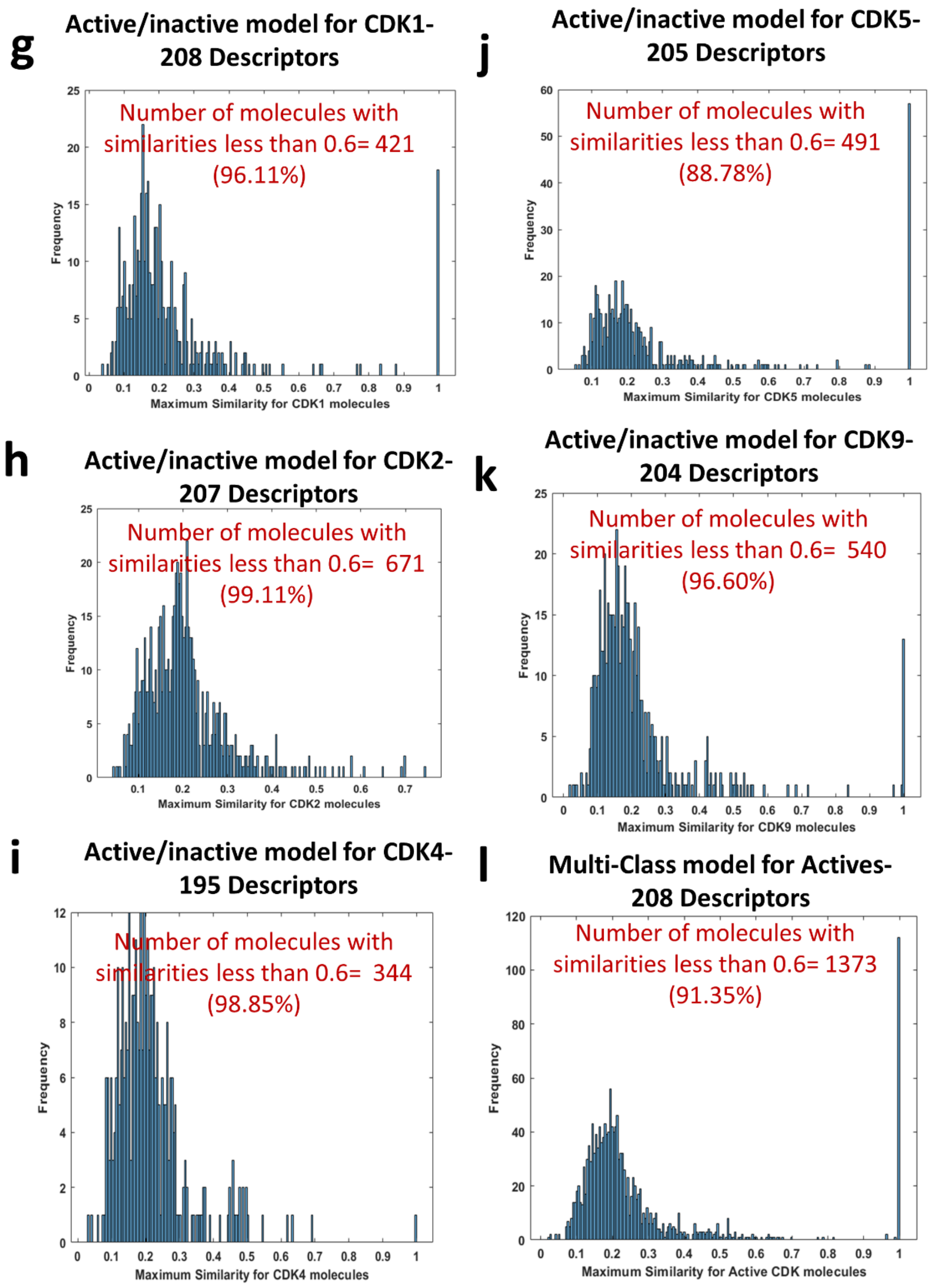

g
Active/inactive model for CDK1-208 Descriptors
Number of molecules with similarities less than 0.6= 421 (96.11%)
Frequency
Maximum Similarity for CDK1 molecules
j
Active/inactive model for CDK5-205 Descriptors
Number of molecules with similarities less than 0.6= 491 (88.78%)
Frequency
Maximum Similarity for CDK5 molecules
h
Active/inactive model for CDK2-207 Descriptors
Number of molecules with similarities less than 0.6= 671 (99.11%)
Frequency
Maximum Similarity for CDK2 molecules
k
Active/inactive model for CDK9-204 Descriptors
Number of molecules with similarities less than 0.6= 540 (96.60%)
Frequency
Maximum Similarity for CDK9 molecules
i
Active/inactive model for CDK4-195 Descriptors
Number of molecules with similarities less than 0.6= 344 (98.85%)
Frequency
Maximum Similarity for CDK4 molecules
l
Multi-Class model for Actives-208 Descriptors
Number of molecules with similarities less than 0.6= 1373 (91.35%)
Frequency
Maximum Similarity for Active CDK molecules

Appendix A

Table A1. The mean and standard deviation of maximum similarities based on Euclidean distance between test set members and their nearest training set member for CDK1-2, CDK4- CDK5, CDK9, and active CDK molecules by VIP-selected molecular descriptors.

Target	Active/Inactive Model for CDK1	Active/Inactive Model for CDK2	Active/Inactive Model for CDK4	Active/Inactive Model for CDK5	Active/Inactive Model for CDK9	Multi-Class Model for Actives
Average of maximum Similarity	0.7427	0.625	0.789	0.608	0.796	0.652
Std. of maximum Similarity	0.2698	0.245	0.221	0.252	0.280	0.284

Table A2. The mean and standard deviation of maximum similarities based on Euclidean distance between test set members and their nearest training set member for (a) CDK1, (b) CDK2, (c) CDK4, (d) CDK5 (e) CDK9 (f) active CDK molecules by filtered molecular descriptors.

Target	Active/Inactive Model for CDK1	Active/Inactive Model for CDK2	Active/Inactive Model for CDK4	Active/Inactive Model for CDK5	Active/Inactive Model for CDK9	Multi-Class Model for Actives
Average of maximum Similarity	0.044	0.204	0.213	0.297	0.216	0.278
Std. of maximum Similarity	0.1932	0.096	0.107	0.260	0.166	0.231

References:

1 Bajusz, D., Rácz, A. & Héberger, K. Why is Tanimoto index an appropriate choice for fingerprint-based similarity calculations? *Journal of Cheminformatics* **7**, 20, doi:10.1186/s13321-015-0069-3 (2015).

Appendix B

In order to validate the suggested SAR patterns in this work, we examined them by analyzing analogues of CDK molecules retrieved from MolPort database. We also collected the decoys and ligands of CDK2 target from directory of useful decoys (DUD database [1]) for evaluation of the suggested SAR for CDK2 target. The followings describe the detailed of the analysis procedure:

A) MolPort Analysis for CDK2: Ten molecules were randomly selected from the pool of the active CDK2 molecules. We searched MolPort for analogues of the selected molecules and ten sets of analogues were downloaded. Optimization of these analogues was performed using OpenBabel to enhance their suitability for further analysis. We calculated the molecular descriptors for the identified analogues using Dragon software. Then, we predicted the activity of analogues using our developed active/inactive binary classification models. This predictive analysis helps for ensuring about the activity level of the selected sets of analogues. After confirming the active analogues using the binary classification model, we filtered the active molecules based on SAR rules we obtained in our analysis. We considered higher number of nCbH for active CDK2 (nCbH>2). The filtered active analogues were used as inputs for our developed multiclass classification model in order to see if they show selectivity toward CDK2. The flowchart of the procedure is illustrated as Fig. B1. Moreover, the results of the models are summarized in Table B1. As shown in Table B1, from ten sets of analogues for active CDK2 molecules, eight sets were mostly predicted as active CDK2 using our previously developed binary active/inactive classifier. The predicted active CDK2 molecules were then used for further processing. In this step, we filtered these molecules with higher number of nCbH (nCbH>2). Then, the filtered molecules were used as inputs for multi-class classification model for target prediction. We hypnotize that, molecules with higher number of nCbH will show better activity toward CDK2 target. Among ten sets of active analogues analyzed in this work, nine sets with higher number of nCbH showed remarkable tendency toward CDK2 rather than other CDK targets (please see Table B1). The results in Table B1 are in agreement with the hypothesis.

B) MolPort Analysis for CDK4: The results of the analysis of analogues for CDK4 target are summarized in Table B2. For CDK4 target, we collected three sets of analogues from MolPort website. The collected analogues were used as input for the active/inactive classification model

for CDK4 target to confirm the activity of the molecules. The majority of the collected analogues for all three sets of analogues were predicted as active CDK4. Here, we hypothesized that the molecules with higher values of T(N..N) will show better selectivity for CDK4 target. We filtered the active molecules with higher T(N..N) values (greater than 180). The filtered molecules were used as inputs for the developed multi-class classification model. The majority of the filtered molecules showed selectivity toward CDK4 target which is in agreement with the hypotheses mentioned above.

We did not report more evaluation of observed SAR patterns for the sake of brevity, but our analysis on other rules also showed some similar patterns.

C) DUD analysis: For testing our models more, we decided to utilize the DUD. This database is specifically designed to test docking algorithms by providing challenging decoys. We downloaded the ligands and decoys of the CDK2 target from DUD. The ligands and decoys were not available at DUD for other CDK targets.

After downloading ligands and decoys for the CDK2, the structures were optimized using OpenBabel and the molecular descriptors were calculated using Dragon software. We took the 23 VIP-selected molecular descriptors (i.e. the same for active/inactive CDK2 classifier) for DUD ligands and decoys. As the next step, we predicted the activity of ligands and decoys, downloaded from DUD, using the active/inactive CDK2 model. Our working hypothesis posits that DUD ligands would align with the active class, while DUD decoys would align with the inactive class for CDK2 target. As evidenced in Table B3, across both the CPANN and SKN models, ligands predominantly aligned with the active CDK2 class.

The ligands were further utilized as inputs for a multiclass target prediction model employing 31 carefully VIP-selected descriptors. Notably, the results depicted in Table B3 highlight a notable predominance of ligands predicted as active CDK2 molecules compared to other classes for both SKN (76.36%) and CPANN (83.64 %) models. Table B4 further demonstrates a higher representation of decoys classified as inactives within the CDK2 class in both models.

Appendix B

We also compared the mean values of VIP-selected molecular descriptors across ligands and decoys in DUD dataset. This comparison assesses the alignment between the Binding-DB and DUD datasets to validate our results (see Table B5). An analysis of the 23 descriptors for active/inactive CDK2 model revealed that 16 descriptors (out of 23) for DUD molecules exhibited a consistent pattern with those of Binding-DB data (from the perspective of average values of descriptors within the active and inactive groups). This alignment supports the robustness of our models, indicating a correspondence between docking data and our results. The violin plots of the distribution of four molecular descriptors for ligand and decoy molecules in DUD database for CDK2 target are shown in Fig. B2. The ligands and decoys downloaded from the DUD database exhibit similar patterns to the active and inactive molecules taken from Binding-DB. These plots confirmed the consistency of our suggested rules across different datasets (see Fig. B2).

Appendix B

Fig B1. The flowchart of the procedure used for analyzing the analogues collected from MolPort database. a) for CDK2 analogues, b) for CDK4 analogues

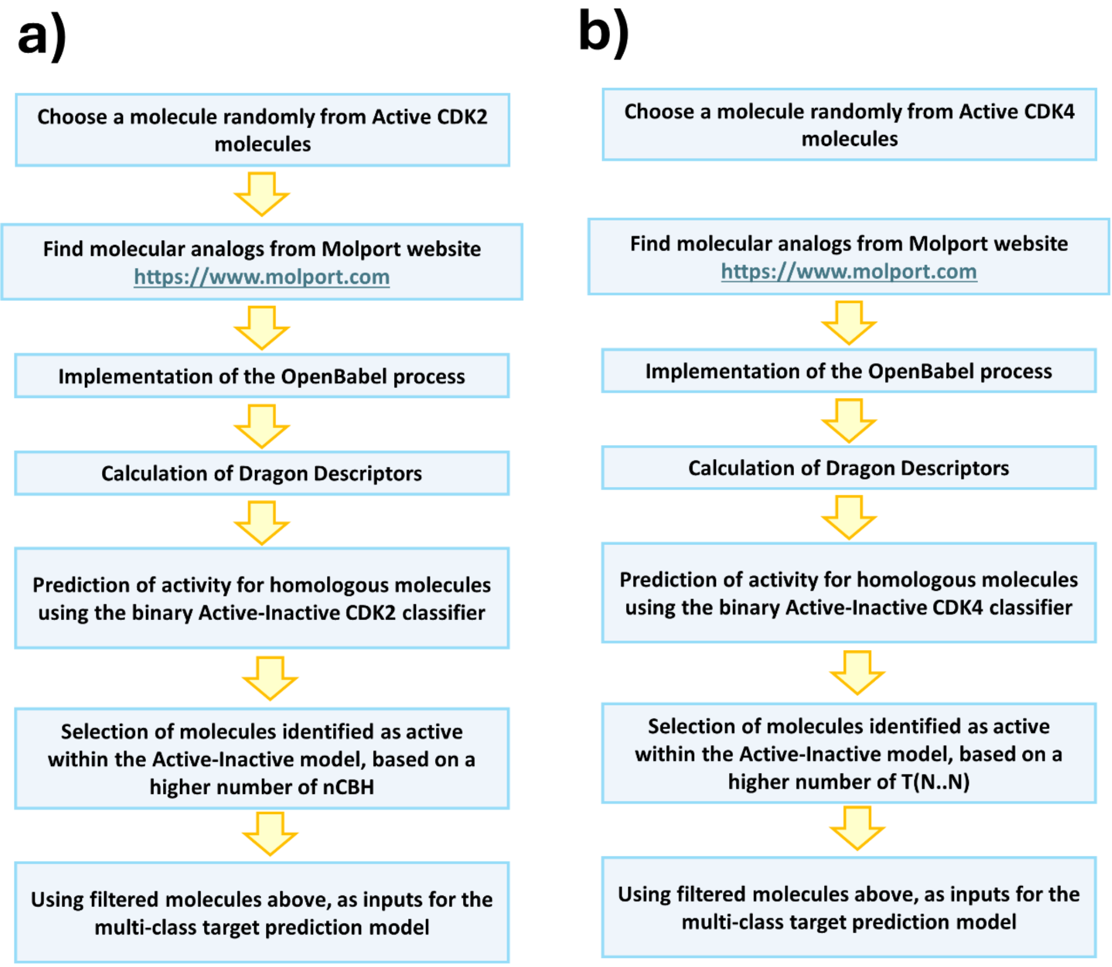

Appendix B

Table B1. The results of the analysis of analogues downloaded from MolPort database for the active CDK2 molecules. The analogues were predicted first using the binary active/inactive classifier and the confirmed molecules with higher number of nCbH were used as inputs for multi-class classifier for target prediction**.** The previously trained SKN models were used for the prediction procedure.

Analog Set Downloaded from MolPort	# Molecules in analogue Set	# Molecular Descriptors	Binary Active/Inactive Model's Output	# molecules in this class	Percentage predicted	Filtering based on high number of nCbH among predicted active CDK2 molecules	# Molecular Descriptors	Multi-Class Model's Output	# molecules in this class	Percentage predicted
1	98	23	Active CDK2	44	44.898%	26	31	Active CDK1	3	11.54%
								Active CDK2	11	42.31%
								Active CDK4	0	0.00%
			Inactive CDK2	54	55.102%			Active CDK5	3	11.54%
								Active CDK9	9	34.62%
2	12	23	Active CDK2	10	83.33%	10	31	Active CDK1	1	10.00%
								Active CDK2	4	40.00%
								Active CDK4	1	10.00%
			Inactive CDK2	2	10.66%			Active CDK5	2	20.00%
								Active CDK9	2	20.00%
3	68	23	Active CDK2	49	72.05%	31	31	Active CDK1	1	3.23%
								Active CDK2	25	80.65%
								Active CDK4	0	0.00%
			Inactive CDK2	19	27.94%			Active	1	3.23%

Appendix B

								CDK5		
								Active CDK9	4	12.90%
4	77	23	Active CDK2	56	72.72%	56	31	Active CDK1	7	12.50%
								Active CDK2	36	64.29%
								Active CDK4	6	10.71%
			Inactive CDK2	21	27.27%			Active CDK5	4	7.14%
								Active CDK9	3	5.36%
5	74	23	Active CDK2	54	72.97%	54	31	Active CDK1	3	5.56%
								Active CDK2	46	85.19%
								Active CDK4	0	0.00%
			Inactive CDK2	20	27.02%			Active CDK5	0	0.00%
								Active CDK9	5	9.26%
6	45	23	Active CDK2	24	53.33%	24	31	Active CDK1	8	33.33%
								Active CDK2	10	41.67%
								Active CDK4	0	0.00%
			Inactive CDK2	21	46.66%			Active CDK5	1	4.17%
								Active CDK9	5	20.83%
7	71	23	Active CDK2	39	54.92%	39	31	Active CDK1	6	15.38%
								Active CDK2	24	61.54%
								Active CDK4	2	5.13%
			Inactive CDK2	32	45.07%			Active CDK5	3	7.69%
								Active CDK9	4	10.26%

Appendix B

8	64	23	Active CDK2	30	46.87%	53	31	Active CDK1	10	33.33%
								Active CDK2	15	40.00%
								Active CDK4	0	0.00%
			Inactive CDK2	34	53.12%			Active CDK5	0	0.00%
								Active CDK9	5	16.67%
9	76	23	Active CDK2	44	54.92%	39	31	Active CDK1	6	15.38%
								Active CDK2	27	61.54%
								Active CDK4	2	5.13%
			Inactive CDK2	32	45.07%			Active CDK5	3	7.69%
								Active CDK9	4	10.26%
10	57	23	Active CDK2	34	59.64%	57	31	Active CDK1	12	35.29%
								Active CDK2	13	38.23%
								Active CDK4	0	0.00%
			Inactive CDK2	23	40.35%			Active CDK5	0	0.00%
								Active CDK9	9	26.47%

Appendix B

Table B2. The results of the analysis of analogues downloaded from MolPort database for the active CDK4 molecules. The analogues were predicted first using the binary active/inactive classifier and the confirmed molecules with higher number of T(N..N) were used as inputs for multi-class classifier for target prediction**.** The previously trained SKN models were used for the prediction procedure.

Analog Set Downloaded from MolPort	# Molecules in analogue Set	# Molecular Descriptors	Binary Active/Inactive Model's Output	# molecules in this class	Percentage predicted	Filtering based on high number of T(N..N) among predicted active CDK4 molecules	# Molecular Descriptors	Multi-Class Model's Output	# molecules in this class	Percentage predicted
1	35	17	Active CDK4	29	82.85%	2	31	Active CDK1	0	0.00%
								Active CDK2	0	0.00%
								Active CDK4	2	100.00%
			Inactive CDK4	6	17.14%			Active CDK5	0	0.00%
								Active CDK9	0	0.00%
2	37	17	Active CDK4	29	82.85%	5	31	Active CDK1	0	0.00%
								Active CDK2	0	0.00%
								Active CDK4	3	100.00%
			Inactive CDK4	8	17.14%			Active CDK5	0	0.00%
								Active CDK9	0	0.00%
2	93	17	Active CDK4	29	100.00%	4	31	Active CDK1	0	0.00%
								Active CDK2	1	25.00%
								Active CDK4	2	50.00%
			Inactive CDK4	8	0.00%			Active CDK5	1	25.00%
								Active CDK9	0	0.00%

Appendix B

Fig B2. The beeswarm plot **(a, e)** for C-034 **(b, f)** for Ui **(c, g)** for nR09 **(d, h)** Hy molecular descriptor(s) for the ligands and decoys of CDK2 target sourced from DUD (top row) and for the active and inactive groups of CDK2 molecules sourced from Binding-DB (bottom row).

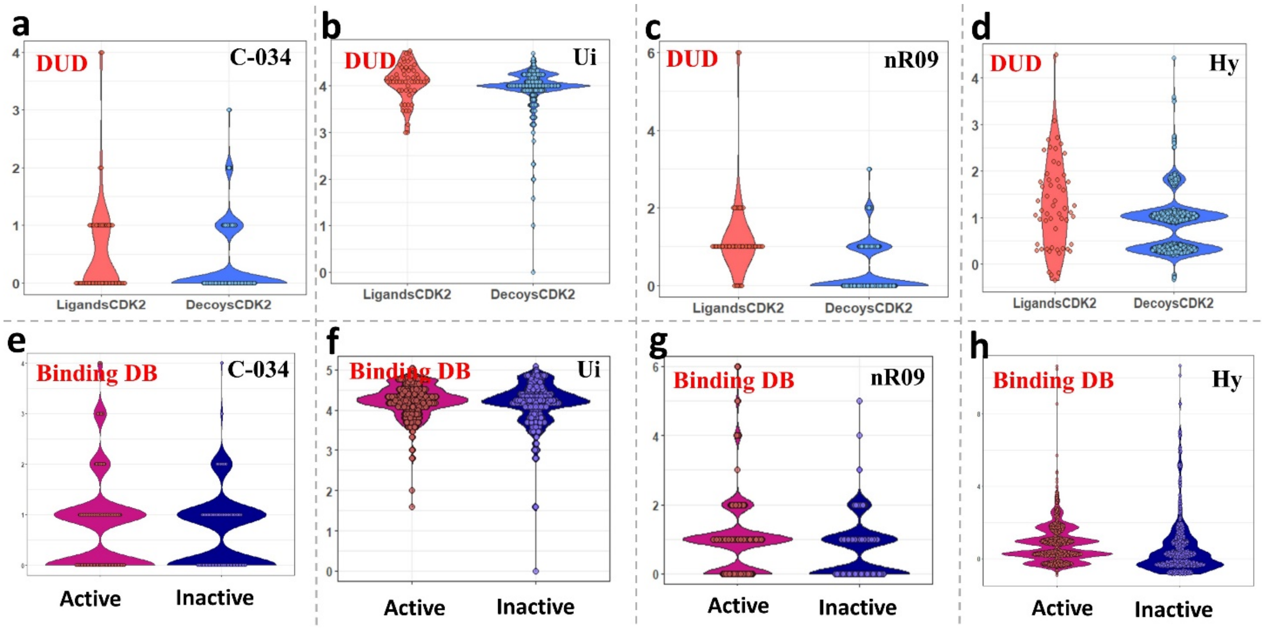

Appendix B

Table B3. The results obtained for prediction of ligands of CDK2 collected from DUD database using models developed by Binding-DB molecules. The ligands were firstly predicted using binary active/inactive SKN and CPANN classifiers (left side). Then, the ligands were also predicted using multi-class SKN and CPANN classifiers (right side). All models show higher percentages of prediction for active CDK2 group.

Model	# ligands downloaded from DUD	Number of Descriptors	Class	# ligands predicted in this class	Percentage predicted	# ligands downloaded from DUD	Number of Descriptors	Class	# ligands predicted in this class	Percentage predicted
SKN	55	23	Active CDK2	42	76.36%	55	31	Active CDK1	3	5.45%
								Active CDK2	32	58.18%
								Active CDK4	11	20.00%
			Inactive CDK2	13	23.64%			Active CDK5	4	7.27%
								Active CDK9	5	9.09%
CPANN	55	23	Active CDK2	46	83.64%	55	31	Active CDK1	8	14.55%
								Active CDK2	23	41.82%
								Active CDK4	15	27.27%
			Inactive CDK2	9	16.36%			Active CDK5	3	5.45%
								Active CDK9	6	10.91%

Appendix B

Table B4. The results obtained for prediction of decoys of CDK2 collected from DUD database using models developed by Binding-DB molecules. The decoys were predicted using binary active/inactive SKN and CPANN classifiers. Both models show higher percentages of prediction for inactive CDK2 group.

Model	# ligands downloaded from DUD	Number of Descriptors	Class	# ligands predicted in this class	Percentage predicted
SKN	1858	23	Active CDK2	739	39.7%
			Inactive CDK2	1119	60.2%
CPANN	1858	23	Active CDK2	500	26.9%
			Inactive CDK2	1358	73.0%

Appendix B

Table B5. The mean and std values of molecular descriptors for ligands and decoys of CDK2 molecules sourced from DUD website.

Selected molecular descriptors	Mean value for Ligands of CDK2 sourced from DUD	Std. value for Ligands of CDK2 sourced from DUD	Mean value for Decoys of CDK2 sourced DUD	Std. value for Decoys of CDK2 sourced DUD
C-029	0.454545	0.603023	0.144779	0.417737
O-058	1.436363	1.150904	2.038751	0.940104
nDB	1.763636	1.514909	2.754574	1.204481
qnmax	-0.4169636	0.081996	-0.435106	0.082054
F-084	0.127272	0.432672	0.036598	0.187824
C-034	0.527273	0.878858	0.30247578	0.587983
H-046	2.618182	3.729219	2.136706	2.759746
C-035	0.018182	0.13484	0.066200	0.248699
nS	0.418182	0.629253	0.482777	0.563639
Ui	4.067764	0.397819	3.989869	0.320925
TPSA(Tot)	103.818200	27.69615	106.467427	15.36433
O-060	0.581818	0.533712	0.788482	0.862013
S-110	0.236364	0.428764	0.201291	0.401073
Qmean	0.110436	0.014571	0.110708	0.017521
nRCONHR	0.309091	0.466378	0.379440	0.577581
nSO2N	0.200000	0.403687	0.185683	0.388956
T(N..S)	9.436364	15.7348	4.773412	7.171325
nR09	1.200000	1.078408	0.378363	0.603798
C-039	0.290900	0.458368	0.080731	0.289737
nArOR	0.527273	0.503857	0.531754	0.767763
T(O..F)	1.490909	5.252416	1.013993	5.588165
nCconj	1.218182	1.300867	1.726049	1.648021
Hy	1.278655	0.965401	0.841324	0.541952

Appendix B

1 Huang, N., Shoichet, B. K. & Irwin, J. J. Benchmarking Sets for Molecular Docking. *Journal of Medicinal Chemistry* **49**, 6789-6801, doi:10.1021/jm0608356 (2006).
